# Prevalence of mutations associated with artemisinin partial resistance and sulfadoxine–pyrimethamine resistance in 13 regions in Tanzania in 2021: a cross-sectional survey

**DOI:** 10.1016/S2666-5247(24)00160-5

**Published:** 2024-10

**Authors:** Jonathan J Juliano, David J Giesbrecht, Alfred Simkin, Abebe A Fola, Beatus M Lyimo, Dativa Pereus, Catherine Bakari, Rashid A Madebe, Misago D Seth, Celine I Mandara, Zachary R Popkin-Hall, Ramadhan Moshi, Ruth B Mbwambo, Karamoko Niaré, Bronwyn MacInnis, Filbert Francis, Daniel Mbwambo, Issa Garimo, Frank Chacky, Sijenunu Aaron, Abdallah Lusasi, Fabrizio Molteni, Ritha J A Njau, Samwel L Nhiga, Ally Mohamed, Jeffrey A Bailey, Deus S Ishengoma

**Affiliations:** a Division of Infectious Diseases, University of North Carolina School of Medicine, University of North Carolina at Chapel Hill, Chapel Hill, NC, USA; b Curriculum of Genetics and Molecular Biology, University of North Carolina School of Medicine, University of North Carolina at Chapel Hill, Chapel Hill, NC, USA; c Institute for Global Health and Infectious Diseases, University of North Carolina School of Medicine, University of North Carolina at Chapel Hill, Chapel Hill, NC, USA; d Department of Epidemiology, Gillings School of Global Public Health, University of North Carolina at Chapel Hill, Chapel Hill, NC, USA; e Department of Pathology and Laboratory Medicine, Brown University, Providence, RI, USA; f National Institute for Medical Research, Dar es Salaam, Tanzania; g School of Life Sciences and Bioengineering, Nelson Mandela African Institute of Science and Technology, Arusha, Tanzania; h Department of Immunology and Infectious Diseases, Harvard T H Chan School of Public Health, Boston, MA, USA; i Infectious Disease and Microbiome Program, Broad Institute, Boston, MA, USA; j National Malaria Control Programme, Dodoma, Tanzania; k Swiss Tropical Public Health Institute, Dar es Salaam, Tanzania; l Department of Parasitology and Medical Entomology, Muhimbili University of Health and Allied Sciences, Dar es Salaam, Tanzania; m Department of Biochemistry, Kampala International University in Tanzania, Dar es Salaam, Tanzania

## Abstract

**Background:**

The emergence of the artemisinin partial resistance (ART-R) mutation in the *Plasmodium falciparum kelch13* gene (*k13*), Arg561His, in Rwanda and the regional presence of polymorphisms affecting sulfadoxine–pyrimethamine have raised concern in neighbouring Tanzania. The goal of this study was to assess the status of antimalarial resistance in Tanzania, with a focus on the border with Rwanda, to understand the distribution of the Arg561His mutation, partner drug resistance, and resistance to chemoprevention drugs.

**Methods:**

In this cross-sectional survey, capillary dried blood spots were collected from malaria positive asymptomatic individuals in the community and symptomatic individuals in health facilities aged 6 months and older, in 13 regions of mainland Tanzania from Jan 31 to June 26, 2021. Exclusion criteria included residence of the areas other than the target sites, presenting to the health facility for care and treatment of conditions other than malaria, and not providing informed consent. Samples were assessed for antimalarial resistance polymorphisms and genetic relatedness using molecular inversion probes targeting *P falciparum* and short-read whole-genome sequencing. The primary outcome was the prevalence of molecular markers of antimalarial resistance at the region level, as well as at the district level in Kagera, a region in the northwest of the country at the border with Rwanda.

**Findings:**

6855 (88·1%) of 7782 capillary dried blood spot samples collected were successfully genotyped. The overall prevalence of *k13* Arg561His in Kagera was 7·7% (90% CI 6·0–9·4; 50 of 649), with the highest prevalence in the districts near the Rwandan border (22·8% [31 of 136] in Karagwe, 14·4% [17 of 118]) in Kyerwa, and 1·4% [two of 144] in Ngara). *k13* Arg561His was uncommon in the other regions. Haplotype analysis suggested that some of these parasites are related to isolates collected in Rwanda in 2015, supporting regional spread of Arg561His. However, a novel *k13* Arg561His haplotype was observed, potentially indicating a second origin in the region. Other validated *k13* resistance polymorphisms (one Arg622Ile and two Ala675Val isolates) were also identified. A region of prevalent dihydrofolate reductase Ile164Leu mutation, associated with sulfadoxine–pyrimethamine resistance, was also identified in Kagera (15·2% [12·6–17·8%]; 80 of 526). The mutant *crt* Lys76Thr mutation, associated with chloroquine and amodiaquine resistance, was uncommon, occurring only in 75 of 2861 genotyped isolates, whereases the wild-type *mdr1* Asn86Tyr allele, associated with reduced sensitivity to lumefantrine, was found in 99·7% (3819 of 3830) of samples countrywide.

**Interpretation:**

These findings show that the *k13* Arg561His mutation is common in northwest Tanzania and that multiple emergences of ART-R, similar as to what was seen in southeast Asia, have occurred. Mutations associated with high levels of sulfadoxine–pyrimethamine resistance are common. These results raise concerns about the long-term efficacy of artemisinin and chemoprevention antimalarials in the region. Understanding how multiple emergences interact with drivers of regional spread is essential for combating ART-R in Africa.

**Funding:**

This study was funded by the Bill & Melinda Gates Foundation and the National Institutes of Health.

## Introduction

Resistance to antimalarial drugs is one of the greatest threats to global control of malaria and is of grave concern in Africa, where the vast majority of cases and deaths occur.^1^ Historically, the emergence and spread of chloroquine and sulfadoxine–pyrimethamine resistance resulted in the collapse of effective treatment of malaria with substantial increases in morbidity and mortality.^2^ Antimalarial combination therapies, consisting of an artemisinin derivative and a partner drug (artemisinin-based combination therapy [ACT]), are currently the recommended therapeutic options for treating uncomplicated *falciparum* malaria.^1^ Given gains in malaria control have already plateaued and are reversing in some countries, the emergence of artemisinin partial resistance (ART-R) in Africa could be a global public health disaster if partner drug resistance emerges in concert resulting in ACT failure as in southeast Asia.^3^

**Research in context**

**Evidence before this study**

We searched PubMed for research articles published from Jan 1, 2014, to Oct 31, 2023, using the search terms “Africa” and “Artemisinin resistance” and “R561H” or “A675V” or “R622I”, returning 32 studies. No language restrictions were applied to this search. The published literature shows the emergence and establishment of Arg561His, Ala675Val, and Arg622Ile, three validated *Plasmodium falciparum kelch13* (*k13)* mutations associated with artemisinin partial resistance (ART-R), in Africa. Large molecular studies of Ala675Val in Uganda and Arg622Ile in Eritrea and Ethiopia have defined the regional spread of these mutations. However, little data are available from studies from the past 15 years about the spread and origins of the Arg561His mutation in the Great Lakes region of east Africa. Detailed studies of the regions of Tanzania that border Rwanda have not been carried out since the mutation was first reported in Rwanda in 2020 in samples from 2014. These data are needed for malaria control programmes to define and implement strategies for controlling the spread of ART-R in Africa, and mitigating a potential global public health disaster and the potential obstacle to the ongoing elimination strategies.

**Added value of this study**

This study reports the first country-wide, large-scale analysis of molecular markers of antimalarial resistance in Tanzania, with a focus on the regions bordering Rwanda where the *k13* Arg561His mutation reached high frequency. Using 6855 *P falciparum* positive samples successfully sequenced using molecular inversion probes from across Tanzania, we show that the *k13* Arg561His mutation has become frequent in three out of the eight districts of Kagera region, bordering Rwanda and Uganda. Importantly, we provide evidence for the separate emergence of a new extended haplotype around Arg561His in Tanzania. This is the first evidence that multiple independent emergences of the Arg561His ART-R have occurred in Africa, as was seen within the past two decades in southeast Asia. A region of high sulphadoxine-pyrimethamine drug resistance was also identified in Kagera with a high prevalence of dihydrofolate reductase Ile164Leu.

**Implications of all the available evidence**

These findings highlight that, similar to Arg622Ile and Ala675Val in other parts of Africa, we can expect the Arg561His mutation to continue to spread in the region. Additionally, this study highlights that we need to be watchful for new origins of mutations beyond the spread of existing resistant parasite lineages. ART-R appears to now be well established in multiple areas in eastern Africa. Intensive control in these regions to prevent spread and monitoring for partner drug resistance emergence in affected areas will be critical for preventing further reversal of malaria control efforts in the region and to support progress to the elimination targets by 2030.

Over a decade has passed since the emergence of ART-R in southeast Asia’s *Plasmodium falciparum* populations, leading to decreased drug efficacy.^4^ Clinical ART-R was first shown in the late 2000s in studies conducted in western Cambodia.^5^ The emergence of ART-R in western Cambodia set the stage for the eventual failure of ACTs, as resistance to the partner drugs also emerged, indicated by increasing treatment failures, parasite clearance times, and partner drug in-vitro resistance over a short timeframe. Early in the emergence of ACT resistance, many areas of southeast Asia had around 50% failure of treatment in patients treated with ACTs due to the combined effect of artemisinin and partner drug resistance.^6^

Mutations in the *P falciparum kelch13* gene (*k13*) are the key mediator of ART-R. These mutations were originally identified through drug pressure experiments and validated in the field and by genetic engineering studies.^7^^,^^8^ These mutations are thought to alter ubiquitination and help parasites resist accumulation of polyubiquitinated proteins and decrease haemoglobin uptake at the cytostome, leading to decreased artemisinin activation in the food vacuole.^9^ 13 key mutations in the *k13* propeller domain are now WHO-validated markers of ART-R (appendix p 4), with nine more mutations considered candidates or associated markers.^4^ Unfortunately, *k13* mutations have now been found extensively in eastern Africa, particularly in the Horn of Africa (Arg622Ile), Uganda (Cys469Tyr and Ala675Val), Rwanda (Arg561His), and Tanzania (Arg561His).^10^, ^11^, ^12^, ^13^, ^14^

In Ethiopia, the Arg622Ile mutation was first reported in 2014.^15^ Since then, these mutations have spread across Eritrea and Ethiopia.^11^^,^^12^ In Uganda, longitudinal molecular surveillance at 16 sites has documented spread of the Cys469Tyr and Ala675Val variants within Uganda.^10^ Importantly, all three of these mutations have been associated with either the clinical phenotypes of prolonged parasite clearance or day 3 positivity by microscopy, or extended parasite survival in vitro.^11^^,^^12^^,^^16^

The emergence and spread of the Arg561His mutation in the Great Lakes region of east Africa is not yet as clear when compared to other mutations. Originally described in Rwanda in samples from 2014 and 2015, this mutation appears to have increased in prevalence within the country over the past decade.^14^^,^^17^ In 2015, 7·4% of samples collected in Masaka had the Arg561His mutation^14^ which increased to 19·6% in Masaka and 22·0% in Rukara during a therapeutic efficacy study in 2018.^18^ Masaka met the WHO criteria for endemic ART-R as defined by more than 5% of patients infected with parasites carrying a *k13* resistance-confirmed mutation and who had persistent parasitaemia by microscopy on day 3.^19^ Whole-genome sequencing (WGS) of isolates from 2015 also confirmed a single haplotype of Arg561His in Rwanda that was not of Asian origin, suggesting de-novo mutation within Africa.^14^

*k13* mutations should always be studied in conjunction with partner drug resistance loci because together they lead to clinical failures of ACTs.^7^ Further, mutations to drugs no longer used for therapy, but that remain in use for chemoprevention (eg, sulfadoxine–pyrimethamine), are also important to characterise for malaria control programmes.

The Molecular Surveillance of Malaria in Tanzania (MSMT) project was developed in 2020 by the National Institute for Medical Research (NIMR) to provide nationwide longitudinal surveillance of parasite populations to understand key aspects of parasite biology that might impact malaria control and interventions. The emergence of Arg561His is a prime concern for Tanzania given its proximity to Rwanda and previous studies in Tanzania documenting occasional individuals harbouring mutant parasites, two in the Chato district and one on the eastern coast.^13^^,^^20^ In this study, we used samples collected through the MSMT to characterise the distribution of markers of the *k13* Arg561His mutation and of markers of resistance to partner drugs and chemoprevention drugs in Tanzania, particularly on the border with Rwanda.

## Methods

### Study design and participants

**Figure 1:**
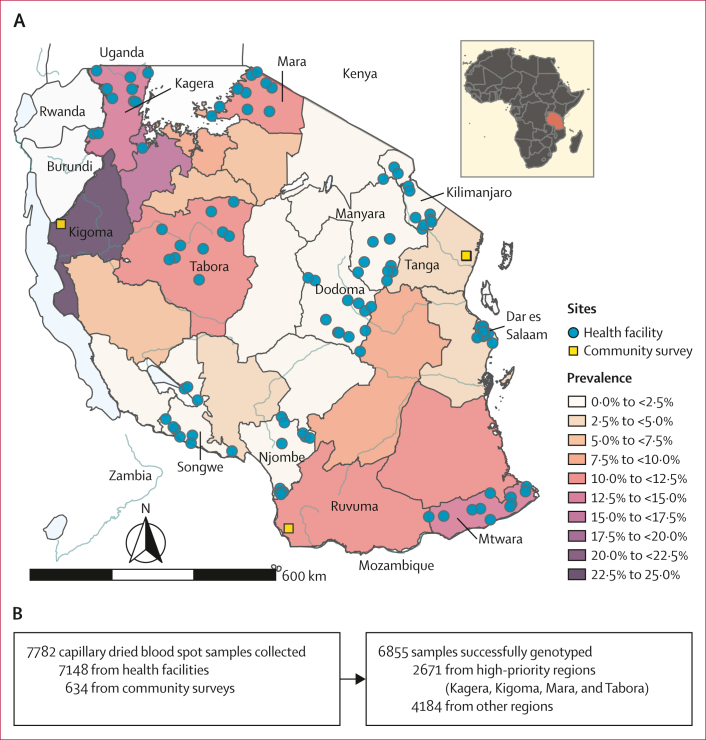
Tanzania malaria prevalence and study design (A) Map of Tanzania showing malaria prevalence in children aged 6–59 months in 2017 (National Malaria Control Program data). Health facilities where sampling occurred for this study are shown as circles and cross-sectional community survey collection sites are shown as squares. (B) Study profile showing the number of samples collected at health facilities and cross-sectional community surveys and the success of genotyping.

The MSMT project conducted cross-sectional surveys of asymptomatic individuals in communities and symptomatic patients in health facilities in 13 regions of mainland Tanzania from Jan 31 to June 26, 2021. MSMT collected capillary dried blood spot samples from patients aged 6 months and older with a positive malaria rapid diagnostic test. Exclusion criteria included residence of the areas other than the target sites, presenting to the health facility for care and treatment of conditions other than malaria, and not providing informed consent. These surveys involved 100 health facilities in ten regions (Dar es salaam, Dodoma, Kagera, Kilimanjaro, Manyara, Mara, Mtwara, Njombe, Songwe, and Tabora) and community surveys, in three additional regions (Kigoma, Ruvuma, and Tanga; figure 1A; appendix p 33). Details of the study sites’ selection, participant selection, and overall sampling are summarised in the appendix (pp 2, 5) and are previously described.^21^, ^22^, ^23^ Four regions (Kagera, Mara, Tabora, and Kigoma) were deemed high-priority areas for antimalarial resistance surveillance due to their proximity to Rwanda and to their location in the Lake Zone of the country with higher transmission (figure 1). Written informed consent was obtained for each participant as approved by the Tanzanian Medical Research Coordinating Committee of NIMR and de-identified dried blood spot samples were processed at NIMR, Dar es Salaam, Tanzania, and Brown University, Providence, RI, USA. De-identified data were analysed at the University of North Carolina (UNC), Chapel Hill, NC, USA, as approved by the UNC Institutional Review Board.

### Procedures

DNA was extracted from the dried blood spot samples using a Chelex-Tween protocol, and molecular inversion probe (MIP) captures and sequencing were conducted as previously described and as described in the appendix (pp 2, 34) for detection of molecular markers of artemisinin partial resistance, and resistance to partner and chemoprevention drugs.^24^ Briefly, this technique uses short oligonucleotide probes with arms designed to hybridise to regions flanking the targeted regions of interest. After MIPs are hybridised, targets are captured by polymerase extension and ligation to form circularised closed products that are subsequently enriched by exonuclease removal of all the template DNA. Each molecular capture is labelled with a unique molecular identifier (UMI). The captures are converted into a sequenceable library by PCR with Illumina specific primers incorporating sample-specific barcodes. This study used a panel specific for drug resistance polymorphism detection and a panel to look at genomic diversity.^24^ Approximately 2000–3000 samples were run together on each NextSeq 500 (Illumina, San Diego, CA, USA) run at 2 × 150 base pair read length, and sample libraries containing fewer than 10× reads at *k13* Arg561His were re-pooled to increase the amount of library for low coverage samples and resequenced. For samples from the high-priority regions (figure 1), we performed an additional MIP capture, using the same approach as described earlier, but with a smaller pool of MIP probes to capture high priority antimalarial resistance genes (appendix p 4), and resequencing to a high depth.^24^ Controls for each MIP capture and sequencing included DNA from 3D7 and 7G8 *P falciparum* genomes as well as no-template and no-probe controls.

Variant calling was conducted as previously described (appendix p 3).^24^ Briefly, fastq data were processed in MIPtools to generate and variants were called using freebayes.^24^ Downstream filtering exploited the UMI clustering for error correction and to enable high-confidence calls and quantification. We kept samples that had at least one haplotype that mapped in the expected locations of the genome for any of our drug resistance MIPs. For known drug resistance mutations (appendix p 4), antimalarial resistance prevalence was calculated for all variants with a UMI count of three or greater and, if heterozygous, with the alternate allele having one UMI or greater using a custom Python script. Candidate and validated antimalarial resistance mutations (appendix pp 9–32) were analysed using a UMI count of ten, with an alternate allele count of three and presence in at least three samples. Because each locus had independent success rates for genotyping, the denominator for prevalence estimates varies between loci. Maps were created using the sf package in R 4.2.1. Analysis of haplotypes included only samples for which complete genotypes across all involved loci were available. Inheritance by descent analysis of parasite relatedness among ART-R parasitaemias was done using the MIPAnalyser software (v.1.0.1) in R 4.2.1 as previously described.^24^

Analysis by WGS with selective whole-genome amplification (sWGA) products was attempted for 23 pure *k13* Arg561His, five mixed Arg561His, and 45 wild-type parasites based on MIP genotyping. This analysis included all available Arg561His mutant samples and location matched wild-type parasites. sWGA was performed in triplicate for each sample using a previously published protocol and pooled.^25^ The pooled sWGA product was sheared using a LE220R-plus Covaris Sonicator (Covaris, Woburn, MA, USA) and libraries prepared using dual indexing with the Kappa Hyper Prep Kit (Roche, Indianapolis, IN, USA). Pooled libraries were sequenced on a NovaSeq6000 (Illumina, San Diego, CA, USA) at the University of North Carolina High Throughput Sequencing Facility. We also downloaded publicly available WGS data (n=25) from *P falciparum* isolates collected in 2014–15 in Rwanda and 74 genomes with Arg561His from southeast Asia from the Pf7 archive, and 41 samples that passed quality filtering were included for joint variant calling with other samples (appendix p 7).^14^

Analysis of WGS data was done using the GATK4 pipeline following previously published methods.^26^ Briefly, reads were mapped to the 3D7 reference genome using BWA-mem, variants were called using GATK4, and single-nucleotide polymorphisms (SNPs) and indels were filtered using variant quality score recalibration. SNPs and indels passing filters were visualised in R version 4.2.1 using the gt package. To detect patterns of selection signals between the southeast Asia, Rwanda, and Tanzania haplotypes, we did extended-haplotype homozygosity statistics focusing on the Arg561His drug resistance SNP using filtered biallelic SNPs and with low missingness data from a variant call format file. All associated extended-haplotype homozygosity calculations were carried out using the R-package rehh (version 2.0.4).

### Outcomes

The primary outcome of this study was the determination of the prevalence of molecular markers of antimalarial resistance at the district and region level in Kagera and the region level in other parts of Tanzania. We included markers for ART-R and resistance to partner, and drugs used for chemoprevention: Lys76Thr in the chloroquine resistance transporter gene (*crt*); Asn51Ile, Cys59Arg, Ser108Asn, and Ile164Leu in the dihydrofolate reductase gene (*dhfr*); Ala437Gly, Lys540Glu and Ala581Gly in the dihydropteroate synthase gene (*dhps*); Arg561His, Arg622Ile, and Ala675Val in *k13*; and Asn86Phe, Asn86Tyr, Tyr184Phe, and Asp1246Tyr in the multidrug resistance transporter 1 gene (*mdr1*). The secondary outcome was the detailed analysis of parasites bearing WHO validated *k13* mutations to determine haplotypes and clusters of closely related parasites.

### Statistical analysis

The normal approximation interval method was used to calculate the 90% CIs for mutation prevalences as implemented within the Python statsmodels module (version 0.14.1).

### Role of the funding source

The funders of the study had no role in study design, data collection, data analysis, data interpretation, or writing of the report.

## Results

**Figure 2:**
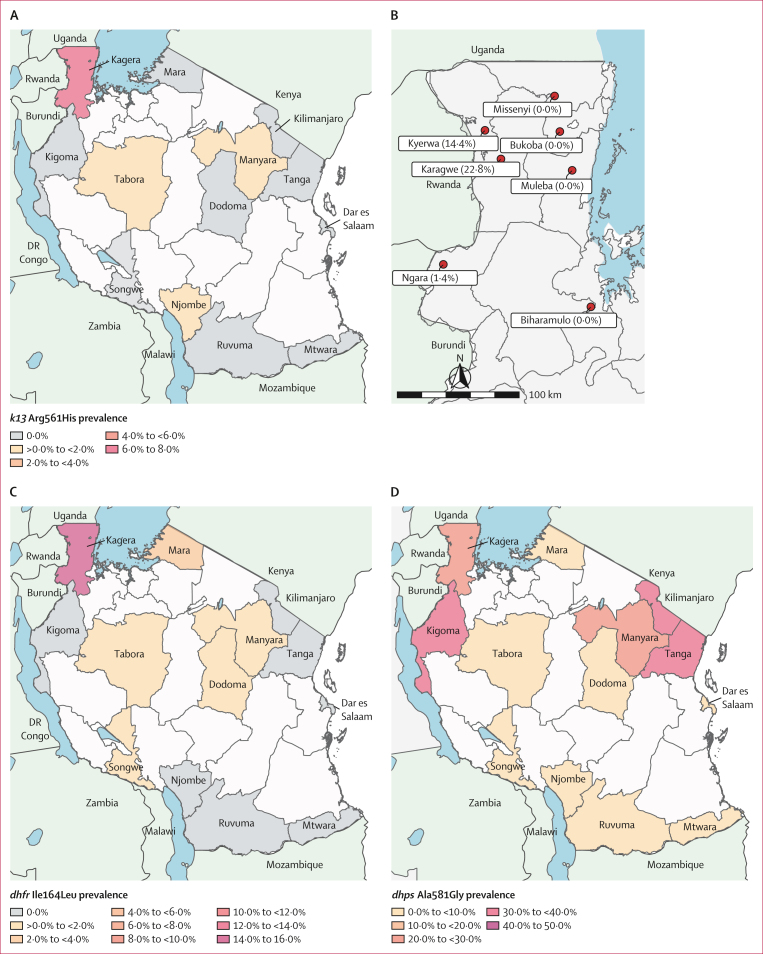
Antimalarial resistance polymorphisms in Tanzania (A) *k13* gene Arg561His mutations in Tanzania by region. White areas represent no sampling, grey areas represent sampled areas where no Arg561His was found, and coloured areas are shaded by the frequency of samples with the Arg561His mutation. (B) *k13* gene Arg561His mutations by district in Kagera. The districts with detected mutation all border Rwanda, with no mutations detected in eastern Kagera. The prevalence of *dhfr* Ile164Leu (C) and *dhps* Ala581Gly (D) in sampled regions is shown in colour gradients. Regions with no sampling are shown in white and regions where a polymorphism was not detected are shown in grey.

We successfully genotyped 6855 (88·1%) of 7782 capillary dried blood spot samples collected (figure 1B). Sequencing across the *k13* gene identified three WHO validated ART-R mutations: Arg561His (54 isolates), Arg622Ile (one isolate), and Ala675Val (two isolates). *k13* Arg561His was predominantly found in Kagera, the northwest region bordering Rwanda and Uganda, but was uncommon on a national scale (figure 2A; table). The overall prevalence in Kagera was 7·7% (50 of 649) and the highest prevalence was in districts in the west, near the Rwandan border: Karagwe at 22·8% (90% CI 16·9–28·7; 31 of 136), Kyerwa at 14·4% (9·1–19·7; 17 of 118), and Ngara at 1·4% (0·0–3·0; two of 144; figure 2B). Beyond Kagera, parasites with the Arg561His mutation were only detected at low prevalence in three other regions: Tabora at 0·5% (two of 438), Manyara at 0·6% (one of 179), and Njombe at 0·4% (one of 279), more than 800 km from the Rwanda border. *k13* Arg622Ile was found in a single isolate from Njombe in southwestern Tanzania (table 1). *k13* Ala675Val was found in one isolate from Kagera and one isolate from Tabora. Other *k13* propeller domain mutations not known to be associated with drug resistance were detected sporadically (appendix p 9). Outside the *k13* propeller domain, we found several polymorphisms including Lys189Thr in 20·6% (300 of 1460) of samples country-wide (appendix p 9). Other mutations that have putatively been associated with artemisinin partial resistance outside of *k13,* including *P falciparum* AP-2mu (AP2mu) are described in the appendix (pp 9–32).^27^ *P falciparum* coronin was not genotyped.

**Table:** Prevalence of antimalarial resistance mutations by region in Tanzania

	*crt* Lys76Thr	*dhfr* Asn51Ile	*dhfr* Cys59Arg	*dhfr* Ser108Asn	*dhfr* Ile164Leu	*dhps* Ala437Gly	*dhps* Lys540Glu	*dhps* Ala581Gly	*k13* Arg561His	*k13* Arg622Ile	*k13* Ala675Val	*mdr1* Asn86Phe	*mdr1* Asn86Tyr	*mdr1* Tyr184Phe	*mdr1* Asp1246Tyr
**Dar es Salaam**															
Number of resistant isolates∗	0/63	169/177	164/177	115/116	0/49	116/140	158/185	26/217	0/137	0/135	0/161	0/215	0/215	125/231	0/70
Estimated prevalence (90% CI)	0·0% (0·0–0·0)	95·5% (92·9–98·0)	92·7% (89·4–95·9)	99·1% (97·7–100·0)	0·0% (0·0–0·0)	82·9% (77·6–88·1)	85·4% (81·1–89·7)	12·0% (8·4–15·6)	0·0% (0·0–0·0)	0·0% (0·0–0·0)	0·0% (0·0–0·0)	0·0% (0·0–0·0)	0·0% (0·0–0·0)	54·1% (48·7–59·5)	0·0% (0·0–0·0)
**Dodoma**															
Number of resistant isolates∗	2/122	184/188	172/188	145/145	1/100	155/163	193/206	12/221	0/158	0/188	0/182	0/223	0/223	115/233	1/115
Estimated prevalence (90% CI)	1·6% (0·0–3·5)	97·9% (96·1–99·6)	91·5% (88·1–94·8)	100·0% (100·0–100·0)	1·0% (0·0–2·6)	95·1% (92·3–97·9)	93·7% (90·9–96·5)	5·4% (2·9–7·9)	0·0% (0·0–0·0)	0·0% (0·0–0·0)	0·0% (0·0–0·0)	0·0% (0·0–0·0)	0·0% (0·0–0·0)	49·4% (44·0–54·7)	0·9% (0·0–2·3)
**Kagera**															
Number of resistant isolates∗	39/586	681/690	630/690	632/632	80/526	651/692	654/697	189/729	50/649	0/709	1/680	1/711	3/711	325/731	18/610
Estimated prevalence (90% CI)	6·7% (5·0–8·3)	98·7% (98·0–99·4)	91·3% (89·5–93·1)	100·0% (100·0–100·0)	15·2% (12·6–17·8)	94·1% (92·6–95·6)	93·8% (92·3–95·3)	25·9% (23·3–28·6)	7·7% (6·0–9·4)	0·0% (0·0–0·0)	0·1% (0·0–0·4)	0·1% (0·0–0·4)	0·4% (0·0–0·8)	44·5% (41·4–47·5)	3·0% (1·8–4·1)
**Kigoma**															
Number of resistant isolates∗	12/86	94/94	86/94	88/88	0/88	92/92	87/87	32/99	0/73	0/94	0/100	0/75	0/75	31/92	4/99
Estimated prevalence (90% CI)	14·0% (7·8–20·1)	100·0% (100·0–100·0)	91·5% (86·8–96·2)	100·0% (100·0–100·0)	0·0% (0·0–0·0)	100·0% (100·0–100·0)	100·0% (100·0–100·0)	32·3% (24·6–40·1)	0·0% (0·0–0·0)	0·0% (0·0–0·0)	0·0% (0·0–0·0)	0·0% (0·0–0·0)	0·0% (0·0–0·0)	33·7% (25·6–41·8)	4·0% (0·8–7·3)
**Kilimanjaro**															
Number of resistant isolates∗	6/181	202/203	194/203	185/185	0/173	180/189	196/204	78/217	0/172	0/198	0/204	0/213	1/213	132/213	3/166
Estimated prevalence (90% CI)	3·3% (1·1–5·5)	99·5% (98·7–100·0)	95·6% (93·2–97·9)	100·0% (100·0–100·0)	0·0% (0·0–0·0)	95·2% (92·7–97·8)	96·1% (93·8–98·3)	35·9% (30·6–41·3)	0·0% (0·0–0·0)	0·0% (0·0–0·0)	0·0% (0·0–0·0)	0·0% (0·0–0·0)	0·5% (0·0–1·2)	62·0% (56·5–67·4)	1·8% (0·1–3·5)
**Manyara**															
Number of resistant isolates∗	1/128	216/218	211/218	162/162	1/91	169/183	200/218	55/242	1/179	0/202	0/202	0/236	0/236	138/243	2/117
Estimated prevalence (90% CI)	0·8% (0·0–2·1)	99·1% (98·0–100·0)	96·8% (94·8–98·8)	100·0% (100·0–100·0)	1·1% (0·0–2·9)	92·3% (89·1–95·6)	91·7% (88·7–94·8)	22·7% (18·3–27·2)	0·6% (0·0–1·5)	0·0% (0·0–0·0)	0·0% (0·0–0·0)	0·0% (0·0–0·0)	0·0% (0·0–0·0)	56·8% (51·6–62·0)	1·7% (0·0–3·7)
**Mara**															
Number of resistant isolates∗	3/555	574/581	538/581	563/566	17/558	551/568	563/579	5/590	0/535	0/578	0/585	0/556	1/556	372/584	15/554
Estimated prevalence (90% CI)	0·5% (0·0–1·1)	98·8% (98·1–99·5)	92·6% (90·8–94·4)	99·5% (99·0–100·0)	3·0% (1·8–4·2)	97·0% (95·8–98·2)	97·2% (96·1–98·4)	0·8% (0·2–1·5)	0·0% (0·0–0·0)	0·0% (0·0–0·0)	0·0% (0·0–0·0)	0·0% (0·0–0·0)	0·2% (0·0–0·5)	63·7% (60·4–67·0)	2·7% (1·6–3·8)
**Mtwara**															
Number of resistant isolates∗	0/97	160/186	176/186	107/107	0/77	126/157	165/210	1/239	0/137	0/144	0/170	0/238	0/238	145/252	1/92
Estimated prevalence (90% CI)	0·0% (0·0–0·0)	86·0% (81·8–90·2)	94·6% (91·9–97·3)	100·0% (100·0–100·0)	0·0% (0·0–0·0)	80·3% (75·0–85·5)	78·6% (73·9–83·2)	0·4% (0·0–1·1)	0·0% (0·0–0·0)	0·0% (0·0–0·0)	0·0% (0·0–0·0)	0·0% (0·0–0·0)	0·0% (0·0–0·0)	57·5% (52·4–62·7)	1·1% (0·0–2·9)
**Njombe**															
Number of resistant isolates∗	10/286	329/332	317/332	291/291	0/246	280/307	303/337	17/359	1/279	1/345	0/331	2/342	2/342	182/354	1/277
Estimated prevalence (90% CI)	3·5% (1·7–5·3)	99·1% (98·2–100·0)	95·5% (93·6–97·4)	100·0% (100·0–100·0)	0·0% (0·0–0·0)	91·2% (88·5–93·9)	89·9% (87·2–92·6)	4·7% (2·9–6·6)	0·4% (0·0–0·9)	0·3% (0·0–0·8)	0·0% (0·0–0·0)	0·6% (0·0–1·3)	0·6% (0·0–1·3)	51·4% (47·0–55·8)	0·4% (0·0–1·0)
**Ruvuma**															
Number of resistant isolates∗	0/56	65/65	64/65	60/60	0/46	51/54	64/69	2/72	0/38	0/68	0/68	0/63	0/63	42/78	0/41
Estimated prevalence (90% CI)	0·0% (0·0–0·0)	100·0% (100·0–100·0)	98·5% (96·0–100·0)	100·0% (100·0–100·0)	0·0% (0·0–0·0)	94·4% (89·3–99·6)	92·8% (87·6–97·9)	2·8% (0·0–6·0)	0·0% (0·0–0·0)	0·0% (0·0–0·0)	0·0% (0·0–0·0)	0·0% (0·0–0·0)	0·0% (0·0–0·0)	53·8% (44·6–63·1)	0·0% (0·0–0·0)
**Songwe**															
Number of resistant isolates∗	0/270	360/362	348/362	328/328	1/228	310/335	342/371	8/399	0/346	0/377	0/362	0/377	0/377	162/386	1/293
Estimated prevalence (90% CI)	0·0% (0·0–0·0)	99·4% (98·8–100·0)	96·1% (94·5–97·8)	100·0% (100·0–100·0)	0·4% (0·0–1·2)	92·5% (90·2–94·9)	92·2% (89·9–94·5)	2·0% (0·9–3·2)	0·0% (0·0–0·0)	0·0% (0·0–0·0)	0·0% (0·0–0·0)	0·0% (0·0–0·0)	0·0% (0·0–0·0)	42·0% (37·8–46·1)	0·3% (0·0–0·9)
**Tabora**															
Number of resistant isolates∗	2/381	504/509	480/509	436/437	6/376	459/472	506/519	20/555	2/438	0/498	1/464	0/521	1/521	280/538	21/411
Estimated prevalence (90% CI)	0·5% (0·0–1·1)	99·0% (98·3–99·7)	94·3% (92·6–96·0)	99·8% (99·4–100·0)	1·6% (0·5–2·7)	97·2% (96·0–98·5)	97·5% (96·4–98·6)	3·6% (2·3–4·9)	0·5% (0·0–1·0)	0·0% (0·0–0·0)	0·2% (0·0–0·6)	0·0% (0·0–0·0)	0·2% (0·0–0·5)	52·0% (48·5–55·6)	5·1% (3·3–6·9)
**Tanga**															
Number of resistant isolates∗	0/50	58/58	55/58	49/49	0/48	45/49	54/63	25/65	0/30	0/45	0/56	0/60	0/60	25/69	2/35
Estimated prevalence (90% CI)	0·0% (0·0–0·0)	100·0% (100·0–100·0)	94·8% (90·0–99·6)	100·0% (100·0–100·0)	0·0% (0·0–0·0)	91·8% (85·4–98·3)	85·7% (78·5–93·0)	38·5% (28·5–48·4)	0·0% (0·0–0·0)	0·0% (0·0–0·0)	0·0% (0·0–0·0)	0·0% (0·0–0·0)	0·0% (0·0–0·0)	36·2% (26·7–45·7)	5·7% (0·0–12·2)
**Overall**															
Number of resistant isolates∗	75/2861	3596/3663	3435/3663	3161/3166	106/2606	3185/3401	3485/3745	470/4004	54/3171	1/3581	2/3565	3/3830	8/3830	2074/4004	69/2880
Estimated prevalence (90% CI)	2·6% (2·1–3·1)	98·2% (97·8–98·5)	93·8% (93·1–94·4)	99·8% (99·7–100·0)	4·1% (3·4–4·7)	93·6% (93·0–94·3)	93·1% (92·4–93·7)	11·7% (10·9–12·6)	1·7% (1·3–2·1)	0·0% (0·0–0·1)	0·1% (0·0–0·1)	0·1% (0·0–0·2)	0·2% (0·1–0·3)	51·8% (50·5–53·1)	2·4% (1·9–2·9)

Prevalence reflects the alternate allele relative to the total number of samples successfully genotyped at each allele, because not every sample had genotyping at every allele. *crt*=chloroquine resistance transporter gene. *dhfr*=dihydrofolate reductase gene. *dhps*=dihydropteroate synthase gene. *k13*=kelch 13 gene. *mdr1*=multidrug resistance transporter 1 gene.

∗ Out of the total number successfully genotyped.

**Figure 3:**
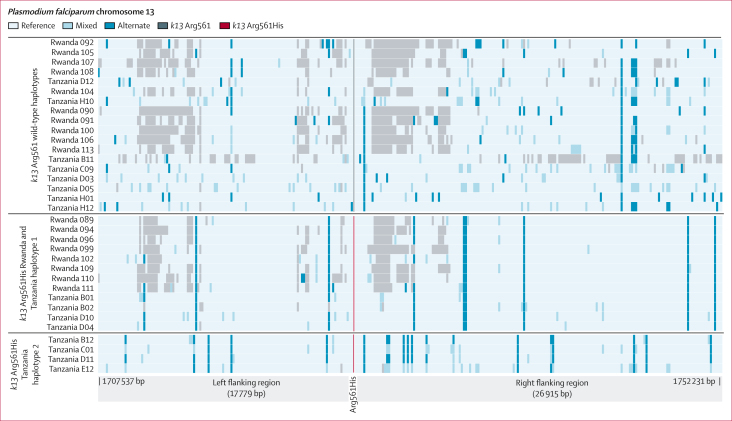
Extended flanking haplotype plot around *k13* Arg561His mutations Every SNP, insertion or deletion passing filters between 1 707 537 bp and 1 752 231 on chromosome 13 is presented as a coloured line, the SNP coding for pure mutant Arg561His is shown in red. All samples were jointly called for variation. Grey regions represent loci where a call could not be made due to low coverage. The top panel represents wild-type and mixed genotypes at position 561. The middle panel shows a common haplotype between Rwandan and Tanzanian isolates. The bottom panel shows a novel haplotype flanking Arg561His detected in Kagera. SNP=single-nucleotide polymorphism.

**Figure 4:**
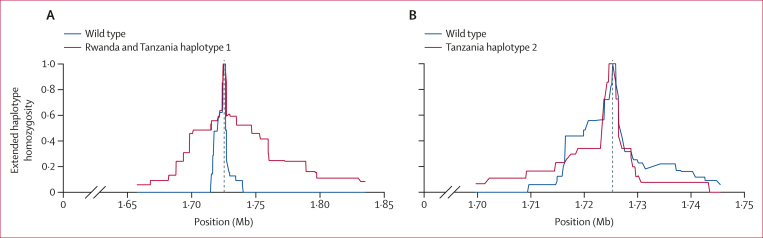
Extended-haplotype homozygosity analysis of Arg561His haplotypes (A) Extended-haplotype homozygosity for the Rwanda and Tanzania haplotype 1 Arg561His (n=12) compared to wild-type parasites. (B) Extended-haplotype homozygosity of the Tanzania haplotype 2 Arg561His (n=4) compared with wild-type parasites. The vertical dotted line represents the position of the single-nucleotide polymorphism for Arg561His. The x axes show the upstream and downstream genomic coordinates from the locus of interest and the homozygosity scale is shown on the y axes, ranging from 0 to 1 (0 representing no homozygosity and 1 representing complete homozygosity).

We achieved sufficient depth and quality on 29 (46%) of 63 Tanzanian isolates from 2021 (including 15 wild type, eight pure Arg561His, and six heterozygous) to assess the variation surrounding *k13*, and analysed them in combination with publicly available WGS data from the isolates collected in 2014 and 2015 in Rwanda and from southeast Asia.^14^ We identified a shared haplotype between the older Rwandan isolates and the contemporary Tanzanian isolates (n=4 from Tanzania; Rwanda and Tanzania haplotype 1), suggesting cross-border spread resulting from a single origin. However, a second extended haplotype (n=4; Tanzania haplotype 2) was also found within the Tanzanian parasites (figure 3). Rwanda and Tanzania haplotype 1 and Tanzania haplotype 2 differ at the closest flanking SNPs (within 1 kb of the Arg561His mutation). The Tanzania haplotype 2 also does not appear to be of southeast Asia origin (appendix p 35). Extended-haplotype homozygosity analysis showed extended haplotypes for both Rwanda and Tanzania haplotype 1 and Tanzania haplotype 2 relative to wild-type parasites, consistent with positive selection for both Arg561His haplotypes (figure 4). Inheritance by descent analysis showed that Arg561His isolates from Kagera were closely related in multiple small clusters (appendix p 36). The two isolates from Tabora were related to Kagera isolates (inheritance by descent ≥0·25), with the other two being more distant (appendix p 37).

Known partner drug resistance mutations were assessed (table). In Kagera, 39 of 586 successfully genotyped samples had the *crt* Lys76The mutation, which is associated with chloroquine and amodiaquine resistance, and 31 of these isolates were from Ngara, a district bordering Rwanda and Burundi. The wild-type *mdr1* Asn86Tyr allele, associated with reduced sensitivity to lumefantrine, was found in 99·7% (3819 of 3830) of samples countrywide. The *mdr1* Asn86Tyr, Tyr184Phe, and Asp1246Tyr haplotype, a haplotype that has been associated with decreased sensitivity to lumefantrine and selected for in population when the drug is used,^28^ was found in 52·6% (1420 of 2698) of samples countrywide. Mutations associated with ART-R in southeast Asia were uncommonly found (appendix p 2).

Markers for sulfadoxine–pyrimethamine resistance were also genotyped, revealing high frequency of many mutations and the emergence of *dhfr* Ile164Leu in Kagera (table 1; figure 2C). The folate synthesis gene triple mutations *dhfr* Asn51Ile, Cys59Arg, and Ser108Asn were found to be near fixation countrywide with this haplotype found at a prevalence of 92·5% (2893 of 3128). Notably, the *dhfr* Ile164Leu mutation was found at 15·2% (80 of 526) in the Kagera region, accounting for 4·2% (105 of 2524) of the Asn51Ile, Cys59Arg, Ser108Asn, and Ile164Leu haplotypes found in the country (figure 2C). In the *dhps* gene, the Lys540Glu mutation was found at 93·1% (3485 of 3745) in Tanzania with little geographical variation (table 1), and the prevalence of *dhps* Ala581Gly ranged from 0·4% (one of 239) in Mtwara to 38·5% (25 of 65) in Tanga (figure 2D). The *dhfr*–*dhps* sextuple mutation (*dhfr* Asn51Ile, Cys59Arg, Ser108Asn and *dhps* Ala437Gly, Lys540Glu, Ala581Gly), has been associated with the compromise of intermittent preventive treatment in pregnancy and was found in 11·2% (323 of 2894) of samples nationally.^29^

## Discussion

Here we show high frequencies of *k13* Arg561His in Kagera (7·7%), a region bordering Rwanda. The presence of the TZ1 haplotype, previously described in Rwanda, is consistent with spread of resistant parasites between Rwanda and Tanzania. More concerning is the evidence of a potential second origin of the Arg561His mutation (Tanzania haplotype 2) in Tanzania, suggesting that ART-R might follow patterns similar to those seen in southeast Asia, with multiple independent origins of the same mutation.^29^^,^^30^ Haplotype analysis suggests multiple origins, but does not prove the mechanism behind them.^10^ If multiple origins occur, containment efforts are more difficult, due to the need to closely monitor for new haplotypes and the spread of specific haplotypes, which might not become extensive without partner drug resistance. However, the presence of multiple haplotypes might be due to gene conversion or recombination events adjacent to the mutation.

Several mutations in the propeller domain of the *k13* gene that are now associated with delayed parasite clearance are present in east Africa.^10^^,^^11^ The most concerning of these, Cys469Tyr, Arg561His, Arg622Ile, and Ala675Val, have shown clinical and in-vitro validation of ART-R. Determining the exact origin of these mutations with certainty is difficult due to the patchy nature of the surveillance data, but one (Arg561His) of the three mutations appears to have originated on the Rwanda–Tanzania border. Arg561His was first detected in Rwanda in 2015 and later in DR Congo, Tanzania, and Uganda.^10^^,^^13^^,^^14^ To date, genotyping efforts to describe the status of *k13* mutations in Tanzania have been limited and do not capture the risk posed by ART-R parasites across the country. The high rate of human movement across the border with Rwanda, where people have close historical ties and One Stop Border Posts that routinely permit thousands of individuals and hundreds of vehicles to cross daily, is a primary concern for malaria control in Tanzania.^31^ Although there have been sporadic reports of validated ART-R polymorphisms in the past,^13^^,^^20^ systematic longitudinal surveillance of these mutations is necessary to identify areas at risk, as has been done in Uganda.^10^ The speed at which data are generated and reported to control programmes is crucial. The MSMT project will provide these nationwide data for Tanzania given the identified threat shown here of Arg561His in Kagera, by allowing yearly sampling and in-country sequencing and analysis of data as the project develops.

Partner drug resistance surveillance showed important patterns. An intriguing pattern of *crt* gene Lys76Thr mutations was found to be clustered near the Burundi border. A possible explanation is that the broad use of artesunate–amodiaquine in Burundi is contributing to continued selection for chloroquine or amodiaquine resistance and that these resistant parasites are being imported into Kagera. Given artemether–lumefantrine remains the first-line antimalarial in Tanzania, it is not surprising that *mdr1* wild-type allele Asn86Tyr was near fixation and the Asn86Tyr, Tyr184Phe, and Asp1246Tyr haplotype was very common, occurring in over 50% of samples.

Markers of elevated sulfadoxine–pyrimethamine resistance are increasing in Tanzania, which has potential impacts on the use of this drug combination as a chemopreventive antimalarial. The geographical distribution of the *dhps* gene Ala581Gly mutation in Tanzania has historically been limited to Tanga and the Lake regions.^32^^,^^33^ We confirm that this remains the case with little evidence of expansion into other regions of Tanzania. The reasons why this mutation has not spread in Tanzania remain unclear and warrant further evaluation, especially given the evidence that spread is occurring in the neighbouring DR Congo and Uganda.^34^^,^^35^ Previous work has suggested that multiple origins of parasites bearing the Ala581Gly mutation have occurred in east Africa, suggesting local origins and limited spread.^36^ However, that work was based on five microsatellites and might not take into account the complex stepwise evolutionary history of mutations in *dhps*. Importantly, a new focus of the quadruple mutation in the *dhfr* gene (Asn51Ile, Cys59Arg, Ser108Asn, and Ile164Leu) was identified in Kagera. In the laboratory, this form of the DHFR enzyme binds pyrimethamine 600 times less tightly than the wild type and about seven times less than the triple mutation Asn51Ile, Cys59Arg, and Ser108Asn.^37^ This weaker binding results in parasites being resistant to therapy in vitro at levels higher than what can be reached in vivo. Presence of *dhps* Ala581Gly has been associated with compromised intermittent preventive treatment in pregnancy and this mutation was commonly found in Tanzania, with the highest prevalence in Tanga.^29^ Given the regional high prevalence of *dhps* Ala581Gly and the emergence of *dhfr* Ile164Leu in northwest Tanzania, additional studies are warranted to evaluate the benefits of sulfadoxine–pyrimethamine-based chemoprevention in this region of Tanzania.

A major advantage of this study is the large geographical range and large number of samples available for analysis. However, this study design also engenders some limitations. The large number of samples resulted in overall low levels of coverage for sequencing in most regions (other than the four high-priority regions that received extra depth). The samples collected at the three community surveys performed poorly with our approach, probably due to low parasitaemia. The overall sequencing coverage in many regions might directly affect the ability to find minority drug-resistant variants in the data, and we might have filtered out minor variants at a within sample frequency of below 1%. Our analysis combines asymptomatic participants (576 of 6855 successfully genotyped) with symptomatic individuals, which might have resulted in an unknown bias. However, there are no known differences in the risk of infection with antimalarial-resistant parasites between these populations and asymptomatic participants represent a small fraction of the study group. Lastly, the study was not designed to be nationally representative, but represents more sites than in most studies and provides a broad estimate from these sites.

The detection of *k13* Arg561His at a prevalence of 22·8% in Karagwe district of Kagera, Tanzania, must raise alarms that ART-R is emerging and that ACTs efficacy is under threat. The independent origin of a new *k13* Arg561His extended haplotype raises a concern that multiple origins of ART-R mutations are emerging and spreading in Africa, complicating malaria control. Evolution of partner drug resistance must be monitored carefully, and therapeutic efficacy studies are urgently needed to understand the susceptibility of currently circulating *k13* mutations in the region.

## Data sharing

All sequencing data have been submitted to the Sequence Read Archive (PRJNA1090883 for molecular inversion probe data and PRJNA1092065 for WGS data). Other metadata are available from the corresponding author upon reasonable request. Code for analysis is available at https://github.com/bailey-lab/MSMT_2021_DR_analyses. The list of publicly available genomes used is provided in the appendix (pp 7–8).

## Declaration of interests

We declare no competing interests.
