# Supplementary material for: Prevalence of mutations associated with artemisinin partial resistance and sulfadoxine–pyrimethamine resistance in 13 regions in Tanzania in 2021: a cross-sectional survey
Source: Lancet Microbe. 2024 Oct;5(10):None. doi: 10.1016/S2666-5247(24)00160-5 (PMC11464622; doi:10.1016/S2666-5247(24)00160-5)
Supplement: Supplementary appendix [file mmc1.pdf]

THE LANCET
Microbe

Supplementary appendix

This appendix formed part of the original submission and has been peer reviewed.
We post it as supplied by the authors.

Supplement to: Juliano JJ, Giesbrecht DJ, Simkin A, et al. Prevalence of mutations associated with artemisinin partial resistance and sulfadoxine–pyrimethamine resistance in 13 regions in Tanzania in 2021: a cross-sectional survey. *Lancet Microbe* 2024. https://doi.org/10.1016/S2666-5247(24)00160-5

SUPPLEMENTAL APPENDIX

Prevalence of mutations associated with artemisinin partial resistance and sulfadoxine-pyrimethamine resistance in 13 regions in Tanzania in 2021: a cross-sectional survey

Table of Contents

Supplemental Text………………………………………………………………………. 2
Table S1………………………………………………………………………………….. 4
Table S2………………………………………………………………………………….. 5
Table S3………………………………………………………………………………….. 7
Table S4………………………………………………………………………………….. 9
Figure S1…………………………………………………………………………………… 33
Figure S2…………………………………………………………………………………… 34
Figure S3…………………………………………………………………………………… 35
Figure S4…………………………………………………………………………………… 36
Figure S5…………………………………………………………………………………… 37
References………………………………………………………………………………….. 38

SUPPLEMENTAL TEXT

Study Design

Cross-sectional sampling from 100 health facilities was undertaken according to the WHO protocol for surveillance of *Plasmodium falciparum* histidine rich proteins 2 and 3 (*pfhrp* 2/3) deletions.[1,2] Ten health facilities in each of the following regions were used: Dar es salaam, Dodoma, Kagera, Kilimanjaro, Manyara, Mara, Mtwara, Njombe, Songwe and Tabora. Patients that presented with symptoms compatible with uncomplicated malaria were approached and enrolled in the study. Written informed consent was provided by adult patients or parents/guardians for their children. Children aged 7 - 17 years also provided an assent. Enrollment criteria included being 6 months of age or older, meeting the case definition for uncomplicated malaria and residing in the study area. All clinic attendees who were enrolled in the study were provided with two types of malaria rapid diagnostic tests (mRDT), the standard histidine rich protein 2 (HRP2)/pan lactate dehydrogenase (LDH) mRDT used by the national health system and a *P. falciparum* LDH test [#C14RHG25, RapiGEN Inc. BIOCREDIT Malaria Ag Pf (Pf-pLDH), Republic of Korea], according to the WHO protocol and as described by Rogier et al.[2] Any patient with positive results by either of the two mRDTs was treated per national guidelines. In addition to mRDTs, all participants provided dried blood spots (DBS). These DBS were used to test for malaria using qPCR and sequencing. Participants who were mRDT were sequenced. In addition, a subset of qPCR positive mRDT negative were included in the study for sequencing.

In addition to health facility sampling, three cross-sectional community surveys were conducted in regions of Tanzania.[3] Malaria epidemiology in these regions has also been previously studied by the National Institute for Medical Research (NIMR).[4,5] Community samples were collected from 3 regions: Kigoma, Ruvuma, and Tanga. In these regions, asymptomatic community members or their guardians provided written consent (assent procedures were done as described above), were tested by an mRDT, and then provided DBS if positive for molecular analysis, including MIP sequencing.[3]

Participant characteristics are shown in **Table S2 p.5**. Age strata were designated based on previous reports.[1]

Molecular Inversion Probes

Molecular surveillance of mutations that impact antimalarial resistance (**Table S1 p. 4**) should rely on platforms that can broadly detect different resistance mutations. Highly multiplex amplicon deep sequencing is one approach that has shown promise. Another approach is molecular inversion probes (MIPs), which have now been used extensively to characterize drug resistance and population structure in parasites in Africa.[5–7] The ability to create and combine different highly multiplexed panels for antimalarial resistance mutations, copy number variation, gene deletions and other genome wide polymorphisms to study complexity of infection, parasite relatedness or population structure makes the MIP platform a highly flexible and cost-effective means of conducting malaria molecular surveillance (MMS). The methods used to produce the parasite genotyping reported in this study are summarized as follows (**Figure S2, p.34**): 1.) Chelex purified DNA template was hybridized to long oligos approximately 100 base pairs long, with each oligo containing complementary arms to the targeted capture region, as well as universal primer sequences and a unique molecular identifier (UMI). 2.) Following hybridization, a circularization step was performed using T4 ligase, then non circularized DNA was digested using exonucleases I and III. 3.) The pool of circularized capture sequence was then PCR amplified using universal primers containing illumina adapters to produce an amplicon that can be sequenced on the Illumina NextSeq500 platform (Illumina, San Diego, CA).

Amplicons from each sample contained a dual index that was demultiplexed using Illumina's built-in `bcl2fastq2` software (Illumina, San Diego, CA). Resulting fastq data was further processed in `MIPtools` and variants were called using `freebayes` (https://github.com/freebayes/freebayes). Downstream filtering exploited the UMI clustering to enable high-confidence calls. Samples with less than 10X coverage at K13 R561H, were rebalanced by re-pooling amplified products to increase the ratio of samples with low coverage and resequenced.

For the MSMT programme, we recognized that the high priority regions neighboring Rwanda needed additional sequencing effort. After an initial pass with all of the MIP probes included in each capture reaction, we found that sample missingness was relatively high. This led to a second round of MIP capture with a greatly reduced probe set designed to capture only key gene fragments associated with well described drug resistance phenotypes (**Table S1, p.4**).

Specific Whole Genome Amplification

Specific whole genome amplification (sWGA) is a technique that uses primers that more commonly or specifically bind to the pathogen of interest than to DNA in the background (e.g. human DNA). This method has been extensively used in malaria genomics to enrich parasite DNA from clinical samples to allow for whole genome sequencing. The sWGA reaction was carried out as previously described.[8] However, each sample was amplified in triplicate and the sWGA reactions pooled together prior to library preparation for whole genome sequencing.

Genomic architecture of artemisinin resistance polymorphisms from Southeast Asia

To investigate associations with mutations previously found to occur in drug resistant parasites in Southeast Asia in combination with K13 mutations, we used the MIP panel genotypes and quantified mutations occurring in the same isolates.[9] None of the following mutations were seen in K13 561H isolates:

- *Plasmodium falciparum* chloroquine resistance transporter (CRT) 326S and 356T
- *Plasmodium falciparum* multidrug resistance protein 2 (MDR2) 484I
- *Plasmodium falciparum* protein phosphatase (PPH) 1157L
- putative phosphoinositide-binding protein (PIB7) 1484F

However, ferredoxin (FD) 193Y polymorphisms were found in 13 K13 561H isolates in Kagera (**Table S4, p.9**).

Table S1. Antimalarial resistance polymorphisms for partner and chemoprevention agents reported (Adapted from [10–12])

Protein	Mutation/Haplotype	Drug	Impact
Chloroquine resistance transporter (CRT)	76T	Chloroquine and Amodiaquine	Heme detoxification in digestive vacuole
Multidrug resistance protein 1 (MDR1)	N86-184F-D1246 Haplotype	Lumefantrine	Potentially the inhibition of hemoglobin import and/or heme detoxification
Dihydropteroate synthase (DHPS)	437G, 540E, 581G	Sulfadoxine	Enzyme kinetics of folate biosynthesis in the parasite cytosol
Dihydrofolate reductase (DHFR)	51I, 59R, 108N, 164L	Pyrimethamine	Enzyme kinetics of folate biosynthesis in the parasite cytosol
Kelch 13 (K13)	WHO validated/candidate mutations[13]<br><br>[446I, 553L, 441L, 537I/D, 458Y, 561H, 449A, 538V, 476I, 574L, 469F/, 568G, 493H, 580Y, 481V, 622I, 539T, 515K, 675V, 543T, 527H]	Artemisinin derivatives	Alter ubiquitination patterns and help parasites to resist accumulation of polyubiquitinated proteins and decrease hemoglobin uptake at the cytostome, leading to decreased artemisinin activation in the food vacuole

Table S2. Participant characteristics.

Variable		Attempted Parasitemias (n= 7,782)	Successful Parasitemias (n-6,855)
Gender, n (%)	Female	3,600 (46.3%)	3,175 (46.3%)
	Male	3,149 (40.5%)	2,771 (40.4%)
	Unknown	1033 (13.3%)	909 (13.3%)
Age, years (SD)		15.8 (16.5)	15.7 (16.3)
Age Distribution, n (%)*	Adult	2,544 (32.7%)	2,247 (32.8%)
	School Children	1,683 (21.6%)	1533 (22.4%)
	Young Children	2,498 (32.1%)	2,147 (31.3%)
	Unknown	1,057 (13.6%)	928 (13.5%)
Fever in Last 48 hours, n (%)	Yes	6,257 (80.4%)	5496 (80.2%)
	No	435 (5.6%)	398 (5.8%)
	Unknown	1090 (14.0%)	961 (14.0%)
Malaria Test in Last 14 Days, n (%)	Yes	238 (3.1%)	193 (2.8%)
	No	6,490 (83.4%)	5,734 (83.6%)
	Unknown	1054 (13.5%)	928 (13.5%)
Malaria Treatment in Last 14 Days, n (%)	Yes	253 (3.3%)	203 (3.0%)
	No	6461 (83.0%)	5,711 (83.3%)
	Unknown	1068 (13.7%)	941 (13.7%)
RDT Result, n (%)	Positive	7,727 (99.3%)	6,830 (99.6%)
	Negative	55 (0.7%)	25 (0.4%)
Region, n (%)	Kigoma (Community)	166 (2.1%)	163 (2.4%)
	Ruvuma (Community)	202 (2.6%)	198 (2.9%)
	Tanga (Community)	265 (3.4%)	215 (3.1%)
	Dar Es Salaam (Clinic)	570 (7.3%)	477 (7.0%)
	Dodoma (Clinic)	594 (7.6%)	528 (7.7%)
	Kagera (Clinic)	988 (12.7%)	941 (13.7%)
	Kilimanjaro (Clinic)	583 (7.5%	449 (6.6%)
	Manyara (Clinic)	557 (7.2%)	489 (7.1%)
	Mara (Clinic)	918 (11.8%)	791 (11.5%)

	**Mtwara (Clinic)**	**882 (11.3%)**	**699 (10.2%)**
	**Njombe (Clinic)**	**573 (7.4%)**	**548 (8.0%)**
	**Songwe (Clinic)**	**620 (8.0%)**	**581 (8.5%)**
	**Tabora (Clinic)**	**864 (11.1%)**	**776 (11.3%)**

* Age Distributions: Young Children <5 years of age; School Children 5-16 years of age; Adults >16 years of age[1]

Table S3. Public genomes used in the analysis.

Southeast Asia Samples	Rwanda Samples
PD0068-C	ERX4235773
PD0079-C	ERX4235772
PD0126-C	ERX4235771
PD0481-C	ERX4235770
PD0495-C	ERX4235769
PD0562-C	ERX4235768
PD0565-C	ERX4235767
PD0799-C	ERX4235766
PD0800-C	ERX4235765
PD0819-C	ERX4235764
PD0822-C	ERX4235763
PD0823-C	ERX4235762
PD0824-C	ERX4235761
PD0826-C	ERX4235760
PD0828-C	ERX4235759
PD0832-C	ERX4235758
PD0876-C	ERX4235757
PD0877-C	ERX4235756
QC0184-C	ERX4235755
QC0185-C	ERX4235754
QC0250-C	ERX4235753
RCN03272	ERX4235752
RCN03319	ERX4235751
RCN03339	ERX4235750
RCN03348	ERX4235749
RCN03388	
RCN03502	
RCN03510	
RCN03535	
RCN09301	
RCN09302	
RCN09312	
RCN09313	
RCN09314	
RCN09316	
RCN09317	
RCN09319	

RCN09326	[redacted]
RCN09329	[redacted]
RCN09340	[redacted]
RCN09399	[redacted]
RCN09402	[redacted]
RCN09466	[redacted]
RCN09486	[redacted]
RCN09489	[redacted]
RCN09503	[redacted]
RCN09544	[redacted]
RCN09571	[redacted]
RCN09580	[redacted]
RCN09592	[redacted]
RCN09602	[redacted]
RCN09658	[redacted]
RCN09677	[redacted]
RCN09694	[redacted]
RCN09716	[redacted]
RCN09812	[redacted]
RCN09828	[redacted]
RCN09832	[redacted]
RCN09845	[redacted]
RCN09935	[redacted]
RCN09954	[redacted]
RCN09963	[redacted]
RCN09964	[redacted]
RCN09979	[redacted]
RCN09989	[redacted]
RCN10002	[redacted]
RCN10007	[redacted]
RCN10008	[redacted]
RCN10009	[redacted]
RCN10019	[redacted]
RCN12401	[redacted]
RCN12402	[redacted]
RCN12403	[redacted]
RCN12404	[redacted]

Table S4. Mutations in antimalarial resistance genes. Only mutations with a UMI coverage of 10, a UMI alternate count of 3, and presence in at least three samples are represented here. The first row for each mutation is a prevalence fraction showing the number of samples with alternate UMIs above the alternate UMI threshold (numerator), and the total number of samples with a coverage above the coverage UMI threshold (denominator). The second row is a percentage representation of the fraction from the first row. The third row is a 90% confidence interval for the percentages of the second row.

Mutation	Dar Es Salaam	Dodoma	Kagera	Kigoma	Kilimanjaro	Manyara	Mara	Mtwara	Njombe	Ruvuma	Songwe	Tabora	Tanga	Overall
PI4K-Asn957His	38/43	82/88	345/399	9/11	130/141	75/83	129/151	71/76	185/205	30/30	176/202	36/43	19/23	1325/1495
	88.4%	93.2%	86.5%	81.8%	92.2%	90.4%	85.4%	93.4%	90.2%	100.0%	87.1%	83.7%	82.6%	88.6%
	80.3%-96.4%	88.8%-97.6%	83.6%-89.3%	62.7%-100.0%	88.5%-95.9%	85.0%-95.7%	80.7%-90.2%	88.7%-98.1%	86.8%-93.7%	100.0%-100.0%	83.3%-91.0%	74.5%-93.0%	69.6%-95.6%	87.3%-90.0%
PI4K-Asn968Lys	0/43	0/88	1/399	0/11	0/141	0/83	0/151	0/76	1/205	0/30	0/202	1/43	0/23	3/1495
	0.0%	0.0%	0.3%	0.0%	0.0%	0.0%	0.0%	0.0%	0.5%	0.0%	0.0%	2.3%	0.0%	0.2%
	0.0%-0.0%	0.0%-0.0%	0.0%-0.7%	0.0%-0.0%	0.0%-0.0%	0.0%-0.0%	0.0%-0.0%	0.0%-0.0%	0.0%-1.3%	0.0%-0.0%	0.0%-0.0%	0.0%-6.1%	0.0%-0.0%	0.0%-0.4%
PI4K-Asp954Asn	0/43	1/88	0/399	0/11	0/141	0/83	0/151	0/76	1/205	0/30	1/202	0/43	0/23	3/1495
	0.0%	1.1%	0.0%	0.0%	0.0%	0.0%	0.0%	0.0%	0.5%	0.0%	0.5%	0.0%	0.0%	0.2%
	0.0%-0.0%	0.0%-3.0%	0.0%-0.0%	0.0%-0.0%	0.0%-0.0%	0.0%-0.0%	0.0%-0.0%	0.0%-0.0%	0.0%-1.3%	0.0%-0.0%	0.0%-1.3%	0.0%-0.0%	0.0%-0.0%	0.0%-0.4%
PI4K-Ile876Val	3/6	37/64	149/248	0/1	82/122	26/53	77/118	9/23	69/122	12/15	34/77	10/19	2/5	510/873
	50.0%	57.8%	60.1%	0.0%	67.2%	49.1%	65.3%	39.1%	56.6%	80.0%	44.2%	52.6%	40.0%	58.4%
	16.4%-83.6%	47.7%-68.0%	55.0%-65.2%	0.0%-0.0%	60.2%-74.2%	37.8%-60.4%	58.0%-72.5%	22.4%-55.9%	49.2%-63.9%	63.0%-97.0%	34.8%-53.5%	33.8%-71.5%	4.0%-76.0%	55.7%-61.2%
PI4K-Lys905Asn	0/6	0/64	0/248	0/1	2/122	1/53	0/118	1/23	4/122	0/15	0/77	0/19	0/5	8/873
	0.0%	0.0%	0.0%	0.0%	1.6%	1.9%	0.0%	4.3%	3.3%	0.0%	0.0%	0.0%	0.0%	0.9%
	0.0%-0.0%	0.0%-0.0%	0.0%-0.0%	0.0%-0.0%	0.0%-3.5%	0.0%-5.0%	0.0%-0.0%	0.0%-11.3%	0.6%-5.9%	0.0%-0.0%	0.0%-0.0%	0.0%-0.0%	0.0%-0.0%	0.4%-1.4%
PI4K-Lys927Thr	1/60	0/104	1/430	0/12	1/154	0/95	1/165	2/88	1/231	0/35	0/232	0/51	0/28	7/1685
	1.7%	0.0%	0.2%	0.0%	0.6%	0.0%	0.6%	2.3%	0.4%	0.0%	0.0%	0.0%	0.0%	0.4%
	0.0%-4.4%	0.0%-0.0%	0.0%-0.6%	0.0%-0.0%	0.0%-1.7%	0.0%-0.0%	0.0%-1.6%	0.0%-4.9%	0.0%-1.1%	0.0%-0.0%	0.0%-0.0%	0.0%-0.0%	0.0%-0.0%	0.2%-0.7%
PI4K-Ser936Asn	1/60	2/104	1/430	0/12	1/154	1/95	0/165	0/88	0/231	0/35	0/232	0/51	0/28	6/1685
	1.7%	1.9%	0.2%	0.0%	0.6%	1.1%	0.0%	0.0%	0.0%	0.0%	0.0%	0.0%	0.0%	0.4%
	0.0%-4.4%	0.0%-4.1%	0.0%-0.6%	0.0%-0.0%	0.0%-1.7%	0.0%-2.8%	0.0%-0.0%	0.0%-0.0%	0.0%-0.0%	0.0%-0.0%	0.0%-0.0%	0.0%-0.0%	0.0%-0.0%	0.1%-0.6%
PI4K-Tyr883His	2/6	38/64	139/248	0/1	76/122	28/53	68/118	10/23	70/122	10/15	39/77	8/19	2/5	490/873
	33.3%	59.4%	56.0%	0.0%	62.3%	52.8%	57.6%	43.5%	57.4%	66.7%	50.6%	42.1%	40.0%	56.1%
	1.7%-65.0%	49.3%-69.5%	50.9%-61.2%	0.0%-0.0%	55.1%-69.5%	41.6%-64.1%	50.1%-65.1%	26.5%-60.5%	50.0%-64.7%	46.6%-86.7%	41.3%-60.0%	23.5%-60.7%	4.0%-76.0%	53.4%-58.9%
PI4K-Val951Gly	0/43	0/88	4/399	0/11	0/141	0/83	0/151	0/76	0/205	0/30	0/202	0/43	0/23	4/1495
	0.0%	0.0%	1.0%	0.0%	0.0%	0.0%	0.0%	0.0%	0.0%	0.0%	0.0%	0.0%	0.0%	0.3%
	0.0%-0.0%	0.0%-0.0%	0.2%-1.8%	0.0%-0.0%	0.0%-0.0%	0.0%-0.0%	0.0%-0.0%	0.0%-0.0%	0.0%-0.0%	0.0%-0.0%	0.0%-0.0%	0.0%-0.0%	0.0%-0.0%	0.0%-0.5%
PI4K-Val972Met	0/43	2/88	3/399	0/11	0/141	0/83	1/151	1/76	1/205	0/30	1/202	2/43	0/23	11/1495
	0.0%	2.3%	0.8%	0.0%	0.0%	0.0%	0.7%	1.3%	0.5%	0.0%	0.5%	4.7%	0.0%	0.7%
	0.0%-0.0%	0.0%-4.9%	0.0%-1.5%	0.0%-0.0%	0.0%-0.0%	0.0%-0.0%	0.0%-1.7%	0.0%-3.5%	0.0%-1.3%	0.0%-0.0%	0.0%-1.3%	0.0%-9.9%	0.0%-0.0%	0.4%-1.1%
Pfap2mu-Ala236Thr	1/39	3/74	17/326	0/10	11/141	2/67	4/138	1/85	9/172	0/26	1/147	1/27	2/23	52/1275
	2.6%	4.1%	5.2%	0.0%	7.8%	3.0%	2.9%	1.2%	5.2%	0.0%	0.7%	3.7%	8.7%	4.1%
	0.0%-6.7%	0.3%-7.8%	3.2%-7.2%	0.0%-0.0%	4.1%-11.5%	0.0%-6.4%	0.5%-5.2%	0.0%-3.1%	2.4%-8.0%	0.0%-0.0%	0.0%-1.8%	0.0%-9.7%	0.0%-18.4%	3.2%-5.0%
Pfap2mu-Ala337Ser	1/67	0/96	1/403	0/8	1/137	0/88	1/141	0/90	0/199	0/27	1/208	0/50	0/16	5/1530
	1.5%	0.0%	0.2%	0.0%	0.7%	0.0%	0.7%	0.0%	0.0%	0.0%	0.5%	0.0%	0.0%	0.3%
	0.0%-3.9%	0.0%-0.0%	0.0%-0.7%	0.0%-0.0%	0.0%-1.9%	0.0%-0.0%	0.0%-1.9%	0.0%-0.0%	0.0%-0.0%	0.0%-0.0%	0.0%-1.3%	0.0%-0.0%	0.0%-0.0%	0.1%-0.6%

Pfap2mu-Ala337Thr	2/67	0/96	0/403	0/8	0/137	0/88	1/141	0/90	0/199	0/27	0/208	0/50	0/16	3/1530
	3.0%	0.0%	0.0%	0.0%	0.0%	0.0%	0.7%	0.0%	0.0%	0.0%	0.0%	0.0%	0.0%	0.2%
	0.0%-6.4%	0.0%-0.0%	0.0%-0.0%	0.0%-0.0%	0.0%-0.0%	0.0%-0.0%	0.0%-1.9%	0.0%-0.0%	0.0%-0.0%	0.0%-0.0%	0.0%-0.0%	0.0%-0.0%	0.0%-0.0%	0.0%-0.4%
Pfap2mu-Arg146Lys	0/51	1/72	10/344	0/4	1/113	4/63	3/113	1/73	0/152	0/17	2/162	2/24	0/11	24/1199
	0.0%	1.4%	2.9%	0.0%	0.9%	6.3%	2.7%	1.4%	0.0%	0.0%	1.2%	8.3%	0.0%	2.0%
	0.0%-0.0%	0.0%-3.7%	1.4%-4.4%	0.0%-0.0%	0.0%-2.3%	1.3%-11.4%	0.2%-5.1%	0.0%-3.6%	0.0%-0.0%	0.0%-0.0%	0.0%-2.7%	0.0%-17.6%	0.0%-0.0%	1.3%-2.7%
Pfap2mu-Asn200Tyr	3/31	2/68	2/298	1/4	1/128	0/58	3/131	2/76	1/132	0/20	0/122	0/21	0/14	15/1103
	9.7%	2.9%	0.7%	25.0%	0.8%	0.0%	2.3%	2.6%	0.8%	0.0%	0.0%	0.0%	0.0%	1.4%
	0.9%-18.4%	0.0%-6.3%	0.0%-1.4%	0.0%-60.6%	0.0%-2.1%	0.0%-0.0%	0.1%-4.4%	0.0%-5.7%	0.0%-2.0%	0.0%-0.0%	0.0%-0.0%	0.0%-0.0%	0.0%-0.0%	0.8%-1.9%
Pfap2mu-Asn233Lys	1/39	1/74	2/326	0/10	2/141	0/67	0/138	0/85	0/172	0/26	3/146	0/27	0/23	9/1274
	2.6%	1.4%	0.6%	0.0%	1.4%	0.0%	0.0%	0.0%	0.0%	0.0%	2.1%	0.0%	0.0%	0.7%
	0.0%-6.7%	0.0%-3.6%	0.0%-1.3%	0.0%-0.0%	0.0%-3.1%	0.0%-0.0%	0.0%-0.0%	0.0%-0.0%	0.0%-0.0%	0.0%-0.0%	0.1%-4.0%	0.0%-0.0%	0.0%-0.0%	0.3%-1.1%
Pfap2mu-Gly315Ser	0/41	0/75	1/348	0/3	1/117	0/65	2/119	1/79	0/151	0/20	0/153	2/34	0/7	7/1212
	0.0%	0.0%	0.3%	0.0%	0.9%	0.0%	1.7%	1.3%	0.0%	0.0%	0.0%	5.9%	0.0%	0.6%
	0.0%-0.0%	0.0%-0.0%	0.0%-0.8%	0.0%-0.0%	0.0%-2.3%	0.0%-0.0%	0.0%-3.6%	0.0%-3.3%	0.0%-0.0%	0.0%-0.0%	0.0%-0.0%	0.0%-12.5%	0.0%-0.0%	0.2%-0.9%
Pfap2mu-Lys199Thr	8/31	12/68	29/298	0/4	18/128	6/58	14/131	3/76	29/132	2/20	20/122	2/21	4/14	147/1103
	25.8%	17.6%	9.7%	0.0%	14.1%	10.3%	10.7%	3.9%	22.0%	10.0%	16.4%	9.5%	28.6%	13.3%
	12.9%-38.7%	10.0%-25.3%	6.9%-12.6%	0.0%-0.0%	9.0%-19.1%	3.8%-16.9%	6.2%-15.1%	0.3%-7.6%	16.0%-27.9%	0.0%-21.0%	10.9%-21.9%	0.0%-20.1%	8.7%-48.4%	11.6%-15.0%
Pfap2mu-Phe437Leu	0/1	2/70	5/271	0/2	4/134	2/54	1/123	0/30	0/128	0/25	2/104	0/20	2/15	18/977
	0.0%	2.9%	1.8%	0.0%	3.0%	3.7%	0.8%	0.0%	0.0%	0.0%	1.9%	0.0%	13.3%	1.8%
	0.0%-0.0%	0.0%-6.1%	0.5%-3.2%	0.0%-0.0%	0.6%-5.4%	0.0%-7.9%	0.0%-2.1%	0.0%-0.0%	0.0%-0.0%	0.0%-0.0%	0.0%-4.1%	0.0%-0.0%	0.0%-27.8%	1.1%-2.6%
Pfap2mu-Ser160Asn	6/51	6/72	67/344	0/4	22/113	7/63	16/113	8/72	32/152	1/17	18/162	5/24	3/11	191/1198
	11.8%	8.3%	19.5%	0.0%	19.5%	11.1%	14.2%	11.1%	21.1%	5.9%	11.1%	20.8%	27.3%	15.9%
	4.3%-19.2%	3.0%-13.7%	16.0%-23.0%	0.0%-0.0%	13.3%-25.6%	4.6%-17.6%	8.8%-19.6%	5.0%-17.2%	15.6%-26.5%	0.0%-15.3%	7.0%-15.2%	7.2%-34.5%	5.2%-49.4%	14.2%-17.7%
Pfap2mu-Ser160Thr	0/51	0/72	3/344	0/4	0/113	0/63	9/113	2/72	0/152	0/17	3/162	1/24	0/11	18/1198
	0.0%	0.0%	0.9%	0.0%	0.0%	0.0%	8.0%	2.8%	0.0%	0.0%	1.9%	4.2%	0.0%	1.5%
	0.0%-0.0%	0.0%-0.0%	0.0%-1.7%	0.0%-0.0%	0.0%-0.0%	0.0%-0.0%	3.8%-12.2%	0.0%-6.0%	0.0%-0.0%	0.0%-0.0%	0.1%-3.6%	0.0%-10.9%	0.0%-0.0%	0.9%-2.1%
Pfap2mu-Ser326Cys	1/41	0/75	0/348	0/3	1/117	0/65	0/119	0/79	0/151	1/20	0/153	0/34	0/7	3/1212
	2.4%	0.0%	0.0%	0.0%	0.9%	0.0%	0.0%	0.0%	0.0%	5.0%	0.0%	0.0%	0.0%	0.2%
	0.0%-6.4%	0.0%-0.0%	0.0%-0.0%	0.0%-0.0%	0.0%-2.3%	0.0%-0.0%	0.0%-0.0%	0.0%-0.0%	0.0%-0.0%	0.0%-13.0%	0.0%-0.0%	0.0%-0.0%	0.0%-0.0%	0.0%-0.5%
Pfap2mu-Ser476Ala	0/40	0/77	1/390	0/9	1/136	0/61	5/146	0/81	1/185	0/29	2/193	0/39	0/20	10/1406
	0.0%	0.0%	0.3%	0.0%	0.7%	0.0%	3.4%	0.0%	0.5%	0.0%	1.0%	0.0%	0.0%	0.7%
	0.0%-0.0%	0.0%-0.0%	0.0%-0.7%	0.0%-0.0%	0.0%-1.9%	0.0%-0.0%	0.9%-5.9%	0.0%-0.0%	0.0%-1.4%	0.0%-0.0%	0.0%-2.2%	0.0%-0.0%	0.0%-0.0%	0.3%-1.1%
Pfap2mu-Ser508Cys	0/33	0/66	3/346	0/6	0/114	0/56	0/127	0/71	1/165	0/21	0/163	0/34	0/10	4/1212
	0.0%	0.0%	0.9%	0.0%	0.0%	0.0%	0.0%	0.0%	0.6%	0.0%	0.0%	0.0%	0.0%	0.3%
	0.0%-0.0%	0.0%-0.0%	0.0%-1.7%	0.0%-0.0%	0.0%-0.0%	0.0%-0.0%	0.0%-0.0%	0.0%-0.0%	0.0%-1.6%	0.0%-0.0%	0.0%-0.0%	0.0%-0.0%	0.0%-0.0%	0.1%-0.6%
Pfubp1-Ala1084Thr	0/30	0/72	1/311	0/4	1/128	0/57	0/127	0/76	0/137	1/22	0/117	0/22	0/13	3/1116
	0.0%	0.0%	0.3%	0.0%	0.8%	0.0%	0.0%	0.0%	0.0%	4.5%	0.0%	0.0%	0.0%	0.3%
	0.0%-0.0%	0.0%-0.0%	0.0%-0.8%	0.0%-0.0%	0.0%-2.1%	0.0%-0.0%	0.0%-0.0%	0.0%-0.0%	0.0%-0.0%	0.0%-11.9%	0.0%-0.0%	0.0%-0.0%	0.0%-0.0%	0.0%-0.5%
Pfubp1-Ala1883Thr	2/251	0/278	3/629	0/39	0/234	0/291	0/350	0/302	0/343	0/63	0/395	1/407	0/68	6/3650
	0.8%	0.0%	0.5%	0.0%	0.0%	0.0%	0.0%	0.0%	0.0%	0.0%	0.0%	0.2%	0.0%	0.2%
	0.0%-1.7%	0.0%-0.0%	0.0%-0.9%	0.0%-0.0%	0.0%-0.0%	0.0%-0.0%	0.0%-0.0%	0.0%-0.0%	0.0%-0.0%	0.0%-0.0%	0.0%-0.0%	0.0%-0.6%	0.0%-0.0%	0.1%-0.3%
Pfubp1-Arg1133Ser	14/25	34/61	218/334	no coverage	65/105	25/49	76/111	40/60	102/149	15/20	94/151	11/18	1/2	695/1085
	56.0%	55.7%	65.3%	no coverage	61.9%	51.0%	68.5%	66.7%	68.5%	75.0%	62.3%	61.1%	50.0%	64.1%

	39.7%-72.3%	45.3%-66.2%	61.0%-69.6%	no coverage	54.1%-69.7%	39.3%-62.8%	61.2%-75.7%	56.7%-76.7%	62.2%-74.7%	59.1%-90.9%	55.8%-68.7%	42.2%-80.0%	0.0%-100.0%	61.7%-66.5%
Pfubp1-Arg1205Cys	0/130	0/144	1/515	0/11	0/149	0/162	3/162	0/125	0/252	0/30	0/287	0/136	0/16	4/2119
	0.0%	0.0%	0.2%	0.0%	0.0%	0.0%	1.9%	0.0%	0.0%	0.0%	0.0%	0.0%	0.0%	0.2%
	0.0%-0.0%	0.0%-0.0%	0.0%-0.5%	0.0%-0.0%	0.0%-0.0%	0.0%-0.0%	0.1%-3.6%	0.0%-0.0%	0.0%-0.0%	0.0%-0.0%	0.0%-0.0%	0.0%-0.0%	0.0%-0.0%	0.0%-0.3%
Pfubp1-Arg1205His	4/161	0/171	0/562	0/17	0/183	0/185	0/209	0/149	0/299	0/41	0/343	0/174	0/38	4/2532
	2.5%	0.0%	0.0%	0.0%	0.0%	0.0%	0.0%	0.0%	0.0%	0.0%	0.0%	0.0%	0.0%	0.2%
	0.5%-4.5%	0.0%-0.0%	0.0%-0.0%	0.0%-0.0%	0.0%-0.0%	0.0%-0.0%	0.0%-0.0%	0.0%-0.0%	0.0%-0.0%	0.0%-0.0%	0.0%-0.0%	0.0%-0.0%	0.0%-0.0%	0.0%-0.3%
Pfubp1-Arg2226Lys	0/1	0/62	1/221	no coverage	2/111	0/47	3/104	no coverage	0/87	0/17	0/65	0/17	0/1	6/733
	0.0%	0.0%	0.5%	no coverage	1.8%	0.0%	2.9%	no coverage	0.0%	0.0%	0.0%	0.0%	0.0%	0.8%
	0.0%-0.0%	0.0%-0.0%	0.0%-1.2%	no coverage	0.0%-3.9%	0.0%-0.0%	0.2%-5.6%	no coverage	0.0%-0.0%	0.0%-0.0%	0.0%-0.0%	0.0%-0.0%	0.0%-0.0%	0.3%-1.4%
Pfubp1-Arg2238Lys	83/85	111/114	479/488	11/12	154/157	108/109	154/164	103/106	235/238	38/38	225/233	57/58	31/31	1789/1833
	97.6%	97.4%	98.2%	91.7%	98.1%	99.1%	93.9%	97.2%	98.7%	100.0%	96.6%	98.3%	100.0%	97.6%
	94.9%-100.0%	94.9%-99.8%	97.2%-99.2%	78.5%-100.0%	96.3%-99.9%	97.6%-100.0%	90.8%-97.0%	94.5%-99.8%	97.6%-99.9%	100.0%-100.0%	94.6%-98.5%	95.5%-100.0%	00.0%-100.0%	97.0%-98.2%
Pfubp1-Arg2385Thr	0/19	0/77	3/331	0/7	0/146	0/68	0/137	0/28	0/196	0/37	0/153	0/22	0/13	3/1234
	0.0%	0.0%	0.9%	0.0%	0.0%	0.0%	0.0%	0.0%	0.0%	0.0%	0.0%	0.0%	0.0%	0.2%
	0.0%-0.0%	0.0%-0.0%	0.0%-1.8%	0.0%-0.0%	0.0%-0.0%	0.0%-0.0%	0.0%-0.0%	0.0%-0.0%	0.0%-0.0%	0.0%-0.0%	0.0%-0.0%	0.0%-0.0%	0.0%-0.0%	0.0%-0.5%
Pfubp1-Arg2445Lys	no coverage	3/45	0/179	no coverage	0/69	0/27	0/101	0/1	0/55	0/8	0/38	0/8	0/1	3/532
	no coverage	6.7%	0.0%	no coverage	0.0%	0.0%	0.0%	0.0%	0.0%	0.0%	0.0%	0.0%	0.0%	0.6%
	no coverage	0.6%-12.8%	0.0%-0.0%	no coverage	0.0%-0.0%	0.0%-0.0%	0.0%-0.0%	0.0%-0.0%	0.0%-0.0%	0.0%-0.0%	0.0%-0.0%	0.0%-0.0%	0.0%-0.0%	0.0%-1.1%
Pfubp1-Asn101Ile	0/29	0/51	0/295	no coverage	0/82	0/35	5/111	0/60	0/125	0/22	0/125	0/14	0/2	5/951
	0.0%	0.0%	0.0%	no coverage	0.0%	0.0%	4.5%	0.0%	0.0%	0.0%	0.0%	0.0%	0.0%	0.5%
	0.0%-0.0%	0.0%-0.0%	0.0%-0.0%	no coverage	0.0%-0.0%	0.0%-0.0%	1.3%-7.7%	0.0%-0.0%	0.0%-0.0%	0.0%-0.0%	0.0%-0.0%	0.0%-0.0%	0.0%-0.0%	0.1%-0.9%
Pfubp1-Asn157Lys	50/192	51/194	165/560	6/18	49/181	72/207	71/229	50/185	76/294	11/41	79/352	55/211	11/40	746/2704
	26.0%	26.3%	29.5%	33.3%	27.1%	34.8%	31.0%	27.0%	25.9%	26.8%	22.4%	26.1%	27.5%	27.6%
	20.8%-31.3%	21.1%-31.5%	26.3%-32.6%	15.1%-51.6%	21.6%-32.5%	29.3%-40.2%	26.0%-36.0%	21.7%-32.4%	21.7%-30.1%	15.4%-38.2%	18.8%-26.1%	21.1%-31.0%	15.9%-39.1%	26.2%-29.0%
Pfubp1-Asn1704Lys	0/103	1/110	4/455	0/5	0/139	1/113	1/149	0/104	1/204	0/29	0/239	0/97	0/15	8/1762
	0.0%	0.9%	0.9%	0.0%	0.0%	0.9%	0.7%	0.0%	0.5%	0.0%	0.0%	0.0%	0.0%	0.5%
	0.0%-0.0%	0.0%-2.4%	0.2%-1.6%	0.0%-0.0%	0.0%-0.0%	0.0%-2.3%	0.0%-1.8%	0.0%-0.0%	0.0%-1.3%	0.0%-0.0%	0.0%-0.0%	0.0%-0.0%	0.0%-0.0%	0.2%-0.7%
Pfubp1-Asn1706Lys	3/103	0/110	0/455	0/5	1/139	0/113	1/149	0/104	0/204	0/29	0/239	0/97	0/15	5/1762
	2.9%	0.0%	0.0%	0.0%	0.7%	0.0%	0.7%	0.0%	0.0%	0.0%	0.0%	0.0%	0.0%	0.3%
	0.2%-5.6%	0.0%-0.0%	0.0%-0.0%	0.0%-0.0%	0.0%-1.9%	0.0%-0.0%	0.0%-1.8%	0.0%-0.0%	0.0%-0.0%	0.0%-0.0%	0.0%-0.0%	0.0%-0.0%	0.0%-0.0%	0.1%-0.5%
Pfubp1-Asn1710Ser	3/103	3/110	11/455	1/5	1/139	3/113	7/149	3/104	2/204	0/29	3/239	9/97	4/15	50/1762
	2.9%	2.7%	2.4%	20.0%	0.7%	2.7%	4.7%	2.9%	1.0%	0.0%	1.3%	9.3%	26.7%	2.8%
	0.2%-5.6%	0.2%-5.3%	1.2%-3.6%	0.0%-49.4%	0.0%-1.9%	0.2%-5.1%	1.8%-7.5%	0.2%-5.6%	0.0%-2.1%	0.0%-0.0%	0.1%-2.4%	4.4%-14.1%	7.9%-45.4%	2.2%-3.5%
Pfubp1-Asn1712Lys	0/103	0/110	0/455	0/5	0/139	0/113	3/149	0/104	0/204	0/29	0/239	0/97	0/15	3/1762
	0.0%	0.0%	0.0%	0.0%	0.0%	0.0%	2.0%	0.0%	0.0%	0.0%	0.0%	0.0%	0.0%	0.2%
	0.0%-0.0%	0.0%-0.0%	0.0%-0.0%	0.0%-0.0%	0.0%-0.0%	0.0%-0.0%	0.1%-3.9%	0.0%-0.0%	0.0%-0.0%	0.0%-0.0%	0.0%-0.0%	0.0%-0.0%	0.0%-0.0%	0.0%-0.3%
Pfubp1-Asn189Lys	0/195	0/196	2/576	0/22	0/194	1/216	3/251	0/212	0/295	0/41	1/335	0/230	0/49	7/2812
	0.0%	0.0%	0.3%	0.0%	0.0%	0.5%	1.2%	0.0%	0.0%	0.0%	0.3%	0.0%	0.0%	0.2%
	0.0%-0.0%	0.0%-0.0%	0.0%-0.8%	0.0%-0.0%	0.0%-0.0%	0.0%-1.2%	0.1%-2.3%	0.0%-0.0%	0.0%-0.0%	0.0%-0.0%	0.0%-0.8%	0.0%-0.0%	0.0%-0.0%	0.1%-0.4%
Pfubp1-Asn1901Tyr	0/243	0/273	0/622	0/34	0/227	3/277	0/337	2/291	0/330	0/59	0/390	0/388	0/63	5/3534
	0.0%	0.0%	0.0%	0.0%	0.0%	1.1%	0.0%	0.7%	0.0%	0.0%	0.0%	0.0%	0.0%	0.1%
	0.0%-0.0%	0.0%-0.0%	0.0%-0.0%	0.0%-0.0%	0.0%-0.0%	0.1%-2.1%	0.0%-0.0%	0.0%-1.5%	0.0%-0.0%	0.0%-0.0%	0.0%-0.0%	0.0%-0.0%	0.0%-0.0%	0.0%-0.2%
Pfubp1-Asn1935Lys	1/1	1/2	11/29	no coverage	0/1	1/2	8/23	9/13	1/2	0/1	5/6	1/1	1/1	39/82

	100.0%	50.0%	37.9%	no coverage	0.0%	50.0%	34.8%	69.2%	50.0%	0.0%	83.3%	100.0%	100.0%	47.6%
	100.0%-100.0%	0.0%-100.0%	23.1%-52.8%	no coverage	0.0%-0.0%	0.0%-100.0%	18.4%-51.1%	48.2%-90.3%	0.0%-100.0%	0.0%-0.0%	58.3%-100.0%	100.0%-100.0%	00.0%-100.0%	38.5%-56.6%
Pfubp1-Asn1942Lys	1/4	2/29	27/111	no coverage	2/20	2/12	8/26	3/7	8/47	2/6	8/30	0/5	1/1	64/298
	25.0%	6.9%	24.3%	no coverage	10.0%	16.7%	30.8%	42.9%	17.0%	33.3%	26.7%	0.0%	100.0%	21.5%
	0.0%-60.6%	0.0%-14.6%	17.6%-31.0%	no coverage	0.0%-21.0%	0.0%-34.4%	15.9%-45.7%	12.1%-73.6%	8.0%-26.0%	1.7%-65.0%	13.4%-39.9%	0.0%-0.0%	00.0%-100.0%	17.6%-25.4%
Pfubp1-Asn1944Ser	0/4	0/29	2/111	no coverage	0/20	0/12	0/26	0/7	0/47	0/6	1/30	0/5	0/1	3/298
	0.0%	0.0%	1.8%	no coverage	0.0%	0.0%	0.0%	0.0%	0.0%	0.0%	3.3%	0.0%	0.0%	1.0%
	0.0%-0.0%	0.0%-0.0%	0.0%-3.9%	no coverage	0.0%-0.0%	0.0%-0.0%	0.0%-0.0%	0.0%-0.0%	0.0%-0.0%	0.0%-0.0%	0.0%-8.7%	0.0%-0.0%	0.0%-0.0%	0.1%-2.0%
Pfubp1-Asn1963Lys	0/87	1/112	5/472	0/8	0/87	1/102	1/144	3/92	5/212	0/29	1/241	1/86	0/11	18/1683
	0.0%	0.9%	1.1%	0.0%	0.0%	1.0%	0.7%	3.3%	2.4%	0.0%	0.4%	1.2%	0.0%	1.1%
	0.0%-0.0%	0.0%-2.4%	0.3%-1.8%	0.0%-0.0%	0.0%-0.0%	0.0%-2.6%	0.0%-1.8%	0.2%-6.3%	0.6%-4.1%	0.0%-0.0%	0.0%-1.1%	0.0%-3.1%	0.0%-0.0%	0.7%-1.5%
Pfubp1-Asn2524Ile	0/68	0/97	2/430	0/9	1/134	1/94	0/135	0/85	0/218	0/26	1/225	0/57	0/17	5/1595
	0.0%	0.0%	0.5%	0.0%	0.7%	1.1%	0.0%	0.0%	0.0%	0.0%	0.4%	0.0%	0.0%	0.3%
	0.0%-0.0%	0.0%-0.0%	0.0%-1.0%	0.0%-0.0%	0.0%-2.0%	0.0%-2.8%	0.0%-0.0%	0.0%-0.0%	0.0%-0.0%	0.0%-0.0%	0.0%-1.2%	0.0%-0.0%	0.0%-0.0%	0.1%-0.5%
Pfubp1-Asn274Lys	0/19	0/58	0/254	0/3	0/109	0/48	5/117	0/22	5/137	1/31	1/100	0/12	0/5	12/915
	0.0%	0.0%	0.0%	0.0%	0.0%	0.0%	4.3%	0.0%	3.6%	3.2%	1.0%	0.0%	0.0%	1.3%
	0.0%-0.0%	0.0%-0.0%	0.0%-0.0%	0.0%-0.0%	0.0%-0.0%	0.0%-0.0%	1.2%-7.3%	0.0%-0.0%	1.0%-6.3%	0.0%-8.4%	0.0%-2.6%	0.0%-0.0%	0.0%-0.0%	0.7%-1.9%
Pfubp1-Asn2813Lys	4/167	1/165	29/558	2/19	4/179	6/183	17/215	7/169	15/271	0/49	21/285	6/181	2/38	114/2479
	2.4%	0.6%	5.2%	10.5%	2.2%	3.3%	7.9%	4.1%	5.5%	0.0%	7.4%	3.3%	5.3%	4.6%
	0.4%-4.3%	0.0%-1.6%	3.7%-6.7%	0.0%-22.1%	0.4%-4.1%	1.1%-5.4%	4.9%-10.9%	1.6%-6.7%	3.3%-7.8%	0.0%-0.0%	4.8%-9.9%	1.1%-5.5%	0.0%-11.2%	3.9%-5.3%
Pfubp1-Asn2838Ser	0/166	0/164	4/557	0/18	0/178	0/182	1/214	0/169	0/270	0/48	0/284	0/181	0/37	5/2468
	0.0%	0.0%	0.7%	0.0%	0.0%	0.0%	0.5%	0.0%	0.0%	0.0%	0.0%	0.0%	0.0%	0.2%
	0.0%-0.0%	0.0%-0.0%	0.1%-1.3%	0.0%-0.0%	0.0%-0.0%	0.0%-0.0%	0.0%-1.2%	0.0%-0.0%	0.0%-0.0%	0.0%-0.0%	0.0%-0.0%	0.0%-0.0%	0.0%-0.0%	0.1%-0.4%
Pfubp1-Asn2969Lys	0/91	0/111	3/480	0/7	0/135	0/102	0/151	0/102	0/227	0/26	0/232	0/67	0/10	3/1741
	0.0%	0.0%	0.6%	0.0%	0.0%	0.0%	0.0%	0.0%	0.0%	0.0%	0.0%	0.0%	0.0%	0.2%
	0.0%-0.0%	0.0%-0.0%	0.0%-1.2%	0.0%-0.0%	0.0%-0.0%	0.0%-0.0%	0.0%-0.0%	0.0%-0.0%	0.0%-0.0%	0.0%-0.0%	0.0%-0.0%	0.0%-0.0%	0.0%-0.0%	0.0%-0.3%
Pfubp1-Asn3054Lys	0/132	0/173	0/552	0/14	0/169	0/160	2/196	0/136	0/275	0/39	0/331	1/147	0/25	3/2349
	0.0%	0.0%	0.0%	0.0%	0.0%	0.0%	1.0%	0.0%	0.0%	0.0%	0.0%	0.7%	0.0%	0.1%
	0.0%-0.0%	0.0%-0.0%	0.0%-0.0%	0.0%-0.0%	0.0%-0.0%	0.0%-0.0%	0.0%-2.2%	0.0%-0.0%	0.0%-0.0%	0.0%-0.0%	0.0%-0.0%	0.0%-1.8%	0.0%-0.0%	0.0%-0.2%
Pfubp1-Asn3133Ser	0/121	0/139	0/472	0/3	1/138	1/143	0/166	0/135	1/246	0/24	0/219	1/160	0/14	4/1980
	0.0%	0.0%	0.0%	0.0%	0.7%	0.7%	0.0%	0.0%	0.4%	0.0%	0.0%	0.6%	0.0%	0.2%
	0.0%-0.0%	0.0%-0.0%	0.0%-0.0%	0.0%-0.0%	0.0%-1.9%	0.0%-1.8%	0.0%-0.0%	0.0%-0.0%	0.0%-1.1%	0.0%-0.0%	0.0%-0.0%	0.0%-1.6%	0.0%-0.0%	0.0%-0.4%
Pfubp1-Asn640Lys	2/34	2/72	7/345	0/12	3/134	2/60	10/140	2/70	0/193	0/29	4/177	1/28	0/18	33/1312
	5.9%	2.8%	2.0%	0.0%	2.2%	3.3%	7.1%	2.9%	0.0%	0.0%	2.3%	3.6%	0.0%	2.5%
	0.0%-12.5%	0.0%-6.0%	0.8%-3.3%	0.0%-0.0%	0.1%-4.3%	0.0%-7.1%	3.6%-10.7%	0.0%-6.1%	0.0%-0.0%	0.0%-0.0%	0.4%-4.1%	0.0%-9.3%	0.0%-0.0%	1.8%-3.2%
Pfubp1-Asn749Lys	0/159	0/162	0/480	0/18	0/153	0/161	0/213	3/186	0/250	0/43	0/285	0/220	0/25	3/2355
	0.0%	0.0%	0.0%	0.0%	0.0%	0.0%	0.0%	1.6%	0.0%	0.0%	0.0%	0.0%	0.0%	0.1%
	0.0%-0.0%	0.0%-0.0%	0.0%-0.0%	0.0%-0.0%	0.0%-0.0%	0.0%-0.0%	0.0%-0.0%	0.1%-3.1%	0.0%-0.0%	0.0%-0.0%	0.0%-0.0%	0.0%-0.0%	0.0%-0.0%	0.0%-0.2%
Pfubp1-Asn784Lys	6/112	8/95	7/346	0/5	5/83	11/92	4/133	5/125	19/179	0/14	2/204	6/136	0/4	73/1528
	5.4%	8.4%	2.0%	0.0%	6.0%	12.0%	3.0%	4.0%	10.6%	0.0%	1.0%	4.4%	0.0%	4.8%
	1.9%-8.9%	3.7%-13.1%	0.8%-3.3%	0.0%-0.0%	1.7%-10.3%	6.4%-17.5%	0.6%-5.4%	1.1%-6.9%	6.8%-14.4%	0.0%-0.0%	0.0%-2.1%	1.5%-7.3%	0.0%-0.0%	3.9%-5.7%
Pfubp1-Asn789Asp	0/112	0/95	13/346	0/5	0/83	0/92	1/133	1/125	1/179	0/14	2/204	3/136	0/4	21/1528
	0.0%	0.0%	3.8%	0.0%	0.0%	0.0%	0.8%	0.8%	0.6%	0.0%	1.0%	2.2%	0.0%	1.4%
	0.0%-0.0%	0.0%-0.0%	2.1%-5.4%	0.0%-0.0%	0.0%-0.0%	0.0%-0.0%	0.0%-2.0%	0.0%-2.1%	0.0%-1.5%	0.0%-0.0%	0.0%-2.1%	0.1%-4.3%	0.0%-0.0%	0.9%-1.9%

Pfubp1-Asn798Asp	6/112	8/95	19/346	0/5	5/83	11/92	5/133	6/125	20/179	0/14	3/204	9/136	0/4	92/1528
	5.4%	8.4%	5.5%	0.0%	6.0%	12.0%	3.8%	4.8%	11.2%	0.0%	1.5%	6.6%	0.0%	6.0%
	1.9%-8.9%	3.7%-13.1%	3.5%-7.5%	0.0%-0.0%	1.7%-10.3%	6.4%-17.5%	1.0%-6.5%	1.7%-7.9%	7.3%-15.0%	0.0%-0.0%	0.1%-2.9%	3.1%-10.1%	0.0%-0.0%	5.0%-7.0%
Pfubp1-Asn917Lys	0/15	0/65	0/219	0/2	0/79	1/54	2/103	0/20	0/98	1/21	2/76	0/17	0/4	6/773
	0.0%	0.0%	0.0%	0.0%	0.0%	1.9%	1.9%	0.0%	0.0%	4.8%	2.6%	0.0%	0.0%	0.8%
	0.0%-0.0%	0.0%-0.0%	0.0%-0.0%	0.0%-0.0%	0.0%-0.0%	0.0%-4.9%	0.0%-4.2%	0.0%-0.0%	0.0%-0.0%	0.0%-12.4%	0.0%-5.7%	0.0%-0.0%	0.0%-0.0%	0.3%-1.3%
Pfubp1-Asn920Asp	0/14	1/71	4/221	0/2	0/92	1/60	0/104	1/19	0/100	0/21	1/76	0/17	0/4	8/801
	0.0%	1.4%	1.8%	0.0%	0.0%	1.7%	0.0%	5.3%	0.0%	0.0%	1.3%	0.0%	0.0%	1.0%
	0.0%-0.0%	0.0%-3.7%	0.3%-3.3%	0.0%-0.0%	0.0%-0.0%	0.0%-4.4%	0.0%-0.0%	0.0%-13.7%	0.0%-0.0%	0.0%-0.0%	0.0%-3.5%	0.0%-0.0%	0.0%-0.0%	0.4%-1.6%
Pfubp1-Asn943Asp	no coverage	no coverage	3/43	no coverage	0/1	no coverage	1/30	no coverage	0/16	0/2	no coverage	no coverage	no coverage	4/92
	no coverage	no coverage	7.0%	no coverage	0.0%	no coverage	3.3%	no coverage	0.0%	0.0%	no coverage	no coverage	no coverage	4.3%
	no coverage	no coverage	0.6%-13.4%	no coverage	0.0%-0.0%	no coverage	0.0%-8.7%	no coverage	0.0%-0.0%	0.0%-0.0%	no coverage	no coverage	no coverage	0.9%-7.8%
Pfubp1-Asn964Lys	no coverage	0/16	2/119	no coverage	0/28	0/5	1/73	no coverage	2/26	0/3	0/5	0/1	no coverage	5/276
	no coverage	0.0%	1.7%	no coverage	0.0%	0.0%	1.4%	no coverage	7.7%	0.0%	0.0%	0.0%	no coverage	1.8%
	no coverage	0.0%-0.0%	0.0%-3.6%	no coverage	0.0%-0.0%	0.0%-0.0%	0.0%-3.6%	no coverage	0.0%-16.3%	0.0%-0.0%	0.0%-0.0%	0.0%-0.0%	no coverage	0.5%-3.1%
Pfubp1-Asn965Lys	no coverage	0/16	2/119	no coverage	0/28	0/5	1/73	no coverage	2/26	0/3	0/5	0/1	no coverage	5/276
	no coverage	0.0%	1.7%	no coverage	0.0%	0.0%	1.4%	no coverage	7.7%	0.0%	0.0%	0.0%	no coverage	1.8%
	no coverage	0.0%-0.0%	0.0%-3.6%	no coverage	0.0%-0.0%	0.0%-0.0%	0.0%-3.6%	no coverage	0.0%-16.3%	0.0%-0.0%	0.0%-0.0%	0.0%-0.0%	no coverage	0.5%-3.1%
Pfubp1-Asn966Lys	no coverage	0/16	2/119	no coverage	0/28	0/5	1/73	no coverage	1/26	0/3	0/5	0/1	no coverage	4/276
	no coverage	0.0%	1.7%	no coverage	0.0%	0.0%	1.4%	no coverage	3.8%	0.0%	0.0%	0.0%	no coverage	1.4%
	no coverage	0.0%-0.0%	0.0%-3.6%	no coverage	0.0%-0.0%	0.0%-0.0%	0.0%-3.6%	no coverage	0.0%-10.0%	0.0%-0.0%	0.0%-0.0%	0.0%-0.0%	no coverage	0.3%-2.6%
Pfubp1-Asp146Tyr	0/192	0/194	1/560	0/18	0/181	0/207	0/229	1/185	0/294	0/41	1/352	0/211	0/40	3/2704
	0.0%	0.0%	0.2%	0.0%	0.0%	0.0%	0.0%	0.5%	0.0%	0.0%	0.3%	0.0%	0.0%	0.1%
	0.0%-0.0%	0.0%-0.0%	0.0%-0.5%	0.0%-0.0%	0.0%-0.0%	0.0%-0.0%	0.0%-0.0%	0.0%-1.4%	0.0%-0.0%	0.0%-0.0%	0.0%-0.8%	0.0%-0.0%	0.0%-0.0%	0.0%-0.2%
Pfubp1-Asp158Asn	0/192	4/194	18/560	0/18	4/181	2/207	3/229	1/185	0/294	0/41	1/352	6/211	0/40	39/2704
	0.0%	2.1%	3.2%	0.0%	2.2%	1.0%	1.3%	0.5%	0.0%	0.0%	0.3%	2.8%	0.0%	1.4%
	0.0%-0.0%	0.4%-3.7%	2.0%-4.4%	0.0%-0.0%	0.4%-4.0%	0.0%-2.1%	0.1%-2.5%	0.0%-1.4%	0.0%-0.0%	0.0%-0.0%	0.0%-0.8%	1.0%-4.7%	0.0%-0.0%	1.1%-1.8%
Pfubp1-Asp180Tyr	0/169	0/173	0/542	0/17	0/171	0/186	2/208	0/170	3/258	0/32	0/282	0/178	0/34	5/2420
	0.0%	0.0%	0.0%	0.0%	0.0%	0.0%	1.0%	0.0%	1.2%	0.0%	0.0%	0.0%	0.0%	0.2%
	0.0%-0.0%	0.0%-0.0%	0.0%-0.0%	0.0%-0.0%	0.0%-0.0%	0.0%-0.0%	0.0%-2.1%	0.0%-0.0%	0.1%-2.3%	0.0%-0.0%	0.0%-0.0%	0.0%-0.0%	0.0%-0.0%	0.1%-0.4%
Pfubp1-Asp1878Glu	1/251	0/278	26/629	1/39	2/234	1/291	12/350	7/302	3/343	3/63	4/395	10/407	0/68	70/3650
	0.4%	0.0%	4.1%	2.6%	0.9%	0.3%	3.4%	2.3%	0.9%	4.8%	1.0%	2.5%	0.0%	1.9%
	0.0%-1.1%	0.0%-0.0%	2.8%-5.4%	0.0%-6.7%	0.0%-1.8%	0.0%-0.9%	1.8%-5.0%	0.9%-3.7%	0.0%-1.7%	0.3%-9.2%	0.2%-1.8%	1.2%-3.7%	0.0%-0.0%	1.5%-2.3%
Pfubp1-Asp1934His	0/47	1/69	1/267	0/9	1/69	0/52	0/114	3/72	1/91	0/12	0/126	0/58	0/4	7/990
	0.0%	1.4%	0.4%	0.0%	1.4%	0.0%	0.0%	4.2%	1.1%	0.0%	0.0%	0.0%	0.0%	0.7%
	0.0%-0.0%	0.0%-3.8%	0.0%-1.0%	0.0%-0.0%	0.0%-3.8%	0.0%-0.0%	0.0%-0.0%	0.3%-8.0%	0.0%-2.9%	0.0%-0.0%	0.0%-0.0%	0.0%-0.0%	0.0%-0.0%	0.3%-1.1%
Pfubp1-Asp267Glu	0/19	0/58	0/254	0/3	0/109	0/48	1/117	0/22	1/137	1/31	0/100	0/12	0/5	3/915
	0.0%	0.0%	0.0%	0.0%	0.0%	0.0%	0.9%	0.0%	0.7%	3.2%	0.0%	0.0%	0.0%	0.3%
	0.0%-0.0%	0.0%-0.0%	0.0%-0.0%	0.0%-0.0%	0.0%-0.0%	0.0%-0.0%	0.0%-2.3%	0.0%-0.0%	0.0%-1.9%	0.0%-8.4%	0.0%-0.0%	0.0%-0.0%	0.0%-0.0%	0.0%-0.6%
Pfubp1-Asp2756Gly	0/36	0/76	2/319	0/16	0/142	1/67	0/139	0/58	0/169	0/27	1/144	0/26	0/27	4/1246
	0.0%	0.0%	0.6%	0.0%	0.0%	1.5%	0.0%	0.0%	0.0%	0.0%	0.7%	0.0%	0.0%	0.3%
	0.0%-0.0%	0.0%-0.0%	0.0%-1.4%	0.0%-0.0%	0.0%-0.0%	0.0%-3.9%	0.0%-0.0%	0.0%-0.0%	0.0%-0.0%	0.0%-0.0%	0.0%-1.8%	0.0%-0.0%	0.0%-0.0%	0.1%-0.6%
Pfubp1-Asp280Ala	0/19	14/58	50/254	2/3	23/109	6/48	15/117	0/22	13/137	1/31	3/100	2/12	2/5	131/915
	0.0%	24.1%	19.7%	66.7%	21.1%	12.5%	12.8%	0.0%	9.5%	3.2%	3.0%	16.7%	40.0%	14.3%

	0.0%-0.0%	14.9%-33.4%	15.6%-23.8%	21.9%-100.0%	14.7%-27.5%	4.6%-20.4%	7.7%-17.9%	0.0%-0.0%	5.4%-13.6%	0.0%-8.4%	0.2%-5.8%	0.0%-34.4%	4.0%-76.0%	12.4%-16.2%
Pfubp1-Asp2876Ala	0/3	0/55	5/252	0/1	0/106	0/51	0/113	0/21	1/122	0/23	0/90	0/15	0/2	6/854
	0.0%	0.0%	2.0%	0.0%	0.0%	0.0%	0.0%	0.0%	0.8%	0.0%	0.0%	0.0%	0.0%	0.7%
	0.0%-0.0%	0.0%-0.0%	0.5%-3.4%	0.0%-0.0%	0.0%-0.0%	0.0%-0.0%	0.0%-0.0%	0.0%-0.0%	0.0%-2.2%	0.0%-0.0%	0.0%-0.0%	0.0%-0.0%	0.0%-0.0%	0.2%-1.2%
Pfubp1-Asp287Asn	0/19	1/58	5/254	0/3	2/109	0/48	0/118	0/22	5/137	1/31	2/101	0/12	0/5	16/917
	0.0%	1.7%	2.0%	0.0%	1.8%	0.0%	0.0%	0.0%	3.6%	3.2%	2.0%	0.0%	0.0%	1.7%
	0.0%-0.0%	0.0%-4.5%	0.5%-3.4%	0.0%-0.0%	0.0%-3.9%	0.0%-0.0%	0.0%-0.0%	0.0%-0.0%	1.0%-6.3%	0.0%-8.4%	0.0%-4.3%	0.0%-0.0%	0.0%-0.0%	1.0%-2.5%
Pfubp1-Asp2947Tyr	0/170	0/165	1/575	0/24	0/189	0/176	0/224	0/188	0/299	0/52	0/327	0/162	2/44	3/2595
	0.0%	0.0%	0.2%	0.0%	0.0%	0.0%	0.0%	0.0%	0.0%	0.0%	0.0%	0.0%	4.5%	0.1%
	0.0%-0.0%	0.0%-0.0%	0.0%-0.5%	0.0%-0.0%	0.0%-0.0%	0.0%-0.0%	0.0%-0.0%	0.0%-0.0%	0.0%-0.0%	0.0%-0.0%	0.0%-0.0%	0.0%-0.0%	0.0%-9.7%	0.0%-0.2%
Pfubp1-Asp3136Asn	0/126	0/164	2/530	0/12	0/171	0/159	0/188	0/144	1/277	0/33	0/267	0/173	0/25	3/2269
	0.0%	0.0%	0.4%	0.0%	0.0%	0.0%	0.0%	0.0%	0.4%	0.0%	0.0%	0.0%	0.0%	0.1%
	0.0%-0.0%	0.0%-0.0%	0.0%-0.8%	0.0%-0.0%	0.0%-0.0%	0.0%-0.0%	0.0%-0.0%	0.0%-0.0%	0.0%-1.0%	0.0%-0.0%	0.0%-0.0%	0.0%-0.0%	0.0%-0.0%	0.0%-0.3%
Pfubp1-Asp360Asn	0/5	0/14	3/116	no coverage	1/10	0/11	2/49	0/9	1/59	0/4	3/40	0/8	0/1	10/326
	0.0%	0.0%	2.6%	no coverage	10.0%	0.0%	4.1%	0.0%	1.7%	0.0%	7.5%	0.0%	0.0%	3.1%
	0.0%-0.0%	0.0%-0.0%	0.2%-5.0%	no coverage	0.0%-25.6%	0.0%-0.0%	0.0%-8.7%	0.0%-0.0%	0.0%-4.5%	0.0%-0.0%	0.6%-14.4%	0.0%-0.0%	0.0%-0.0%	1.5%-4.6%
Pfubp1-Asp734Val	0/180	0/197	0/566	0/27	0/202	0/190	0/252	1/216	2/301	0/55	0/348	0/248	0/44	3/2826
	0.0%	0.0%	0.0%	0.0%	0.0%	0.0%	0.0%	0.5%	0.7%	0.0%	0.0%	0.0%	0.0%	0.1%
	0.0%-0.0%	0.0%-0.0%	0.0%-0.0%	0.0%-0.0%	0.0%-0.0%	0.0%-0.0%	0.0%-0.0%	0.0%-1.2%	0.0%-1.4%	0.0%-0.0%	0.0%-0.0%	0.0%-0.0%	0.0%-0.0%	0.0%-0.2%
Pfubp1-Asp777Gly	6/112	8/95	7/346	0/5	5/83	11/92	4/133	5/125	19/179	0/14	3/204	6/136	0/4	74/1528
	5.4%	8.4%	2.0%	0.0%	6.0%	12.0%	3.0%	4.0%	10.6%	0.0%	1.5%	4.4%	0.0%	4.8%
	1.9%-8.9%	3.7%-13.1%	0.8%-3.3%	0.0%-0.0%	1.7%-10.3%	6.4%-17.5%	0.6%-5.4%	1.1%-6.9%	6.8%-14.4%	0.0%-0.0%	0.1%-2.9%	1.5%-7.3%	0.0%-0.0%	3.9%-5.7%
Pfubp1-Asp782Gly	6/112	8/95	7/346	0/5	5/83	11/92	4/133	5/125	19/179	0/14	2/204	6/136	0/4	73/1528
	5.4%	8.4%	2.0%	0.0%	6.0%	12.0%	3.0%	4.0%	10.6%	0.0%	1.0%	4.4%	0.0%	4.8%
	1.9%-8.9%	3.7%-13.1%	0.8%-3.3%	0.0%-0.0%	1.7%-10.3%	6.4%-17.5%	0.6%-5.4%	1.1%-6.9%	6.8%-14.4%	0.0%-0.0%	0.0%-2.1%	1.5%-7.3%	0.0%-0.0%	3.9%-5.7%
Pfubp1-Asp786Gly	6/112	8/95	7/346	0/5	5/83	11/92	4/133	5/125	19/179	0/14	2/204	6/136	0/4	73/1528
	5.4%	8.4%	2.0%	0.0%	6.0%	12.0%	3.0%	4.0%	10.6%	0.0%	1.0%	4.4%	0.0%	4.8%
	1.9%-8.9%	3.7%-13.1%	0.8%-3.3%	0.0%-0.0%	1.7%-10.3%	6.4%-17.5%	0.6%-5.4%	1.1%-6.9%	6.8%-14.4%	0.0%-0.0%	0.0%-2.1%	1.5%-7.3%	0.0%-0.0%	3.9%-5.7%
Pfubp1-Asp790Lys	6/112	8/95	7/346	0/5	5/83	11/92	4/133	5/125	19/179	0/14	2/204	6/136	0/4	73/1528
	5.4%	8.4%	2.0%	0.0%	6.0%	12.0%	3.0%	4.0%	10.6%	0.0%	1.0%	4.4%	0.0%	4.8%
	1.9%-8.9%	3.7%-13.1%	0.8%-3.3%	0.0%-0.0%	1.7%-10.3%	6.4%-17.5%	0.6%-5.4%	1.1%-6.9%	6.8%-14.4%	0.0%-0.0%	0.0%-2.1%	1.5%-7.3%	0.0%-0.0%	3.9%-5.7%
Pfubp1-Asp791Asn	0/112	0/95	12/346	0/5	0/83	0/92	0/133	0/125	1/179	0/14	1/204	3/136	0/4	17/1528
	0.0%	0.0%	3.5%	0.0%	0.0%	0.0%	0.0%	0.0%	0.6%	0.0%	0.5%	2.2%	0.0%	1.1%
	0.0%-0.0%	0.0%-0.0%	1.9%-5.1%	0.0%-0.0%	0.0%-0.0%	0.0%-0.0%	0.0%-0.0%	0.0%-0.0%	0.0%-1.5%	0.0%-0.0%	0.0%-1.3%	0.1%-4.3%	0.0%-0.0%	0.7%-1.6%
Pfubp1-Asp796Asn	6/112	8/95	7/346	0/5	5/83	11/92	4/133	5/125	15/179	0/14	2/204	6/136	0/4	69/1528
	5.4%	8.4%	2.0%	0.0%	6.0%	12.0%	3.0%	4.0%	8.4%	0.0%	1.0%	4.4%	0.0%	4.5%
	1.9%-8.9%	3.7%-13.1%	0.8%-3.3%	0.0%-0.0%	1.7%-10.3%	6.4%-17.5%	0.6%-5.4%	1.1%-6.9%	5.0%-11.8%	0.0%-0.0%	0.0%-2.1%	1.5%-7.3%	0.0%-0.0%	3.6%-5.4%
Pfubp1-Asp800Asn	0/112	0/95	12/346	0/5	0/83	0/92	1/133	0/125	5/179	0/14	1/204	3/136	0/4	22/1528
	0.0%	0.0%	3.5%	0.0%	0.0%	0.0%	0.8%	0.0%	2.8%	0.0%	0.5%	2.2%	0.0%	1.4%
	0.0%-0.0%	0.0%-0.0%	1.9%-5.1%	0.0%-0.0%	0.0%-0.0%	0.0%-0.0%	0.0%-2.0%	0.0%-0.0%	0.8%-4.8%	0.0%-0.0%	0.0%-1.3%	0.1%-4.3%	0.0%-0.0%	0.9%-1.9%
Pfubp1-Cys2818Gly	4/167	0/165	18/558	1/19	5/179	3/183	12/215	4/169	0/271	2/49	8/285	4/181	1/38	62/2479
	2.4%	0.0%	3.2%	5.3%	2.8%	1.6%	5.6%	2.4%	0.0%	4.1%	2.8%	2.2%	2.6%	2.5%
	0.4%-4.3%	0.0%-0.0%	2.0%-4.5%	0.0%-13.7%	0.8%-4.8%	0.1%-3.2%	3.0%-8.2%	0.4%-4.3%	0.0%-0.0%	0.0%-8.7%	1.2%-4.4%	0.4%-4.0%	0.0%-6.9%	2.0%-3.0%
Pfubp1-Cys2818Tyr	1/167	3/165	23/558	2/19	7/179	8/183	5/215	6/169	18/271	0/49	17/285	5/181	6/38	101/2479

	0.6%	1.8%	4.1%	10.5%	3.9%	4.4%	2.3%	3.6%	6.6%	0.0%	6.0%	2.8%	15.8%	4.1%
	0.0%-1.6%	0.1%-3.5%	2.7%-5.5%	0.0%-22.1%	1.5%-6.3%	1.9%-6.9%	0.6%-4.0%	1.2%-5.9%	4.2%-9.1%	0.0%-0.0%	3.7%-8.3%	0.8%-4.8%	6.1%-25.5%	3.4%-4.7%
Pfubp1-Gln1330Leu	0/60	0/76	7/362	0/8	0/128	0/65	3/130	0/85	2/155	0/27	0/172	0/43	0/12	12/1323
	0.0%	0.0%	1.9%	0.0%	0.0%	0.0%	2.3%	0.0%	1.3%	0.0%	0.0%	0.0%	0.0%	0.9%
	0.0%-0.0%	0.0%-0.0%	0.7%-3.1%	0.0%-0.0%	0.0%-0.0%	0.0%-0.0%	0.1%-4.5%	0.0%-0.0%	0.0%-2.8%	0.0%-0.0%	0.0%-0.0%	0.0%-0.0%	0.0%-0.0%	0.5%-1.3%
Pfubp1-Gln1885Pro	0/243	0/273	0/622	0/34	0/227	1/277	1/337	0/291	0/330	2/59	0/390	1/388	0/63	5/3534
	0.0%	0.0%	0.0%	0.0%	0.0%	0.4%	0.3%	0.0%	0.0%	3.4%	0.0%	0.3%	0.0%	0.1%
	0.0%-0.0%	0.0%-0.0%	0.0%-0.0%	0.0%-0.0%	0.0%-0.0%	0.0%-1.0%	0.0%-0.8%	0.0%-0.0%	0.0%-0.0%	0.0%-7.3%	0.0%-0.0%	0.0%-0.7%	0.0%-0.0%	0.0%-0.2%
Pfubp1-Gln2914Arg	0/123	6/140	1/521	0/23	0/172	0/148	0/201	0/156	1/255	0/48	0/273	0/122	0/38	8/2220
	0.0%	4.3%	0.2%	0.0%	0.0%	0.0%	0.0%	0.0%	0.4%	0.0%	0.0%	0.0%	0.0%	0.4%
	0.0%-0.0%	1.5%-7.1%	0.0%-0.5%	0.0%-0.0%	0.0%-0.0%	0.0%-0.0%	0.0%-0.0%	0.0%-0.0%	0.0%-1.0%	0.0%-0.0%	0.0%-0.0%	0.0%-0.0%	0.0%-0.0%	0.2%-0.6%
Pfubp1-Gln740Arg	3/180	0/197	1/566	0/27	3/202	0/190	7/252	0/216	0/301	1/55	6/348	1/248	0/44	22/2826
	1.7%	0.0%	0.2%	0.0%	1.5%	0.0%	2.8%	0.0%	0.0%	1.8%	1.7%	0.4%	0.0%	0.8%
	0.1%-3.2%	0.0%-0.0%	0.0%-0.5%	0.0%-0.0%	0.1%-2.9%	0.0%-0.0%	1.1%-4.5%	0.0%-0.0%	0.0%-0.0%	0.0%-4.8%	0.6%-2.9%	0.0%-1.1%	0.0%-0.0%	0.5%-1.1%
Pfubp1-Gln740Lys	0/180	0/197	1/566	0/27	0/202	0/190	2/252	1/216	0/301	0/55	0/348	0/248	0/44	4/2826
	0.0%	0.0%	0.2%	0.0%	0.0%	0.0%	0.8%	0.5%	0.0%	0.0%	0.0%	0.0%	0.0%	0.1%
	0.0%-0.0%	0.0%-0.0%	0.0%-0.5%	0.0%-0.0%	0.0%-0.0%	0.0%-0.0%	0.0%-1.7%	0.0%-1.2%	0.0%-0.0%	0.0%-0.0%	0.0%-0.0%	0.0%-0.0%	0.0%-0.0%	0.0%-0.3%
Pfubp1-Glu1011Lys	0/8	0/66	5/259	0/2	0/123	0/54	2/118	0/39	1/138	0/19	2/106	1/16	0/12	11/960
	0.0%	0.0%	1.9%	0.0%	0.0%	0.0%	1.7%	0.0%	0.7%	0.0%	1.9%	6.2%	0.0%	1.1%
	0.0%-0.0%	0.0%-0.0%	0.5%-3.3%	0.0%-0.0%	0.0%-0.0%	0.0%-0.0%	0.0%-3.6%	0.0%-0.0%	0.0%-1.9%	0.0%-0.0%	0.0%-4.1%	0.0%-16.2%	0.0%-0.0%	0.6%-1.7%
Pfubp1-Glu1085Lys	0/30	0/72	3/311	0/4	0/128	0/57	0/127	0/76	0/137	0/22	0/117	0/22	0/13	3/1116
	0.0%	0.0%	1.0%	0.0%	0.0%	0.0%	0.0%	0.0%	0.0%	0.0%	0.0%	0.0%	0.0%	0.3%
	0.0%-0.0%	0.0%-0.0%	0.1%-1.9%	0.0%-0.0%	0.0%-0.0%	0.0%-0.0%	0.0%-0.0%	0.0%-0.0%	0.0%-0.0%	0.0%-0.0%	0.0%-0.0%	0.0%-0.0%	0.0%-0.0%	0.0%-0.5%
Pfubp1-Glu111Asp	2/95	0/102	2/481	0/1	1/116	0/106	0/135	0/101	1/243	0/28	0/284	0/66	0/3	6/1761
	2.1%	0.0%	0.4%	0.0%	0.9%	0.0%	0.0%	0.0%	0.4%	0.0%	0.0%	0.0%	0.0%	0.3%
	0.0%-4.5%	0.0%-0.0%	0.0%-0.9%	0.0%-0.0%	0.0%-2.3%	0.0%-0.0%	0.0%-0.0%	0.0%-0.0%	0.0%-1.1%	0.0%-0.0%	0.0%-0.0%	0.0%-0.0%	0.0%-0.0%	0.1%-0.6%
Pfubp1-Glu1613Asp	0/3	1/65	1/222	0/1	0/111	1/51	0/115	0/29	0/80	0/10	0/61	0/18	0/9	3/775
	0.0%	1.5%	0.5%	0.0%	0.0%	2.0%	0.0%	0.0%	0.0%	0.0%	0.0%	0.0%	0.0%	0.4%
	0.0%-0.0%	0.0%-4.0%	0.0%-1.2%	0.0%-0.0%	0.0%-0.0%	0.0%-5.2%	0.0%-0.0%	0.0%-0.0%	0.0%-0.0%	0.0%-0.0%	0.0%-0.0%	0.0%-0.0%	0.0%-0.0%	0.0%-0.8%
Pfubp1-Glu1877Lys	0/251	0/278	0/629	0/39	0/234	0/291	0/350	3/302	0/343	1/63	2/395	0/407	0/68	6/3650
	0.0%	0.0%	0.0%	0.0%	0.0%	0.0%	0.0%	1.0%	0.0%	1.6%	0.5%	0.0%	0.0%	0.2%
	0.0%-0.0%	0.0%-0.0%	0.0%-0.0%	0.0%-0.0%	0.0%-0.0%	0.0%-0.0%	0.0%-0.0%	0.1%-1.9%	0.0%-0.0%	0.0%-4.2%	0.0%-1.1%	0.0%-0.0%	0.0%-0.0%	0.1%-0.3%
Pfubp1-Glu1915Lys	173/176	187/194	527/533	19/19	186/187	195/200	219/227	184/186	262/268	36/38	292/296	220/222	33/33	2533/2579
	98.3%	96.4%	98.9%	100.0%	99.5%	97.5%	96.5%	98.9%	97.8%	94.7%	98.6%	99.1%	100.0%	98.2%
	96.7%-99.9%	94.2%-98.6%	98.1%-99.6%	100.0%-100.0%	98.6%-100.0%	95.7%-99.3%	94.5%-98.5%	97.7%-100.0%	96.3%-99.2%	88.8%-100.0%	97.5%-99.8%	98.1%-100.0%	00.0%-100.0%	97.8%-98.6%
Pfubp1-Glu1922Lys	53/171	39/182	205/520	11/18	62/180	44/185	79/211	36/181	85/256	6/33	99/287	64/199	4/32	787/2455
	31.0%	21.4%	39.4%	61.1%	34.4%	23.8%	37.4%	19.9%	33.2%	18.2%	34.5%	32.2%	12.5%	32.1%
	25.2%-36.8%	16.4%-26.4%	35.9%-42.9%	42.2%-80.0%	28.6%-40.3%	18.6%-28.9%	32.0%-42.9%	15.0%-24.8%	28.4%-38.0%	7.1%-29.2%	29.9%-39.1%	26.7%-37.6%	2.9%-22.1%	30.5%-33.6%
Pfubp1-Glu1929Lys	3/47	8/69	27/265	1/9	2/69	2/52	11/114	7/72	17/91	1/12	16/126	2/58	1/4	98/988
	6.4%	11.6%	10.2%	11.1%	2.9%	3.8%	9.6%	9.7%	18.7%	8.3%	12.7%	3.4%	25.0%	9.9%
	0.5%-12.2%	5.3%-17.9%	7.1%-13.2%	0.0%-28.3%	0.0%-6.2%	0.0%-8.2%	5.1%-14.2%	4.0%-15.5%	12.0%-25.4%	0.0%-21.5%	7.8%-17.6%	0.0%-7.4%	0.0%-60.6%	8.4%-11.5%
Pfubp1-Glu277Gln	0/19	0/58	0/254	0/3	0/109	0/48	4/117	0/22	3/137	0/31	0/100	0/12	0/5	7/915
	0.0%	0.0%	0.0%	0.0%	0.0%	0.0%	3.4%	0.0%	2.2%	0.0%	0.0%	0.0%	0.0%	0.8%
	0.0%-0.0%	0.0%-0.0%	0.0%-0.0%	0.0%-0.0%	0.0%-0.0%	0.0%-0.0%	0.7%-6.2%	0.0%-0.0%	0.1%-4.2%	0.0%-0.0%	0.0%-0.0%	0.0%-0.0%	0.0%-0.0%	0.3%-1.2%

Pfubp1-Glu2860Val	0/169	0/168	1/571	0/19	0/187	1/191	2/223	0/169	1/288	0/50	2/304	0/186	0/40	7/2565
	0.0%	0.0%	0.2%	0.0%	0.0%	0.5%	0.9%	0.0%	0.3%	0.0%	0.7%	0.0%	0.0%	0.3%
	0.0%-0.0%	0.0%-0.0%	0.0%-0.5%	0.0%-0.0%	0.0%-0.0%	0.0%-1.4%	0.0%-1.9%	0.0%-0.0%	0.0%-0.9%	0.0%-0.0%	0.0%-1.4%	0.0%-0.0%	0.0%-0.0%	0.1%-0.4%
Pfubp1-Glu317Val	1/100	2/137	18/475	0/13	4/161	0/141	3/170	2/123	1/226	1/42	7/251	0/99	0/20	39/1958
	1.0%	1.5%	3.8%	0.0%	2.5%	0.0%	1.8%	1.6%	0.4%	2.4%	2.8%	0.0%	0.0%	2.0%
	0.0%-2.6%	0.0%-3.1%	2.3%-5.2%	0.0%-0.0%	0.5%-4.5%	0.0%-0.0%	0.1%-3.4%	0.0%-3.5%	0.0%-1.2%	0.0%-6.3%	1.1%-4.5%	0.0%-0.0%	0.0%-0.0%	1.5%-2.5%
Pfubp1-Glu737Val	0/180	1/197	0/566	0/27	1/202	0/190	0/252	0/216	0/301	0/55	2/348	1/248	0/44	5/2826
	0.0%	0.5%	0.0%	0.0%	0.5%	0.0%	0.0%	0.0%	0.0%	0.0%	0.6%	0.4%	0.0%	0.2%
	0.0%-0.0%	0.0%-1.3%	0.0%-0.0%	0.0%-0.0%	0.0%-1.3%	0.0%-0.0%	0.0%-0.0%	0.0%-0.0%	0.0%-0.0%	0.0%-0.0%	0.0%-1.2%	0.0%-1.1%	0.0%-0.0%	0.0%-0.3%
Pfubp1-Glu805Lys	0/187	0/177	0/503	0/15	0/145	0/189	3/209	0/215	0/247	0/30	0/300	0/269	0/18	3/2504
	0.0%	0.0%	0.0%	0.0%	0.0%	0.0%	1.4%	0.0%	0.0%	0.0%	0.0%	0.0%	0.0%	0.1%
	0.0%-0.0%	0.0%-0.0%	0.0%-0.0%	0.0%-0.0%	0.0%-0.0%	0.0%-0.0%	0.1%-2.8%	0.0%-0.0%	0.0%-0.0%	0.0%-0.0%	0.0%-0.0%	0.0%-0.0%	0.0%-0.0%	0.0%-0.2%
Pfubp1-Gly1009Ala	0/8	0/66	0/259	0/2	0/123	0/54	5/118	0/39	0/138	0/19	0/106	0/16	0/12	5/960
	0.0%	0.0%	0.0%	0.0%	0.0%	0.0%	4.2%	0.0%	0.0%	0.0%	0.0%	0.0%	0.0%	0.5%
	0.0%-0.0%	0.0%-0.0%	0.0%-0.0%	0.0%-0.0%	0.0%-0.0%	0.0%-0.0%	1.2%-7.3%	0.0%-0.0%	0.0%-0.0%	0.0%-0.0%	0.0%-0.0%	0.0%-0.0%	0.0%-0.0%	0.1%-0.9%
Pfubp1-Gly1697Asp	0/103	0/110	0/455	0/5	1/139	0/113	2/149	0/104	0/204	0/29	0/239	0/97	0/15	3/1762
	0.0%	0.0%	0.0%	0.0%	0.7%	0.0%	1.3%	0.0%	0.0%	0.0%	0.0%	0.0%	0.0%	0.2%
	0.0%-0.0%	0.0%-0.0%	0.0%-0.0%	0.0%-0.0%	0.0%-1.9%	0.0%-0.0%	0.0%-2.9%	0.0%-0.0%	0.0%-0.0%	0.0%-0.0%	0.0%-0.0%	0.0%-0.0%	0.0%-0.0%	0.0%-0.3%
Pfubp1-Gly170Ala	5/192	4/194	11/561	0/18	2/182	1/207	2/229	3/185	5/295	0/41	2/352	1/211	0/40	36/2707
	2.6%	2.1%	2.0%	0.0%	1.1%	0.5%	0.9%	1.6%	1.7%	0.0%	0.6%	0.5%	0.0%	1.3%
	0.7%-4.5%	0.4%-3.7%	1.0%-2.9%	0.0%-0.0%	0.0%-2.4%	0.0%-1.3%	0.0%-1.9%	0.1%-3.1%	0.5%-2.9%	0.0%-0.0%	0.0%-1.2%	0.0%-1.3%	0.0%-0.0%	1.0%-1.7%
Pfubp1-Gly1900Arg	2/243	1/273	0/622	0/34	0/227	0/277	1/336	0/291	0/330	0/59	0/390	2/388	0/63	6/3533
	0.8%	0.4%	0.0%	0.0%	0.0%	0.0%	0.3%	0.0%	0.0%	0.0%	0.0%	0.5%	0.0%	0.2%
	0.0%-1.8%	0.0%-1.0%	0.0%-0.0%	0.0%-0.0%	0.0%-0.0%	0.0%-0.0%	0.0%-0.8%	0.0%-0.0%	0.0%-0.0%	0.0%-0.0%	0.0%-0.0%	0.0%-1.1%	0.0%-0.0%	0.1%-0.3%
Pfubp1-Gly2010Asp	0/118	0/133	0/521	0/16	0/166	0/144	0/182	0/126	3/269	0/45	0/289	0/96	0/33	3/2138
	0.0%	0.0%	0.0%	0.0%	0.0%	0.0%	0.0%	0.0%	1.1%	0.0%	0.0%	0.0%	0.0%	0.1%
	0.0%-0.0%	0.0%-0.0%	0.0%-0.0%	0.0%-0.0%	0.0%-0.0%	0.0%-0.0%	0.0%-0.0%	0.0%-0.0%	0.1%-2.2%	0.0%-0.0%	0.0%-0.0%	0.0%-0.0%	0.0%-0.0%	0.0%-0.3%
Pfubp1-Gly2806Cys	0/65	0/93	0/395	0/15	0/155	0/84	0/167	0/90	3/199	0/36	0/203	0/46	0/31	3/1579
	0.0%	0.0%	0.0%	0.0%	0.0%	0.0%	0.0%	0.0%	1.5%	0.0%	0.0%	0.0%	0.0%	0.2%
	0.0%-0.0%	0.0%-0.0%	0.0%-0.0%	0.0%-0.0%	0.0%-0.0%	0.0%-0.0%	0.0%-0.0%	0.0%-0.0%	0.1%-2.9%	0.0%-0.0%	0.0%-0.0%	0.0%-0.0%	0.0%-0.0%	0.0%-0.4%
Pfubp1-Gly2814Cys	4/167	1/165	27/558	0/19	4/179	5/183	9/215	7/169	10/271	0/49	19/285	4/181	2/38	92/2479
	2.4%	0.6%	4.8%	0.0%	2.2%	2.7%	4.2%	4.1%	3.7%	0.0%	6.7%	2.2%	5.3%	3.7%
	0.4%-4.3%	0.0%-1.6%	3.3%-6.3%	0.0%-0.0%	0.4%-4.1%	0.8%-4.7%	1.9%-6.4%	1.6%-6.7%	1.8%-5.6%	0.0%-0.0%	4.2%-9.1%	0.4%-4.0%	0.0%-11.2%	3.1%-4.3%
Pfubp1-His1107Tyr	0/76	0/97	0/420	0/8	0/145	1/88	0/147	0/95	1/207	0/31	1/222	0/39	0/21	3/1596
	0.0%	0.0%	0.0%	0.0%	0.0%	1.1%	0.0%	0.0%	0.5%	0.0%	0.5%	0.0%	0.0%	0.2%
	0.0%-0.0%	0.0%-0.0%	0.0%-0.0%	0.0%-0.0%	0.0%-0.0%	0.0%-3.0%	0.0%-0.0%	0.0%-0.0%	0.0%-1.3%	0.0%-0.0%	0.0%-1.2%	0.0%-0.0%	0.0%-0.0%	0.0%-0.4%
Pfubp1-His1203Arg	0/130	0/144	0/515	0/11	0/149	0/162	0/162	0/125	3/252	0/30	0/287	0/136	0/16	3/2119
	0.0%	0.0%	0.0%	0.0%	0.0%	0.0%	0.0%	0.0%	1.2%	0.0%	0.0%	0.0%	0.0%	0.1%
	0.0%-0.0%	0.0%-0.0%	0.0%-0.0%	0.0%-0.0%	0.0%-0.0%	0.0%-0.0%	0.0%-0.0%	0.0%-0.0%	0.1%-2.3%	0.0%-0.0%	0.0%-0.0%	0.0%-0.0%	0.0%-0.0%	0.0%-0.3%
Pfubp1-His1406Leu	0/12	0/64	2/213	0/1	1/109	1/52	0/111	0/44	0/90	0/17	0/71	0/14	0/11	4/809
	0.0%	0.0%	0.9%	0.0%	0.9%	1.9%	0.0%	0.0%	0.0%	0.0%	0.0%	0.0%	0.0%	0.5%
	0.0%-0.0%	0.0%-0.0%	0.0%-2.0%	0.0%-0.0%	0.0%-2.4%	0.0%-5.1%	0.0%-0.0%	0.0%-0.0%	0.0%-0.0%	0.0%-0.0%	0.0%-0.0%	0.0%-0.0%	0.0%-0.0%	0.1%-0.9%
Pfubp1-His162Asn	0/192	7/194	73/561	0/18	6/181	9/207	42/229	0/185	11/294	0/41	6/352	3/211	0/40	157/2705
	0.0%	3.6%	13.0%	0.0%	3.3%	4.3%	18.3%	0.0%	3.7%	0.0%	1.7%	1.4%	0.0%	5.8%

	0.0%-0.0%	1.4%-5.8%	10.7%-15.3%	0.0%-0.0%	1.1%-5.5%	2.0%-6.7%	14.1%-22.5%	0.0%-0.0%	1.9%-5.6%	0.0%-0.0%	0.6%-2.8%	0.1%-2.8%	0.0%-0.0%	5.1%-6.5%
Pfubp1-His1990Arg	0/81	0/113	5/462	0/11	0/148	0/113	0/157	0/105	0/224	0/31	0/221	0/66	0/23	5/1755
	0.0%	0.0%	1.1%	0.0%	0.0%	0.0%	0.0%	0.0%	0.0%	0.0%	0.0%	0.0%	0.0%	0.3%
	0.0%-0.0%	0.0%-0.0%	0.3%-1.9%	0.0%-0.0%	0.0%-0.0%	0.0%-0.0%	0.0%-0.0%	0.0%-0.0%	0.0%-0.0%	0.0%-0.0%	0.0%-0.0%	0.0%-0.0%	0.0%-0.0%	0.1%-0.5%
Pfubp1-His2001Pro	0/81	0/113	4/462	0/11	0/148	0/113	1/157	0/105	0/224	2/31	0/221	0/66	0/23	7/1755
	0.0%	0.0%	0.9%	0.0%	0.0%	0.0%	0.6%	0.0%	0.0%	6.5%	0.0%	0.0%	0.0%	0.4%
	0.0%-0.0%	0.0%-0.0%	0.2%-1.6%	0.0%-0.0%	0.0%-0.0%	0.0%-0.0%	0.0%-1.7%	0.0%-0.0%	0.0%-0.0%	0.0%-13.7%	0.0%-0.0%	0.0%-0.0%	0.0%-0.0%	0.2%-0.6%
Pfubp1-His224Gln	1/86	0/86	3/411	0/6	0/131	0/102	1/133	0/92	4/187	1/22	1/186	0/53	0/18	11/1513
	1.2%	0.0%	0.7%	0.0%	0.0%	0.0%	0.8%	0.0%	2.1%	4.5%	0.5%	0.0%	0.0%	0.7%
	0.0%-3.1%	0.0%-0.0%	0.0%-1.4%	0.0%-0.0%	0.0%-0.0%	0.0%-0.0%	0.0%-2.0%	0.0%-0.0%	0.4%-3.9%	0.0%-11.9%	0.0%-1.4%	0.0%-0.0%	0.0%-0.0%	0.4%-1.1%
Pfubp1-His2370Tyr	0/19	0/77	0/331	0/7	1/146	2/68	0/137	0/28	0/196	0/37	0/153	0/22	0/13	3/1234
	0.0%	0.0%	0.0%	0.0%	0.7%	2.9%	0.0%	0.0%	0.0%	0.0%	0.0%	0.0%	0.0%	0.2%
	0.0%-0.0%	0.0%-0.0%	0.0%-0.0%	0.0%-0.0%	0.0%-1.8%	0.0%-6.3%	0.0%-0.0%	0.0%-0.0%	0.0%-0.0%	0.0%-0.0%	0.0%-0.0%	0.0%-0.0%	0.0%-0.0%	0.0%-0.5%
Pfubp1-His2552Tyr	0/70	1/101	8/459	0/10	0/145	1/107	1/156	0/88	0/243	0/32	1/250	1/60	0/24	13/1745
	0.0%	1.0%	1.7%	0.0%	0.0%	0.9%	0.6%	0.0%	0.0%	0.0%	0.4%	1.7%	0.0%	0.7%
	0.0%-0.0%	0.0%-2.6%	0.7%-2.7%	0.0%-0.0%	0.0%-0.0%	0.0%-2.5%	0.0%-1.7%	0.0%-0.0%	0.0%-0.0%	0.0%-0.0%	0.0%-1.1%	0.0%-4.4%	0.0%-0.0%	0.4%-1.1%
Pfubp1-His2904Pro	0/131	0/145	0/537	0/23	0/181	0/160	3/209	0/159	0/277	0/53	0/289	0/125	0/40	3/2329
	0.0%	0.0%	0.0%	0.0%	0.0%	0.0%	1.4%	0.0%	0.0%	0.0%	0.0%	0.0%	0.0%	0.1%
	0.0%-0.0%	0.0%-0.0%	0.0%-0.0%	0.0%-0.0%	0.0%-0.0%	0.0%-0.0%	0.1%-2.8%	0.0%-0.0%	0.0%-0.0%	0.0%-0.0%	0.0%-0.0%	0.0%-0.0%	0.0%-0.0%	0.0%-0.3%
Pfubp1-His2940Tyr	0/123	0/140	2/521	0/23	0/172	0/148	0/201	0/156	0/255	0/48	2/273	0/122	0/38	4/2220
	0.0%	0.0%	0.4%	0.0%	0.0%	0.0%	0.0%	0.0%	0.0%	0.0%	0.7%	0.0%	0.0%	0.2%
	0.0%-0.0%	0.0%-0.0%	0.0%-0.8%	0.0%-0.0%	0.0%-0.0%	0.0%-0.0%	0.0%-0.0%	0.0%-0.0%	0.0%-0.0%	0.0%-0.0%	0.0%-1.6%	0.0%-0.0%	0.0%-0.0%	0.0%-0.3%
Pfubp1-His3074Arg	2/132	2/176	1/549	0/13	1/169	3/172	2/201	2/144	1/273	0/41	0/331	1/165	0/26	15/2392
	1.5%	1.1%	0.2%	0.0%	0.6%	1.7%	1.0%	1.4%	0.4%	0.0%	0.0%	0.6%	0.0%	0.6%
	0.0%-3.3%	0.0%-2.5%	0.0%-0.5%	0.0%-0.0%	0.0%-1.6%	0.1%-3.4%	0.0%-2.1%	0.0%-3.0%	0.0%-1.0%	0.0%-0.0%	0.0%-0.0%	0.0%-1.6%	0.0%-0.0%	0.4%-0.9%
Pfubp1-His321Pro	0/100	3/137	1/475	0/13	0/161	0/141	2/170	0/123	0/226	0/42	0/251	0/99	0/20	6/1958
	0.0%	2.2%	0.2%	0.0%	0.0%	0.0%	1.2%	0.0%	0.0%	0.0%	0.0%	0.0%	0.0%	0.3%
	0.0%-0.0%	0.1%-4.2%	0.0%-0.6%	0.0%-0.0%	0.0%-0.0%	0.0%-0.0%	0.0%-2.5%	0.0%-0.0%	0.0%-0.0%	0.0%-0.0%	0.0%-0.0%	0.0%-0.0%	0.0%-0.0%	0.1%-0.5%
Pfubp1-His504Tyr	1/20	0/71	0/294	0/7	0/127	0/61	0/129	0/29	0/169	0/29	2/129	0/21	0/15	3/1101
	5.0%	0.0%	0.0%	0.0%	0.0%	0.0%	0.0%	0.0%	0.0%	0.0%	1.6%	0.0%	0.0%	0.3%
	0.0%-13.0%	0.0%-0.0%	0.0%-0.0%	0.0%-0.0%	0.0%-0.0%	0.0%-0.0%	0.0%-0.0%	0.0%-0.0%	0.0%-0.0%	0.0%-0.0%	0.0%-3.3%	0.0%-0.0%	0.0%-0.0%	0.0%-0.5%
Pfubp1-Ile1094Leu	0/30	3/72	5/311	0/4	0/128	0/57	0/127	0/76	0/137	0/22	0/117	0/22	0/13	8/1116
	0.0%	4.2%	1.6%	0.0%	0.0%	0.0%	0.0%	0.0%	0.0%	0.0%	0.0%	0.0%	0.0%	0.7%
	0.0%-0.0%	0.3%-8.0%	0.4%-2.8%	0.0%-0.0%	0.0%-0.0%	0.0%-0.0%	0.0%-0.0%	0.0%-0.0%	0.0%-0.0%	0.0%-0.0%	0.0%-0.0%	0.0%-0.0%	0.0%-0.0%	0.3%-1.1%
Pfubp1-Ile1210Asn	0/161	2/171	0/562	0/17	0/183	0/185	0/209	0/149	2/299	0/41	0/343	0/174	0/38	4/2532
	0.0%	1.2%	0.0%	0.0%	0.0%	0.0%	0.0%	0.0%	0.7%	0.0%	0.0%	0.0%	0.0%	0.2%
	0.0%-0.0%	0.0%-2.5%	0.0%-0.0%	0.0%-0.0%	0.0%-0.0%	0.0%-0.0%	0.0%-0.0%	0.0%-0.0%	0.0%-1.4%	0.0%-0.0%	0.0%-0.0%	0.0%-0.0%	0.0%-0.0%	0.0%-0.3%
Pfubp1-Ile1874Val	0/180	0/167	2/564	0/21	0/193	0/188	0/223	1/182	0/294	0/53	0/324	0/159	0/45	3/2593
	0.0%	0.0%	0.4%	0.0%	0.0%	0.0%	0.0%	0.5%	0.0%	0.0%	0.0%	0.0%	0.0%	0.1%
	0.0%-0.0%	0.0%-0.0%	0.0%-0.8%	0.0%-0.0%	0.0%-0.0%	0.0%-0.0%	0.0%-0.0%	0.0%-1.5%	0.0%-0.0%	0.0%-0.0%	0.0%-0.0%	0.0%-0.0%	0.0%-0.0%	0.0%-0.2%
Pfubp1-Ile1980Thr	0/147	0/157	0/546	0/19	0/176	2/170	2/205	0/161	0/284	0/49	1/318	3/163	0/32	8/2427
	0.0%	0.0%	0.0%	0.0%	0.0%	1.2%	1.0%	0.0%	0.0%	0.0%	0.3%	1.8%	0.0%	0.3%
	0.0%-0.0%	0.0%-0.0%	0.0%-0.0%	0.0%-0.0%	0.0%-0.0%	0.0%-2.5%	0.0%-2.1%	0.0%-0.0%	0.0%-0.0%	0.0%-0.0%	0.0%-0.8%	0.1%-3.6%	0.0%-0.0%	0.1%-0.5%
Pfubp1-Ile2287Leu	0/92	0/124	1/489	0/13	0/153	0/120	3/170	0/112	3/245	0/41	0/241	0/61	0/29	7/1890

	0.0%	0.0%	0.2%	0.0%	0.0%	0.0%	1.8%	0.0%	1.2%	0.0%	0.0%	0.0%	0.0%	0.4%
	0.0%-0.0%	0.0%-0.0%	0.0%-0.5%	0.0%-0.0%	0.0%-0.0%	0.0%-0.0%	0.1%-3.4%	0.0%-0.0%	0.1%-2.4%	0.0%-0.0%	0.0%-0.0%	0.0%-0.0%	0.0%-0.0%	0.1%-0.6%
Pfubp1-Ile3137Val	0/126	0/164	0/530	0/12	0/171	0/159	0/188	15/144	0/277	0/33	0/267	0/173	0/25	15/2269
	0.0%	0.0%	0.0%	0.0%	0.0%	0.0%	0.0%	10.4%	0.0%	0.0%	0.0%	0.0%	0.0%	0.7%
	0.0%-0.0%	0.0%-0.0%	0.0%-0.0%	0.0%-0.0%	0.0%-0.0%	0.0%-0.0%	0.0%-0.0%	6.2%-14.6%	0.0%-0.0%	0.0%-0.0%	0.0%-0.0%	0.0%-0.0%	0.0%-0.0%	0.4%-0.9%
Pfubp1-Ile47Met	0/2	0/56	0/236	0/1	0/105	0/45	3/110	0/14	0/88	0/20	0/81	0/16	0/4	3/778
	0.0%	0.0%	0.0%	0.0%	0.0%	0.0%	2.7%	0.0%	0.0%	0.0%	0.0%	0.0%	0.0%	0.4%
	0.0%-0.0%	0.0%-0.0%	0.0%-0.0%	0.0%-0.0%	0.0%-0.0%	0.0%-0.0%	0.2%-5.3%	0.0%-0.0%	0.0%-0.0%	0.0%-0.0%	0.0%-0.0%	0.0%-0.0%	0.0%-0.0%	0.0%-0.8%
Pfubp1-Leu153Phe	1/192	0/194	0/560	0/18	1/181	1/207	0/229	0/185	0/294	0/41	0/352	0/211	0/40	3/2704
	0.5%	0.0%	0.0%	0.0%	0.6%	0.5%	0.0%	0.0%	0.0%	0.0%	0.0%	0.0%	0.0%	0.1%
	0.0%-1.4%	0.0%-0.0%	0.0%-0.0%	0.0%-0.0%	0.0%-1.5%	0.0%-1.3%	0.0%-0.0%	0.0%-0.0%	0.0%-0.0%	0.0%-0.0%	0.0%-0.0%	0.0%-0.0%	0.0%-0.0%	0.0%-0.2%
Pfubp1-Leu2258Ile	2/82	2/111	13/461	0/11	4/147	5/106	1/158	0/102	1/216	2/35	2/219	2/53	3/22	37/1723
	2.4%	1.8%	2.8%	0.0%	2.7%	4.7%	0.6%	0.0%	0.5%	5.7%	0.9%	3.8%	13.6%	2.1%
	0.0%-5.2%	0.0%-3.9%	1.6%-4.1%	0.0%-0.0%	0.5%-4.9%	1.3%-8.1%	0.0%-1.7%	0.0%-0.0%	0.0%-1.2%	0.0%-12.2%	0.0%-2.0%	0.0%-8.1%	1.6%-25.7%	1.6%-2.7%
Pfubp1-Leu879Phe	0/34	0/70	2/323	0/3	0/130	0/56	0/121	0/68	0/161	0/26	1/159	0/24	0/11	3/1186
	0.0%	0.0%	0.6%	0.0%	0.0%	0.0%	0.0%	0.0%	0.0%	0.0%	0.6%	0.0%	0.0%	0.3%
	0.0%-0.0%	0.0%-0.0%	0.0%-1.3%	0.0%-0.0%	0.0%-0.0%	0.0%-0.0%	0.0%-0.0%	0.0%-0.0%	0.0%-0.0%	0.0%-0.0%	0.0%-1.7%	0.0%-0.0%	0.0%-0.0%	0.0%-0.5%
Pfubp1-Lys1193Thr	1/130	6/144	37/515	0/11	7/149	7/162	12/162	5/125	11/252	0/30	2/287	7/136	0/16	95/2119
	0.8%	4.2%	7.2%	0.0%	4.7%	4.3%	7.4%	4.0%	4.4%	0.0%	0.7%	5.1%	0.0%	4.5%
	0.0%-2.0%	1.4%-6.9%	5.3%-9.1%	0.0%-0.0%	1.8%-7.5%	1.7%-6.9%	4.0%-10.8%	1.1%-6.9%	2.2%-6.5%	0.0%-0.0%	0.0%-1.5%	2.0%-8.3%	0.0%-0.0%	3.7%-5.2%
Pfubp1-Lys147Asn	1/192	0/194	1/560	0/18	0/181	0/207	0/229	1/185	0/294	0/41	3/352	0/211	0/40	6/2704
	0.5%	0.0%	0.2%	0.0%	0.0%	0.0%	0.0%	0.5%	0.0%	0.0%	0.9%	0.0%	0.0%	0.2%
	0.0%-1.4%	0.0%-0.0%	0.0%-0.5%	0.0%-0.0%	0.0%-0.0%	0.0%-0.0%	0.0%-0.0%	0.0%-1.4%	0.0%-0.0%	0.0%-0.0%	0.0%-1.7%	0.0%-0.0%	0.0%-0.0%	0.1%-0.4%
Pfubp1-Lys1705Asn	3/103	1/110	4/455	0/5	1/139	1/113	2/149	0/104	1/204	0/29	0/239	0/97	0/15	13/1762
	2.9%	0.9%	0.9%	0.0%	0.7%	0.9%	1.3%	0.0%	0.5%	0.0%	0.0%	0.0%	0.0%	0.7%
	0.2%-5.6%	0.0%-2.4%	0.2%-1.6%	0.0%-0.0%	0.0%-1.9%	0.0%-2.3%	0.0%-2.9%	0.0%-0.0%	0.0%-1.3%	0.0%-0.0%	0.0%-0.0%	0.0%-0.0%	0.0%-0.0%	0.4%-1.1%
Pfubp1-Lys1780Arg	0/1	0/69	0/251	0/3	0/118	0/54	4/113	0/3	0/108	0/18	0/77	0/19	0/9	4/843
	0.0%	0.0%	0.0%	0.0%	0.0%	0.0%	3.5%	0.0%	0.0%	0.0%	0.0%	0.0%	0.0%	0.5%
	0.0%-0.0%	0.0%-0.0%	0.0%-0.0%	0.0%-0.0%	0.0%-0.0%	0.0%-0.0%	0.7%-6.4%	0.0%-0.0%	0.0%-0.0%	0.0%-0.0%	0.0%-0.0%	0.0%-0.0%	0.0%-0.0%	0.1%-0.9%
Pfubp1-Lys1914Asn	173/176	187/194	527/533	19/19	186/187	195/200	219/227	184/186	262/268	36/38	292/296	220/222	33/33	2533/2579
	98.3%	96.4%	98.9%	100.0%	99.5%	97.5%	96.5%	98.9%	97.8%	94.7%	98.6%	99.1%	100.0%	98.2%
	96.7%-99.9%	94.2%-98.6%	98.1%-99.6%	100.0%-100.0%	98.6%-100.0%	95.7%-99.3%	94.5%-98.5%	97.7%-100.0%	96.3%-99.2%	88.8%-100.0%	97.5%-99.8%	98.1%-100.0%	00.0%-100.0%	97.8%-98.6%
Pfubp1-Lys1921Asn	55/171	39/182	205/520	11/18	62/180	44/185	79/211	37/181	86/256	6/33	99/287	64/199	4/32	791/2455
	32.2%	21.4%	39.4%	61.1%	34.4%	23.8%	37.4%	20.4%	33.6%	18.2%	34.5%	32.2%	12.5%	32.2%
	26.3%-38.0%	16.4%-26.4%	35.9%-42.9%	42.2%-80.0%	28.6%-40.3%	18.6%-28.9%	32.0%-42.9%	15.5%-25.4%	28.7%-38.4%	7.1%-29.2%	29.9%-39.1%	26.7%-37.6%	2.9%-22.1%	30.7%-33.8%
Pfubp1-Lys1928Asn	3/47	8/69	27/265	1/9	2/69	2/52	11/114	7/72	17/91	1/12	16/126	2/58	1/4	98/988
	6.4%	11.6%	10.2%	11.1%	2.9%	3.8%	9.6%	9.7%	18.7%	8.3%	12.7%	3.4%	25.0%	9.9%
	0.5%-12.2%	5.3%-17.9%	7.1%-13.2%	0.0%-28.3%	0.0%-6.2%	0.0%-8.2%	5.1%-14.2%	4.0%-15.5%	12.0%-25.4%	0.0%-21.5%	7.8%-17.6%	0.0%-7.4%	0.0%-60.6%	8.4%-11.5%
Pfubp1-Lys1936Glu	1/1	1/2	11/29	no coverage	0/1	1/2	8/23	9/13	1/2	0/1	5/6	1/1	1/1	39/82
	100.0%	50.0%	37.9%	no coverage	0.0%	50.0%	34.8%	69.2%	50.0%	0.0%	83.3%	100.0%	100.0%	47.6%
	100.0%-100.0%	0.0%-100.0%	23.1%-52.8%	no coverage	0.0%-0.0%	0.0%-100.0%	18.4%-51.1%	48.2%-90.3%	0.0%-100.0%	0.0%-0.0%	58.3%-100.0%	100.0%-100.0%	00.0%-100.0%	38.5%-56.6%
Pfubp1-Lys1949Asn	1/28	2/75	17/399	0/3	3/60	3/61	3/105	1/50	5/162	3/25	2/175	0/38	0/6	40/1187
	3.6%	2.7%	4.3%	0.0%	5.0%	4.9%	2.9%	2.0%	3.1%	12.0%	1.1%	0.0%	0.0%	3.4%
	0.0%-9.3%	0.0%-5.7%	2.6%-5.9%	0.0%-0.0%	0.4%-9.6%	0.4%-9.5%	0.2%-5.5%	0.0%-5.3%	0.9%-5.3%	1.3%-22.7%	0.0%-2.5%	0.0%-0.0%	0.0%-0.0%	2.5%-4.2%

Pfubp1-Lys1950Glu	0/28	1/75	0/399	0/3	0/60	1/61	0/105	0/50	0/162	0/25	0/175	1/38	0/6	3/1187
	0.0%	1.3%	0.0%	0.0%	0.0%	1.6%	0.0%	0.0%	0.0%	0.0%	0.0%	2.6%	0.0%	0.3%
	0.0%-0.0%	0.0%-3.5%	0.0%-0.0%	0.0%-0.0%	0.0%-0.0%	0.0%-4.3%	0.0%-0.0%	0.0%-0.0%	0.0%-0.0%	0.0%-0.0%	0.0%-0.0%	0.0%-6.9%	0.0%-0.0%	0.0%-0.5%
Pfubp1-Lys1956Asn	7/88	3/112	27/472	0/8	2/87	3/102	13/144	7/92	14/212	0/29	9/241	0/86	0/11	85/1684
	8.0%	2.7%	5.7%	0.0%	2.3%	2.9%	9.0%	7.6%	6.6%	0.0%	3.7%	0.0%	0.0%	5.0%
	3.2%-12.7%	0.2%-5.2%	4.0%-7.5%	0.0%-0.0%	0.0%-4.9%	0.2%-5.7%	5.1%-13.0%	3.1%-12.2%	3.8%-9.4%	0.0%-0.0%	1.7%-5.7%	0.0%-0.0%	0.0%-0.0%	4.2%-5.9%
Pfubp1-Lys1957Glu	1/88	0/112	0/472	0/8	0/87	0/102	0/144	0/92	0/212	0/29	0/241	3/86	0/11	4/1684
	1.1%	0.0%	0.0%	0.0%	0.0%	0.0%	0.0%	0.0%	0.0%	0.0%	0.0%	3.5%	0.0%	0.2%
	0.0%-3.0%	0.0%-0.0%	0.0%-0.0%	0.0%-0.0%	0.0%-0.0%	0.0%-0.0%	0.0%-0.0%	0.0%-0.0%	0.0%-0.0%	0.0%-0.0%	0.0%-0.0%	0.2%-6.7%	0.0%-0.0%	0.0%-0.4%
Pfubp1-Lys2809Asn	0/65	0/93	1/395	0/15	2/155	0/84	2/167	0/90	4/199	0/36	3/203	0/46	0/31	12/1579
	0.0%	0.0%	0.3%	0.0%	1.3%	0.0%	1.2%	0.0%	2.0%	0.0%	1.5%	0.0%	0.0%	0.8%
	0.0%-0.0%	0.0%-0.0%	0.0%-0.7%	0.0%-0.0%	0.0%-2.8%	0.0%-0.0%	0.0%-2.6%	0.0%-0.0%	0.4%-3.6%	0.0%-0.0%	0.1%-2.9%	0.0%-0.0%	0.0%-0.0%	0.4%-1.1%
Pfubp1-Lys2817Asn	3/167	0/165	16/558	1/19	4/179	3/183	12/215	4/169	0/271	2/49	8/285	2/181	1/38	56/2479
	1.8%	0.0%	2.9%	5.3%	2.2%	1.6%	5.6%	2.4%	0.0%	4.1%	2.8%	1.1%	2.6%	2.3%
	0.1%-3.5%	0.0%-0.0%	1.7%-4.0%	0.0%-13.7%	0.4%-4.1%	0.1%-3.2%	3.0%-8.2%	0.4%-4.3%	0.0%-0.0%	0.0%-8.7%	1.2%-4.4%	0.0%-2.4%	0.0%-6.9%	1.8%-2.7%
Pfubp1-Lys2843Asn	2/166	1/164	0/557	0/18	1/178	3/182	0/214	1/169	0/270	0/48	0/284	0/181	0/37	8/2468
	1.2%	0.6%	0.0%	0.0%	0.6%	1.6%	0.0%	0.6%	0.0%	0.0%	0.0%	0.0%	0.0%	0.3%
	0.0%-2.6%	0.0%-1.6%	0.0%-0.0%	0.0%-0.0%	0.0%-1.5%	0.1%-3.2%	0.0%-0.0%	0.0%-1.6%	0.0%-0.0%	0.0%-0.0%	0.0%-0.0%	0.0%-0.0%	0.0%-0.0%	0.1%-0.5%
Pfubp1-Lys3209Asn	0/26	0/76	0/347	0/12	0/140	3/72	0/146	0/41	0/188	0/26	0/153	0/32	0/21	3/1280
	0.0%	0.0%	0.0%	0.0%	0.0%	4.2%	0.0%	0.0%	0.0%	0.0%	0.0%	0.0%	0.0%	0.2%
	0.0%-0.0%	0.0%-0.0%	0.0%-0.0%	0.0%-0.0%	0.0%-0.0%	0.3%-8.0%	0.0%-0.0%	0.0%-0.0%	0.0%-0.0%	0.0%-0.0%	0.0%-0.0%	0.0%-0.0%	0.0%-0.0%	0.0%-0.5%
Pfubp1-Lys779Asn	6/112	8/95	7/346	0/5	5/83	11/92	4/133	5/125	15/179	0/14	3/204	6/136	0/4	70/1528
	5.4%	8.4%	2.0%	0.0%	6.0%	12.0%	3.0%	4.0%	8.4%	0.0%	1.5%	4.4%	0.0%	4.6%
	1.9%-8.9%	3.7%-13.1%	0.8%-3.3%	0.0%-0.0%	1.7%-10.3%	6.4%-17.5%	0.6%-5.4%	1.1%-6.9%	5.0%-11.8%	0.0%-0.0%	0.1%-2.9%	1.5%-7.3%	0.0%-0.0%	3.7%-5.5%
Pfubp1-Lys877Arg	0/34	0/70	5/323	0/3	0/130	0/56	5/121	0/68	0/161	0/26	4/159	0/24	0/11	14/1186
	0.0%	0.0%	1.5%	0.0%	0.0%	0.0%	4.1%	0.0%	0.0%	0.0%	2.5%	0.0%	0.0%	1.2%
	0.0%-0.0%	0.0%-0.0%	0.4%-2.7%	0.0%-0.0%	0.0%-0.0%	0.0%-0.0%	1.2%-7.1%	0.0%-0.0%	0.0%-0.0%	0.0%-0.0%	0.5%-4.6%	0.0%-0.0%	0.0%-0.0%	0.7%-1.7%
Pfubp1-Lys895Asn	0/15	1/64	6/221	0/2	0/80	0/54	4/104	0/20	1/98	0/21	0/76	0/17	0/5	12/777
	0.0%	1.6%	2.7%	0.0%	0.0%	0.0%	3.8%	0.0%	1.0%	0.0%	0.0%	0.0%	0.0%	1.5%
	0.0%-0.0%	0.0%-4.1%	0.9%-4.5%	0.0%-0.0%	0.0%-0.0%	0.0%-0.0%	0.7%-6.9%	0.0%-0.0%	0.0%-2.7%	0.0%-0.0%	0.0%-0.0%	0.0%-0.0%	0.0%-0.0%	0.8%-2.3%
Pfubp1-Lys906Asn	5/15	38/65	164/219	1/2	47/79	32/54	80/103	13/20	61/98	9/21	42/76	10/17	3/4	505/773
	33.3%	58.5%	74.9%	50.0%	59.5%	59.3%	77.7%	65.0%	62.2%	42.9%	55.3%	58.8%	75.0%	65.3%
	13.3%-53.4%	48.4%-68.5%	70.1%-79.7%	0.0%-100.0%	50.4%-68.6%	48.3%-70.3%	70.9%-84.4%	47.5%-82.5%	54.2%-70.3%	25.1%-60.6%	45.9%-64.6%	39.2%-78.5%	39.4%-100.0%	62.5%-68.1%
Pfubp1-Met1178Val	1/130	0/144	1/515	0/11	1/149	0/162	0/162	0/125	0/252	0/30	0/287	0/136	0/16	3/2119
	0.8%	0.0%	0.2%	0.0%	0.7%	0.0%	0.0%	0.0%	0.0%	0.0%	0.0%	0.0%	0.0%	0.1%
	0.0%-2.0%	0.0%-0.0%	0.0%-0.5%	0.0%-0.0%	0.0%-1.8%	0.0%-0.0%	0.0%-0.0%	0.0%-0.0%	0.0%-0.0%	0.0%-0.0%	0.0%-0.0%	0.0%-0.0%	0.0%-0.0%	0.0%-0.3%
Pfubp1-Met163Ile	0/192	29/194	154/561	1/18	42/181	29/207	89/229	0/185	47/294	0/41	29/352	5/211	1/40	426/2705
	0.0%	14.9%	27.5%	5.6%	23.2%	14.0%	38.9%	0.0%	16.0%	0.0%	8.2%	2.4%	2.5%	15.7%
	0.0%-0.0%	10.7%-19.2%	24.4%-30.6%	0.0%-14.4%	18.0%-28.4%	10.0%-18.0%	33.6%-44.2%	0.0%-0.0%	12.5%-19.5%	0.0%-0.0%	5.8%-10.6%	0.6%-4.1%	0.0%-6.6%	14.6%-16.9%
Pfubp1-Met193Leu	0/195	0/196	0/576	0/22	0/194	0/216	0/251	0/212	3/295	0/41	0/335	0/230	0/49	3/2812
	0.0%	0.0%	0.0%	0.0%	0.0%	0.0%	0.0%	0.0%	1.0%	0.0%	0.0%	0.0%	0.0%	0.1%
	0.0%-0.0%	0.0%-0.0%	0.0%-0.0%	0.0%-0.0%	0.0%-0.0%	0.0%-0.0%	0.0%-0.0%	0.0%-0.0%	0.1%-2.0%	0.0%-0.0%	0.0%-0.0%	0.0%-0.0%	0.0%-0.0%	0.0%-0.2%
Pfubp1-Met2618Val	0/8	3/76	7/314	0/10	8/143	3/67	11/143	0/23	2/186	0/33	5/132	1/23	0/22	40/1180
	0.0%	3.9%	2.2%	0.0%	5.6%	4.5%	7.7%	0.0%	1.1%	0.0%	3.8%	4.3%	0.0%	3.4%

	0.0%-0.0%	0.3%-7.6%	0.9%-3.6%	0.0%-0.0%	2.4%-8.8%	0.3%-8.6%	4.0%-11.4%	0.0%-0.0%	0.0%-2.3%	0.0%-0.0%	1.1%-6.5%	0.0%-11.3%	0.0%-0.0%	2.5%-4.3%
Pfubp1-Met3069Leu	0/126	2/162	2/537	0/13	1/163	0/159	0/191	0/134	1/269	0/40	0/320	0/143	0/25	6/2282
	0.0%	1.2%	0.4%	0.0%	0.6%	0.0%	0.0%	0.0%	0.4%	0.0%	0.0%	0.0%	0.0%	0.3%
	0.0%-0.0%	0.0%-2.7%	0.0%-0.8%	0.0%-0.0%	0.0%-1.6%	0.0%-0.0%	0.0%-0.0%	0.0%-0.0%	0.0%-1.0%	0.0%-0.0%	0.0%-0.0%	0.0%-0.0%	0.0%-0.0%	0.1%-0.4%
Pfubp1-Met3223Ile	0/48	0/90	16/435	0/15	0/161	0/92	0/167	0/75	0/242	0/36	0/236	1/45	0/37	17/1679
	0.0%	0.0%	3.7%	0.0%	0.0%	0.0%	0.0%	0.0%	0.0%	0.0%	0.0%	2.2%	0.0%	1.0%
	0.0%-0.0%	0.0%-0.0%	2.2%-5.2%	0.0%-0.0%	0.0%-0.0%	0.0%-0.0%	0.0%-0.0%	0.0%-0.0%	0.0%-0.0%	0.0%-0.0%	0.0%-0.0%	0.0%-5.8%	0.0%-0.0%	0.6%-1.4%
Pfubp1-Met967Ile	no coverage	0/16	3/119	no coverage	0/28	0/5	1/73	no coverage	2/26	0/3	0/5	0/1	no coverage	6/276
	no coverage	0.0%	2.5%	no coverage	0.0%	0.0%	1.4%	no coverage	7.7%	0.0%	0.0%	0.0%	no coverage	2.2%
	no coverage	0.0%-0.0%	0.2%-4.9%	no coverage	0.0%-0.0%	0.0%-0.0%	0.0%-3.6%	no coverage	0.0%-16.3%	0.0%-0.0%	0.0%-0.0%	0.0%-0.0%	no coverage	0.7%-3.6%
Pfubp1-Phe1407Leu	0/12	0/64	2/213	0/1	1/109	1/52	0/111	0/44	0/90	0/17	0/71	0/14	0/11	4/809
	0.0%	0.0%	0.9%	0.0%	0.9%	1.9%	0.0%	0.0%	0.0%	0.0%	0.0%	0.0%	0.0%	0.5%
	0.0%-0.0%	0.0%-0.0%	0.0%-2.0%	0.0%-0.0%	0.0%-2.4%	0.0%-5.1%	0.0%-0.0%	0.0%-0.0%	0.0%-0.0%	0.0%-0.0%	0.0%-0.0%	0.0%-0.0%	0.0%-0.0%	0.1%-0.9%
Pfubp1-Phe1739Tyr	0/37	0/77	3/340	0/11	1/147	1/71	0/146	1/64	3/199	0/30	1/168	0/29	1/28	11/1347
	0.0%	0.0%	0.9%	0.0%	0.7%	1.4%	0.0%	1.6%	1.5%	0.0%	0.6%	0.0%	3.6%	0.8%
	0.0%-0.0%	0.0%-0.0%	0.0%-1.7%	0.0%-0.0%	0.0%-1.8%	0.0%-3.7%	0.0%-0.0%	0.0%-4.1%	0.1%-2.9%	0.0%-0.0%	0.0%-1.6%	0.0%-0.0%	0.0%-9.3%	0.4%-1.2%
Pfubp1-Phe2333Leu	0/4	0/66	3/244	0/1	0/121	0/56	2/118	0/10	1/124	0/22	1/85	0/18	0/5	7/874
	0.0%	0.0%	1.2%	0.0%	0.0%	0.0%	1.7%	0.0%	0.8%	0.0%	1.2%	0.0%	0.0%	0.8%
	0.0%-0.0%	0.0%-0.0%	0.1%-2.4%	0.0%-0.0%	0.0%-0.0%	0.0%-0.0%	0.0%-3.6%	0.0%-0.0%	0.0%-2.1%	0.0%-0.0%	0.0%-3.1%	0.0%-0.0%	0.0%-0.0%	0.3%-1.3%
Pfubp1-Phe692Leu	2/76	0/97	0/461	0/15	0/155	0/96	0/165	1/103	4/241	0/40	0/229	0/53	0/29	7/1760
	2.6%	0.0%	0.0%	0.0%	0.0%	0.0%	0.0%	1.0%	1.7%	0.0%	0.0%	0.0%	0.0%	0.4%
	0.0%-5.7%	0.0%-0.0%	0.0%-0.0%	0.0%-0.0%	0.0%-0.0%	0.0%-0.0%	0.0%-0.0%	0.0%-2.6%	0.3%-3.0%	0.0%-0.0%	0.0%-0.0%	0.0%-0.0%	0.0%-0.0%	0.2%-0.6%
Pfubp1-Pro2903Gln	0/131	2/145	2/537	0/23	0/181	0/160	0/209	1/159	0/277	0/53	0/289	0/125	0/40	5/2329
	0.0%	1.4%	0.4%	0.0%	0.0%	0.0%	0.0%	0.6%	0.0%	0.0%	0.0%	0.0%	0.0%	0.2%
	0.0%-0.0%	0.0%-3.0%	0.0%-0.8%	0.0%-0.0%	0.0%-0.0%	0.0%-0.0%	0.0%-0.0%	0.0%-1.7%	0.0%-0.0%	0.0%-0.0%	0.0%-0.0%	0.0%-0.0%	0.0%-0.0%	0.1%-0.4%
Pfubp1-Pro3131Ser	0/124	0/139	0/474	0/3	0/140	0/146	0/167	15/138	0/249	0/26	0/221	0/163	0/14	15/2004
	0.0%	0.0%	0.0%	0.0%	0.0%	0.0%	0.0%	10.9%	0.0%	0.0%	0.0%	0.0%	0.0%	0.7%
	0.0%-0.0%	0.0%-0.0%	0.0%-0.0%	0.0%-0.0%	0.0%-0.0%	0.0%-0.0%	0.0%-0.0%	6.5%-15.2%	0.0%-0.0%	0.0%-0.0%	0.0%-0.0%	0.0%-0.0%	0.0%-0.0%	0.4%-1.1%
Pfubp1-Pro499Ser	0/20	1/71	1/294	0/7	1/127	1/61	0/129	0/29	0/169	0/29	0/129	0/21	0/15	4/1101
	0.0%	1.4%	0.3%	0.0%	0.8%	1.6%	0.0%	0.0%	0.0%	0.0%	0.0%	0.0%	0.0%	0.4%
	0.0%-0.0%	0.0%-3.7%	0.0%-0.9%	0.0%-0.0%	0.0%-2.1%	0.0%-4.3%	0.0%-0.0%	0.0%-0.0%	0.0%-0.0%	0.0%-0.0%	0.0%-0.0%	0.0%-0.0%	0.0%-0.0%	0.1%-0.7%
Pfubp1-Ser1103Leu	0/30	0/72	7/311	0/4	0/128	0/57	2/127	0/76	0/137	0/22	1/117	0/22	0/13	10/1116
	0.0%	0.0%	2.3%	0.0%	0.0%	0.0%	1.6%	0.0%	0.0%	0.0%	0.9%	0.0%	0.0%	0.9%
	0.0%-0.0%	0.0%-0.0%	0.9%-3.6%	0.0%-0.0%	0.0%-0.0%	0.0%-0.0%	0.0%-3.4%	0.0%-0.0%	0.0%-0.0%	0.0%-0.0%	0.0%-2.3%	0.0%-0.0%	0.0%-0.0%	0.4%-1.4%
Pfubp1-Ser1105Asn	0/76	0/97	2/420	0/8	0/145	0/88	1/147	0/95	0/207	0/31	0/222	0/39	0/21	3/1596
	0.0%	0.0%	0.5%	0.0%	0.0%	0.0%	0.7%	0.0%	0.0%	0.0%	0.0%	0.0%	0.0%	0.2%
	0.0%-0.0%	0.0%-0.0%	0.0%-1.0%	0.0%-0.0%	0.0%-0.0%	0.0%-0.0%	0.0%-1.8%	0.0%-0.0%	0.0%-0.0%	0.0%-0.0%	0.0%-0.0%	0.0%-0.0%	0.0%-0.0%	0.0%-0.4%
Pfubp1-Ser1121Tyr	0/25	0/61	2/334	no coverage	0/105	0/49	1/111	0/60	2/149	0/20	0/151	1/18	0/2	6/1085
	0.0%	0.0%	0.6%	no coverage	0.0%	0.0%	0.9%	0.0%	1.3%	0.0%	0.0%	5.6%	0.0%	0.6%
	0.0%-0.0%	0.0%-0.0%	0.0%-1.3%	no coverage	0.0%-0.0%	0.0%-0.0%	0.0%-2.4%	0.0%-0.0%	0.0%-2.9%	0.0%-0.0%	0.0%-0.0%	0.0%-14.4%	0.0%-0.0%	0.2%-0.9%
Pfubp1-Ser1185Thr	1/130	1/144	1/515	0/11	0/149	0/162	0/162	1/125	0/252	0/30	0/287	0/136	0/16	4/2119
	0.8%	0.7%	0.2%	0.0%	0.0%	0.0%	0.0%	0.8%	0.0%	0.0%	0.0%	0.0%	0.0%	0.2%
	0.0%-2.0%	0.0%-1.8%	0.0%-0.5%	0.0%-0.0%	0.0%-0.0%	0.0%-0.0%	0.0%-0.0%	0.0%-2.1%	0.0%-0.0%	0.0%-0.0%	0.0%-0.0%	0.0%-0.0%	0.0%-0.0%	0.0%-0.3%
Pfubp1-Ser1258Pro	0/53	1/88	2/450	0/4	4/133	1/82	0/141	0/81	2/213	0/28	0/219	0/39	0/9	10/1540

	0.0%	1.1%	0.4%	0.0%	3.0%	1.2%	0.0%	0.0%	0.9%	0.0%	0.0%	0.0%	0.0%	0.6%
	0.0%-0.0%	0.0%-3.0%	0.0%-1.0%	0.0%-0.0%	0.6%-5.4%	0.0%-3.2%	0.0%-0.0%	0.0%-0.0%	0.0%-2.0%	0.0%-0.0%	0.0%-0.0%	0.0%-0.0%	0.0%-0.0%	0.3%-1.0%
Pfubp1-Ser1258Thr	0/53	6/88	3/450	0/4	6/133	1/82	3/141	0/81	2/213	0/28	2/219	2/39	0/9	25/1540
	0.0%	6.8%	0.7%	0.0%	4.5%	1.2%	2.1%	0.0%	0.9%	0.0%	0.9%	5.1%	0.0%	1.6%
	0.0%-0.0%	2.4%-11.2%	0.0%-1.3%	0.0%-0.0%	1.6%-7.5%	0.0%-3.2%	0.1%-4.1%	0.0%-0.0%	0.0%-2.0%	0.0%-0.0%	0.0%-2.0%	0.0%-10.9%	0.0%-0.0%	1.1%-2.2%
Pfubp1-Ser1472Tyr	0/86	0/102	1/454	0/9	0/136	0/107	1/134	0/98	0/196	0/31	1/229	0/67	0/12	3/1661
	0.0%	0.0%	0.2%	0.0%	0.0%	0.0%	0.7%	0.0%	0.0%	0.0%	0.4%	0.0%	0.0%	0.2%
	0.0%-0.0%	0.0%-0.0%	0.0%-0.6%	0.0%-0.0%	0.0%-0.0%	0.0%-0.0%	0.0%-2.0%	0.0%-0.0%	0.0%-0.0%	0.0%-0.0%	0.0%-1.2%	0.0%-0.0%	0.0%-0.0%	0.0%-0.4%
Pfubp1-Ser161Tyr	0/192	0/194	3/561	0/18	0/181	1/207	0/229	0/185	0/294	0/41	0/352	0/211	0/40	4/2705
	0.0%	0.0%	0.5%	0.0%	0.0%	0.5%	0.0%	0.0%	0.0%	0.0%	0.0%	0.0%	0.0%	0.1%
	0.0%-0.0%	0.0%-0.0%	0.0%-1.0%	0.0%-0.0%	0.0%-0.0%	0.0%-1.3%	0.0%-0.0%	0.0%-0.0%	0.0%-0.0%	0.0%-0.0%	0.0%-0.0%	0.0%-0.0%	0.0%-0.0%	0.0%-0.3%
Pfubp1-Ser2281Pro	1/92	0/124	0/489	0/13	0/153	0/120	1/170	0/112	0/245	1/41	1/241	0/61	0/29	4/1890
	1.1%	0.0%	0.0%	0.0%	0.0%	0.0%	0.6%	0.0%	0.0%	2.4%	0.4%	0.0%	0.0%	0.2%
	0.0%-2.9%	0.0%-0.0%	0.0%-0.0%	0.0%-0.0%	0.0%-0.0%	0.0%-0.0%	0.0%-1.6%	0.0%-0.0%	0.0%-0.0%	0.0%-6.4%	0.0%-1.1%	0.0%-0.0%	0.0%-0.0%	0.0%-0.4%
Pfubp1-Ser2635Cys	0/5	0/70	2/284	0/8	0/128	0/56	0/129	0/14	0/157	0/28	3/109	0/23	0/13	5/1024
	0.0%	0.0%	0.7%	0.0%	0.0%	0.0%	0.0%	0.0%	0.0%	0.0%	2.8%	0.0%	0.0%	0.5%
	0.0%-0.0%	0.0%-0.0%	0.0%-1.5%	0.0%-0.0%	0.0%-0.0%	0.0%-0.0%	0.0%-0.0%	0.0%-0.0%	0.0%-0.0%	0.0%-0.0%	0.2%-5.3%	0.0%-0.0%	0.0%-0.0%	0.1%-0.8%
Pfubp1-Ser2729Pro	0/36	0/76	5/319	0/16	0/142	0/67	0/139	0/58	0/169	0/27	0/144	0/26	0/27	5/1246
	0.0%	0.0%	1.6%	0.0%	0.0%	0.0%	0.0%	0.0%	0.0%	0.0%	0.0%	0.0%	0.0%	0.4%
	0.0%-0.0%	0.0%-0.0%	0.4%-2.7%	0.0%-0.0%	0.0%-0.0%	0.0%-0.0%	0.0%-0.0%	0.0%-0.0%	0.0%-0.0%	0.0%-0.0%	0.0%-0.0%	0.0%-0.0%	0.0%-0.0%	0.1%-0.7%
Pfubp1-Ser281Ala	15/19	40/58	180/254	3/3	65/109	27/48	93/117	7/22	74/137	19/31	48/100	10/12	5/5	586/915
	78.9%	69.0%	70.9%	100.0%	59.6%	56.2%	79.5%	31.8%	54.0%	61.3%	48.0%	83.3%	100.0%	64.0%
	63.6%-94.3%	59.0%-79.0%	66.2%-75.6%	100.0%-100.0%	51.9%-67.4%	44.5%-68.0%	73.3%-85.6%	15.5%-48.2%	47.0%-61.0%	46.9%-75.7%	39.8%-56.2%	65.6%-100.0%	00.0%-100.0%	61.4%-66.7%
Pfubp1-Ser281Thr	1/19	0/58	3/254	0/3	0/109	0/48	0/117	1/22	2/137	0/31	0/100	0/12	0/5	7/915
	5.3%	0.0%	1.2%	0.0%	0.0%	0.0%	0.0%	4.5%	1.5%	0.0%	0.0%	0.0%	0.0%	0.8%
	0.0%-13.7%	0.0%-0.0%	0.1%-2.3%	0.0%-0.0%	0.0%-0.0%	0.0%-0.0%	0.0%-0.0%	0.0%-11.9%	0.0%-3.1%	0.0%-0.0%	0.0%-0.0%	0.0%-0.0%	0.0%-0.0%	0.3%-1.2%
Pfubp1-Ser286Arg	0/19	0/58	2/254	0/3	0/109	0/48	1/118	0/22	0/137	0/31	0/101	0/12	0/5	3/917
	0.0%	0.0%	0.8%	0.0%	0.0%	0.0%	0.8%	0.0%	0.0%	0.0%	0.0%	0.0%	0.0%	0.3%
	0.0%-0.0%	0.0%-0.0%	0.0%-1.7%	0.0%-0.0%	0.0%-0.0%	0.0%-0.0%	0.0%-2.2%	0.0%-0.0%	0.0%-0.0%	0.0%-0.0%	0.0%-0.0%	0.0%-0.0%	0.0%-0.0%	0.0%-0.6%
Pfubp1-Ser286Ile	1/19	0/58	3/254	0/3	0/109	0/48	1/118	0/22	1/137	0/31	0/101	0/12	0/5	6/917
	5.3%	0.0%	1.2%	0.0%	0.0%	0.0%	0.8%	0.0%	0.7%	0.0%	0.0%	0.0%	0.0%	0.7%
	0.0%-13.7%	0.0%-0.0%	0.1%-2.3%	0.0%-0.0%	0.0%-0.0%	0.0%-0.0%	0.0%-2.2%	0.0%-0.0%	0.0%-1.9%	0.0%-0.0%	0.0%-0.0%	0.0%-0.0%	0.0%-0.0%	0.2%-1.1%
Pfubp1-Ser2946Cys	14/170	1/165	9/575	0/24	4/189	1/176	1/224	5/188	10/299	0/52	3/327	2/162	0/44	50/2595
	8.2%	0.6%	1.6%	0.0%	2.1%	0.6%	0.4%	2.7%	3.3%	0.0%	0.9%	1.2%	0.0%	1.9%
	4.8%-11.7%	0.0%-1.6%	0.7%-2.4%	0.0%-0.0%	0.4%-3.8%	0.0%-1.5%	0.0%-1.2%	0.7%-4.6%	1.6%-5.1%	0.0%-0.0%	0.1%-1.8%	0.0%-2.7%	0.0%-0.0%	1.5%-2.4%
Pfubp1-Ser3121Asn	8/140	8/148	33/484	0/3	11/146	12/167	14/175	13/152	30/261	0/27	6/238	10/187	0/14	145/2142
	5.7%	5.4%	6.8%	0.0%	7.5%	7.2%	8.0%	8.6%	11.5%	0.0%	2.5%	5.3%	0.0%	6.8%
	2.5%-8.9%	2.3%-8.5%	4.9%-8.7%	0.0%-0.0%	3.9%-11.1%	3.9%-10.5%	4.6%-11.4%	4.8%-12.3%	8.2%-14.7%	0.0%-0.0%	0.8%-4.2%	2.6%-8.1%	0.0%-0.0%	5.9%-7.7%
Pfubp1-Ser389Asn	no coverage	3/3	3/5	no coverage	1/2	0/2	0/1	0/3	2/4	no coverage	0/3	3/4	0/1	12/28
	no coverage	100.0%	60.0%	no coverage	50.0%	0.0%	0.0%	0.0%	50.0%	no coverage	0.0%	75.0%	0.0%	42.9%
	no coverage	100.0%-100.0%	24.0%-96.0%	no coverage	0.0%-100.0%	0.0%-0.0%	0.0%-0.0%	0.0%-0.0%	8.9%-91.1%	no coverage	0.0%-0.0%	39.4%-100.0%	0.0%-0.0%	27.5%-58.2%
Pfubp1-Ser806Arg	0/187	0/177	0/503	0/15	0/145	0/189	1/208	3/215	0/247	0/30	1/300	2/269	0/18	7/2503
	0.0%	0.0%	0.0%	0.0%	0.0%	0.0%	0.5%	1.4%	0.0%	0.0%	0.3%	0.7%	0.0%	0.3%
	0.0%-0.0%	0.0%-0.0%	0.0%-0.0%	0.0%-0.0%	0.0%-0.0%	0.0%-0.0%	0.0%-1.3%	0.1%-2.7%	0.0%-0.0%	0.0%-0.0%	0.0%-0.9%	0.0%-1.6%	0.0%-0.0%	0.1%-0.5%

Pfubp1-Ser806Asn	0/187	0/177	1/503	0/15	0/145	0/189	0/208	0/215	3/247	0/30	2/300	0/269	0/18	6/2503
	0.0%	0.0%	0.2%	0.0%	0.0%	0.0%	0.0%	0.0%	1.2%	0.0%	0.7%	0.0%	0.0%	0.2%
	0.0%-0.0%	0.0%-0.0%	0.0%-0.5%	0.0%-0.0%	0.0%-0.0%	0.0%-0.0%	0.0%-0.0%	0.0%-0.0%	0.1%-2.4%	0.0%-0.0%	0.0%-1.4%	0.0%-0.0%	0.0%-0.0%	0.1%-0.4%
Pfubp1-Thr1500Ala	0/154	0/166	0/509	0/14	1/171	1/178	0/192	1/157	0/244	0/35	0/255	0/167	0/28	3/2270
	0.0%	0.0%	0.0%	0.0%	0.6%	0.6%	0.0%	0.6%	0.0%	0.0%	0.0%	0.0%	0.0%	0.1%
	0.0%-0.0%	0.0%-0.0%	0.0%-0.0%	0.0%-0.0%	0.0%-1.5%	0.0%-1.5%	0.0%-0.0%	0.0%-1.7%	0.0%-0.0%	0.0%-0.0%	0.0%-0.0%	0.0%-0.0%	0.0%-0.0%	0.0%-0.3%
Pfubp1-Thr160Lys	0/192	0/194	7/561	0/18	0/181	0/207	6/229	0/185	1/294	0/41	0/352	0/211	0/40	14/2705
	0.0%	0.0%	1.2%	0.0%	0.0%	0.0%	2.6%	0.0%	0.3%	0.0%	0.0%	0.0%	0.0%	0.5%
	0.0%-0.0%	0.0%-0.0%	0.5%-2.0%	0.0%-0.0%	0.0%-0.0%	0.0%-0.0%	0.9%-4.4%	0.0%-0.0%	0.0%-0.9%	0.0%-0.0%	0.0%-0.0%	0.0%-0.0%	0.0%-0.0%	0.3%-0.7%
Pfubp1-Thr1612Ala	0/3	0/65	2/222	0/1	0/111	0/51	0/115	0/29	0/80	0/10	0/61	0/18	1/9	3/775
	0.0%	0.0%	0.9%	0.0%	0.0%	0.0%	0.0%	0.0%	0.0%	0.0%	0.0%	0.0%	11.1%	0.4%
	0.0%-0.0%	0.0%-0.0%	0.0%-1.9%	0.0%-0.0%	0.0%-0.0%	0.0%-0.0%	0.0%-0.0%	0.0%-0.0%	0.0%-0.0%	0.0%-0.0%	0.0%-0.0%	0.0%-0.0%	0.0%-28.3%	0.0%-0.8%
Pfubp1-Thr1675Ala	0/114	0/122	7/488	0/10	0/163	0/130	0/169	0/109	0/233	0/35	0/277	0/114	0/22	7/1986
	0.0%	0.0%	1.4%	0.0%	0.0%	0.0%	0.0%	0.0%	0.0%	0.0%	0.0%	0.0%	0.0%	0.4%
	0.0%-0.0%	0.0%-0.0%	0.5%-2.3%	0.0%-0.0%	0.0%-0.0%	0.0%-0.0%	0.0%-0.0%	0.0%-0.0%	0.0%-0.0%	0.0%-0.0%	0.0%-0.0%	0.0%-0.0%	0.0%-0.0%	0.1%-0.6%
Pfubp1-Thr2369Ala	0/19	0/77	0/331	0/7	0/146	0/68	0/137	0/28	3/196	0/37	0/153	0/22	0/13	3/1234
	0.0%	0.0%	0.0%	0.0%	0.0%	0.0%	0.0%	0.0%	1.5%	0.0%	0.0%	0.0%	0.0%	0.2%
	0.0%-0.0%	0.0%-0.0%	0.0%-0.0%	0.0%-0.0%	0.0%-0.0%	0.0%-0.0%	0.0%-0.0%	0.0%-0.0%	0.1%-3.0%	0.0%-0.0%	0.0%-0.0%	0.0%-0.0%	0.0%-0.0%	0.0%-0.5%
Pfubp1-Thr275Lys	7/19	0/58	11/254	0/3	1/109	0/48	2/117	8/22	13/137	2/31	2/100	0/12	0/5	46/915
	36.8%	0.0%	4.3%	0.0%	0.9%	0.0%	1.7%	36.4%	9.5%	6.5%	2.0%	0.0%	0.0%	5.0%
	18.6%-55.0%	0.0%-0.0%	2.2%-6.4%	0.0%-0.0%	0.0%-2.4%	0.0%-0.0%	0.0%-3.7%	19.5%-53.2%	5.4%-13.6%	0.0%-13.7%	0.0%-4.3%	0.0%-0.0%	0.0%-0.0%	3.8%-6.2%
Pfubp1-Thr278Lys	1/19	0/58	3/254	0/3	0/109	0/48	0/117	2/22	5/137	0/31	0/100	0/12	0/5	11/915
	5.3%	0.0%	1.2%	0.0%	0.0%	0.0%	0.0%	9.1%	3.6%	0.0%	0.0%	0.0%	0.0%	1.2%
	0.0%-13.7%	0.0%-0.0%	0.1%-2.3%	0.0%-0.0%	0.0%-0.0%	0.0%-0.0%	0.0%-0.0%	0.0%-19.2%	1.0%-6.3%	0.0%-0.0%	0.0%-0.0%	0.0%-0.0%	0.0%-0.0%	0.6%-1.8%
Pfubp1-Thr3018Lys	0/150	0/186	0/576	0/21	0/186	0/178	0/215	0/148	3/292	0/46	0/345	0/162	0/35	3/2540
	0.0%	0.0%	0.0%	0.0%	0.0%	0.0%	0.0%	0.0%	1.0%	0.0%	0.0%	0.0%	0.0%	0.1%
	0.0%-0.0%	0.0%-0.0%	0.0%-0.0%	0.0%-0.0%	0.0%-0.0%	0.0%-0.0%	0.0%-0.0%	0.0%-0.0%	0.1%-2.0%	0.0%-0.0%	0.0%-0.0%	0.0%-0.0%	0.0%-0.0%	0.0%-0.2%
Pfubp1-Thr3212Met	0/48	0/90	7/435	0/15	0/161	0/92	0/167	0/75	0/242	1/36	0/236	0/45	0/37	8/1679
	0.0%	0.0%	1.6%	0.0%	0.0%	0.0%	0.0%	0.0%	0.0%	2.8%	0.0%	0.0%	0.0%	0.5%
	0.0%-0.0%	0.0%-0.0%	0.6%-2.6%	0.0%-0.0%	0.0%-0.0%	0.0%-0.0%	0.0%-0.0%	0.0%-0.0%	0.0%-0.0%	0.0%-7.3%	0.0%-0.0%	0.0%-0.0%	0.0%-0.0%	0.2%-0.8%
Pfubp1-Thr444Lys	no coverage	0/54	0/202	no coverage	0/90	0/32	3/103	0/3	1/76	1/18	0/64	0/13	0/1	5/656
	no coverage	0.0%	0.0%	no coverage	0.0%	0.0%	2.9%	0.0%	1.3%	5.6%	0.0%	0.0%	0.0%	0.8%
	no coverage	0.0%-0.0%	0.0%-0.0%	no coverage	0.0%-0.0%	0.0%-0.0%	0.2%-5.6%	0.0%-0.0%	0.0%-3.5%	0.0%-14.4%	0.0%-0.0%	0.0%-0.0%	0.0%-0.0%	0.2%-1.3%
Pfubp1-Thr870Lys	0/34	0/70	3/323	0/3	0/130	0/56	0/121	0/68	0/161	0/26	0/159	0/24	0/11	3/1186
	0.0%	0.0%	0.9%	0.0%	0.0%	0.0%	0.0%	0.0%	0.0%	0.0%	0.0%	0.0%	0.0%	0.3%
	0.0%-0.0%	0.0%-0.0%	0.1%-1.8%	0.0%-0.0%	0.0%-0.0%	0.0%-0.0%	0.0%-0.0%	0.0%-0.0%	0.0%-0.0%	0.0%-0.0%	0.0%-0.0%	0.0%-0.0%	0.0%-0.0%	0.0%-0.5%
Pfubp1-Tyr1462His	1/86	0/102	13/455	0/9	0/136	3/107	3/134	0/98	3/196	0/31	5/229	1/67	0/12	29/1662
	1.2%	0.0%	2.9%	0.0%	0.0%	2.8%	2.2%	0.0%	1.5%	0.0%	2.2%	1.5%	0.0%	1.7%
	0.0%-3.1%	0.0%-0.0%	1.6%-4.1%	0.0%-0.0%	0.0%-0.0%	0.2%-5.4%	0.1%-4.3%	0.0%-0.0%	0.1%-3.0%	0.0%-0.0%	0.6%-3.8%	0.0%-3.9%	0.0%-0.0%	1.2%-2.3%
Pfubp1-Tyr1549Cys	0/44	0/84	0/297	0/6	0/114	0/63	0/116	0/70	0/121	2/18	1/138	0/29	0/7	3/1107
	0.0%	0.0%	0.0%	0.0%	0.0%	0.0%	0.0%	0.0%	0.0%	11.1%	0.7%	0.0%	0.0%	0.3%
	0.0%-0.0%	0.0%-0.0%	0.0%-0.0%	0.0%-0.0%	0.0%-0.0%	0.0%-0.0%	0.0%-0.0%	0.0%-0.0%	0.0%-0.0%	0.0%-23.3%	0.0%-1.9%	0.0%-0.0%	0.0%-0.0%	0.0%-0.5%
Pfubp1-Tyr1556Cys	0/46	0/90	6/336	0/8	1/133	1/72	2/131	1/77	0/140	0/21	1/168	0/34	0/14	12/1270
	0.0%	0.0%	1.8%	0.0%	0.8%	1.4%	1.5%	1.3%	0.0%	0.0%	0.6%	0.0%	0.0%	0.9%

	0.0%-0.0%	0.0%-0.0%	0.6%-3.0%	0.0%-0.0%	0.0%-2.0%	0.0%-3.7%	0.0%-3.3%	0.0%-3.4%	0.0%-0.0%	0.0%-0.0%	0.0%-1.6%	0.0%-0.0%	0.0%-0.0%	0.5%-1.4%
Pfubp1-Tyr1896Asp	0/243	2/273	0/622	0/34	0/227	0/277	0/337	0/291	1/330	0/59	0/390	0/388	0/63	3/3534
	0.0%	0.7%	0.0%	0.0%	0.0%	0.0%	0.0%	0.0%	0.3%	0.0%	0.0%	0.0%	0.0%	0.1%
	0.0%-0.0%	0.0%-1.6%	0.0%-0.0%	0.0%-0.0%	0.0%-0.0%	0.0%-0.0%	0.0%-0.0%	0.0%-0.0%	0.0%-0.8%	0.0%-0.0%	0.0%-0.0%	0.0%-0.0%	0.0%-0.0%	0.0%-0.2%
Pfubp1-Tyr1969Asp	0/88	1/112	2/472	0/8	0/87	0/102	0/144	0/92	0/212	0/29	0/241	1/86	0/11	4/1684
	0.0%	0.9%	0.4%	0.0%	0.0%	0.0%	0.0%	0.0%	0.0%	0.0%	0.0%	1.2%	0.0%	0.2%
	0.0%-0.0%	0.0%-2.4%	0.0%-0.9%	0.0%-0.0%	0.0%-0.0%	0.0%-0.0%	0.0%-0.0%	0.0%-0.0%	0.0%-0.0%	0.0%-0.0%	0.0%-0.0%	0.0%-3.1%	0.0%-0.0%	0.0%-0.4%
Pfubp1-Tyr279Asn	1/19	0/58	1/254	0/3	0/109	0/48	0/117	0/22	1/137	0/31	1/100	0/12	0/5	4/915
	5.3%	0.0%	0.4%	0.0%	0.0%	0.0%	0.0%	0.0%	0.7%	0.0%	1.0%	0.0%	0.0%	0.4%
	0.0%-13.7%	0.0%-0.0%	0.0%-1.0%	0.0%-0.0%	0.0%-0.0%	0.0%-0.0%	0.0%-0.0%	0.0%-0.0%	0.0%-1.9%	0.0%-0.0%	0.0%-2.6%	0.0%-0.0%	0.0%-0.0%	0.1%-0.8%
Pfubp1-Tyr279His	0/19	0/58	0/254	0/3	0/109	0/48	4/117	0/22	3/137	0/31	0/100	0/12	0/5	7/915
	0.0%	0.0%	0.0%	0.0%	0.0%	0.0%	3.4%	0.0%	2.2%	0.0%	0.0%	0.0%	0.0%	0.8%
	0.0%-0.0%	0.0%-0.0%	0.0%-0.0%	0.0%-0.0%	0.0%-0.0%	0.0%-0.0%	0.7%-6.2%	0.0%-0.0%	0.1%-4.2%	0.0%-0.0%	0.0%-0.0%	0.0%-0.0%	0.0%-0.0%	0.3%-1.2%
Pfubp1-Tyr279Ser	0/19	1/58	2/254	0/3	1/109	0/48	0/117	0/22	2/137	0/31	1/100	0/12	0/5	7/915
	0.0%	1.7%	0.8%	0.0%	0.9%	0.0%	0.0%	0.0%	1.5%	0.0%	1.0%	0.0%	0.0%	0.8%
	0.0%-0.0%	0.0%-4.5%	0.0%-1.7%	0.0%-0.0%	0.0%-2.4%	0.0%-0.0%	0.0%-0.0%	0.0%-0.0%	0.0%-3.1%	0.0%-0.0%	0.0%-2.6%	0.0%-0.0%	0.0%-0.0%	0.3%-1.2%
Pfubp1-Tyr2822Cys	7/167	4/165	33/558	3/19	7/179	7/183	16/215	8/169	6/271	3/49	11/285	5/181	2/38	112/2479
	4.2%	2.4%	5.9%	15.8%	3.9%	3.8%	7.4%	4.7%	2.2%	6.1%	3.9%	2.8%	5.3%	4.5%
	1.6%-6.7%	0.5%-4.4%	4.3%-7.6%	2.0%-29.5%	1.5%-6.3%	1.5%-6.2%	4.5%-10.4%	2.0%-7.4%	0.7%-3.7%	0.5%-11.8%	2.0%-5.7%	0.8%-4.8%	0.0%-11.2%	3.8%-5.2%
Pfubp1-Tyr3045Cys	0/150	1/186	1/576	0/21	0/186	0/178	0/215	0/148	0/292	0/46	0/345	1/162	0/35	3/2540
	0.0%	0.5%	0.2%	0.0%	0.0%	0.0%	0.0%	0.0%	0.0%	0.0%	0.0%	0.6%	0.0%	0.1%
	0.0%-0.0%	0.0%-1.4%	0.0%-0.5%	0.0%-0.0%	0.0%-0.0%	0.0%-0.0%	0.0%-0.0%	0.0%-0.0%	0.0%-0.0%	0.0%-0.0%	0.0%-0.0%	0.0%-1.6%	0.0%-0.0%	0.0%-0.2%
Pfubp1-Tyr615His	4/4	63/63	230/230	no coverage	101/101	47/47	104/104	24/24	125/127	16/16	92/92	17/17	4/4	827/829
	100.0%	100.0%	100.0%	no coverage	100.0%	100.0%	100.0%	100.0%	98.4%	100.0%	100.0%	100.0%	100.0%	99.8%
	100.0%-100.0%	100.0%-100.0%	100.0%-100.0%	no coverage	100.0%-100.0%	100.0%-100.0%	100.0%-100.0%	100.0%-100.0%	96.6%-100.0%	100.0%-100.0%	100.0%-100.0%	100.0%-100.0%	100.0%-100.0%	99.5%-100.0%
Pfubp1-Val1285Met	0/60	1/76	0/362	0/8	0/128	0/65	0/130	0/85	6/155	0/27	0/172	0/43	0/12	7/1323
	0.0%	1.3%	0.0%	0.0%	0.0%	0.0%	0.0%	0.0%	3.9%	0.0%	0.0%	0.0%	0.0%	0.5%
	0.0%-0.0%	0.0%-3.5%	0.0%-0.0%	0.0%-0.0%	0.0%-0.0%	0.0%-0.0%	0.0%-0.0%	0.0%-0.0%	1.3%-6.4%	0.0%-0.0%	0.0%-0.0%	0.0%-0.0%	0.0%-0.0%	0.2%-0.9%
Pfubp1-Val3063Glu	0/132	0/172	1/547	0/14	0/169	0/158	1/194	0/136	0/273	0/38	1/331	0/146	0/25	3/2335
	0.0%	0.0%	0.2%	0.0%	0.0%	0.0%	0.5%	0.0%	0.0%	0.0%	0.3%	0.0%	0.0%	0.1%
	0.0%-0.0%	0.0%-0.0%	0.0%-0.5%	0.0%-0.0%	0.0%-0.0%	0.0%-0.0%	0.0%-1.4%	0.0%-0.0%	0.0%-0.0%	0.0%-0.0%	0.0%-0.8%	0.0%-0.0%	0.0%-0.0%	0.0%-0.3%
Pfubp1-Val3072Ala	0/126	0/162	1/537	0/13	4/163	2/159	0/191	0/134	0/269	0/40	0/320	0/143	0/25	7/2282
	0.0%	0.0%	0.2%	0.0%	2.5%	1.3%	0.0%	0.0%	0.0%	0.0%	0.0%	0.0%	0.0%	0.3%
	0.0%-0.0%	0.0%-0.0%	0.0%-0.5%	0.0%-0.0%	0.5%-4.4%	0.0%-2.7%	0.0%-0.0%	0.0%-0.0%	0.0%-0.0%	0.0%-0.0%	0.0%-0.0%	0.0%-0.0%	0.0%-0.0%	0.1%-0.5%
Pfubp1-Val3120Met	0/138	0/148	2/480	0/3	0/145	1/166	0/168	0/147	0/256	0/26	1/238	0/182	0/14	4/2111
	0.0%	0.0%	0.4%	0.0%	0.0%	0.6%	0.0%	0.0%	0.0%	0.0%	0.4%	0.0%	0.0%	0.2%
	0.0%-0.0%	0.0%-0.0%	0.0%-0.9%	0.0%-0.0%	0.0%-0.0%	0.0%-1.6%	0.0%-0.0%	0.0%-0.0%	0.0%-0.0%	0.0%-0.0%	0.0%-1.1%	0.0%-0.0%	0.0%-0.0%	0.0%-0.3%
Pfubp1-Val53Ile	0/29	0/51	0/295	no coverage	0/82	0/35	0/111	1/60	2/125	0/22	0/125	0/14	0/2	3/951
	0.0%	0.0%	0.0%	no coverage	0.0%	0.0%	0.0%	1.7%	1.6%	0.0%	0.0%	0.0%	0.0%	0.3%
	0.0%-0.0%	0.0%-0.0%	0.0%-0.0%	no coverage	0.0%-0.0%	0.0%-0.0%	0.0%-0.0%	0.0%-4.4%	0.0%-3.4%	0.0%-0.0%	0.0%-0.0%	0.0%-0.0%	0.0%-0.0%	0.0%-0.6%
Pfubp1-Val627Ile	0/4	1/63	2/230	no coverage	0/101	0/47	0/104	1/24	1/127	0/16	1/92	2/17	0/4	8/829
	0.0%	1.6%	0.9%	no coverage	0.0%	0.0%	0.0%	4.2%	0.8%	0.0%	1.1%	11.8%	0.0%	1.0%
	0.0%-0.0%	0.0%-4.2%	0.0%-1.9%	no coverage	0.0%-0.0%	0.0%-0.0%	0.0%-0.0%	0.0%-10.9%	0.0%-2.1%	0.0%-0.0%	0.0%-2.9%	0.0%-24.6%	0.0%-0.0%	0.4%-1.5%
Pfubp1-Val708Ile	0/52	0/83	1/397	0/12	0/142	0/77	1/141	3/64	0/202	0/31	1/186	0/33	0/20	6/1440

	0.0%	0.0%	0.3%	0.0%	0.0%	0.0%	0.7%	4.7%	0.0%	0.0%	0.5%	0.0%	0.0%	0.4%
	0.0%-0.0%	0.0%-0.0%	0.0%-0.7%	0.0%-0.0%	0.0%-0.0%	0.0%-0.0%	0.0%-1.9%	0.3%-9.0%	0.0%-0.0%	0.0%-0.0%	0.0%-1.4%	0.0%-0.0%	0.0%-0.0%	0.1%-0.7%
Pfubp1-Val739Met	0/180	0/197	0/566	0/27	0/202	0/190	0/252	0/216	2/301	0/55	1/348	0/248	0/44	3/2826
	0.0%	0.0%	0.0%	0.0%	0.0%	0.0%	0.0%	0.0%	0.7%	0.0%	0.3%	0.0%	0.0%	0.1%
	0.0%-0.0%	0.0%-0.0%	0.0%-0.0%	0.0%-0.0%	0.0%-0.0%	0.0%-0.0%	0.0%-0.0%	0.0%-0.0%	0.0%-1.4%	0.0%-0.0%	0.0%-0.8%	0.0%-0.0%	0.0%-0.0%	0.0%-0.2%
Sec14-Asn615Asp	13/59	4/94	62/422	1/15	20/163	8/89	21/170	9/85	13/228	4/45	47/214	11/46	6/39	219/1669
	22.0%	4.3%	14.7%	6.7%	12.3%	9.0%	12.4%	10.6%	5.7%	8.9%	22.0%	23.9%	15.4%	13.1%
	13.2%-30.9%	0.8%-7.7%	11.9%-17.5%	0.0%-17.3%	8.0%-16.5%	4.0%-14.0%	8.2%-16.5%	5.1%-16.1%	3.2%-8.2%	1.9%-15.9%	17.3%-26.6%	13.6%-34.3%	5.9%-24.9%	11.8%-14.5%
Sec14-Cys618Ser	3/59	0/94	0/422	0/15	0/163	0/89	0/170	0/85	0/228	0/45	0/214	0/46	0/39	3/1669
	5.1%	0.0%	0.0%	0.0%	0.0%	0.0%	0.0%	0.0%	0.0%	0.0%	0.0%	0.0%	0.0%	0.2%
	0.4%-9.8%	0.0%-0.0%	0.0%-0.0%	0.0%-0.0%	0.0%-0.0%	0.0%-0.0%	0.0%-0.0%	0.0%-0.0%	0.0%-0.0%	0.0%-0.0%	0.0%-0.0%	0.0%-0.0%	0.0%-0.0%	0.0%-0.4%
Sec14-His638Gln	0/59	0/94	3/422	0/15	0/163	0/89	0/170	0/85	0/228	0/45	0/214	0/46	0/39	3/1669
	0.0%	0.0%	0.7%	0.0%	0.0%	0.0%	0.0%	0.0%	0.0%	0.0%	0.0%	0.0%	0.0%	0.2%
	0.0%-0.0%	0.0%-0.0%	0.0%-1.4%	0.0%-0.0%	0.0%-0.0%	0.0%-0.0%	0.0%-0.0%	0.0%-0.0%	0.0%-0.0%	0.0%-0.0%	0.0%-0.0%	0.0%-0.0%	0.0%-0.0%	0.0%-0.4%
crt-Ala220Ser	0/8	1/56	16/264	4/30	2/119	1/38	5/384	0/23	4/98	0/25	1/71	3/223	0/26	37/1365
	0.0%	1.8%	6.1%	13.3%	1.7%	2.6%	1.3%	0.0%	4.1%	0.0%	1.4%	1.3%	0.0%	2.7%
	0.0%-0.0%	0.0%-4.7%	3.6%-8.5%	3.1%-23.5%	0.0%-3.6%	0.0%-6.9%	0.4%-2.3%	0.0%-0.0%	0.8%-7.4%	0.0%-0.0%	0.0%-3.7%	0.1%-2.6%	0.0%-0.0%	2.0%-3.4%
crt-Ala355Thr	0/30	0/69	3/390	0/49	0/132	0/63	1/471	0/47	0/150	0/25	0/133	0/278	0/16	4/1853
	0.0%	0.0%	0.8%	0.0%	0.0%	0.0%	0.2%	0.0%	0.0%	0.0%	0.0%	0.0%	0.0%	0.2%
	0.0%-0.0%	0.0%-0.0%	0.0%-1.5%	0.0%-0.0%	0.0%-0.0%	0.0%-0.0%	0.0%-0.6%	0.0%-0.0%	0.0%-0.0%	0.0%-0.0%	0.0%-0.0%	0.0%-0.0%	0.0%-0.0%	0.0%-0.4%
crt-Arg371Ile	1/6	4/67	25/356	4/38	4/136	0/57	9/446	0/12	5/130	1/25	1/94	4/259	0/15	58/1641
	16.7%	6.0%	7.0%	10.5%	2.9%	0.0%	2.0%	0.0%	3.8%	4.0%	1.1%	1.5%	0.0%	3.5%
	0.0%-41.7%	1.2%-10.7%	4.8%-9.3%	2.3%-18.7%	0.6%-5.3%	0.0%-0.0%	0.9%-3.1%	0.0%-0.0%	1.1%-6.6%	0.0%-10.4%	0.0%-2.8%	0.3%-2.8%	0.0%-0.0%	2.8%-4.3%
crt-Asn326Ser	0/9	0/81	0/404	0/58	2/157	0/69	0/483	0/25	4/169	0/33	0/137	1/293	0/22	7/1940
	0.0%	0.0%	0.0%	0.0%	1.3%	0.0%	0.0%	0.0%	2.4%	0.0%	0.0%	0.3%	0.0%	0.4%
	0.0%-0.0%	0.0%-0.0%	0.0%-0.0%	0.0%-0.0%	0.0%-2.7%	0.0%-0.0%	0.0%-0.0%	0.0%-0.0%	0.4%-4.3%	0.0%-0.0%	0.0%-0.0%	0.0%-0.9%	0.0%-0.0%	0.1%-0.6%
crt-Asn75Glu	0/5	1/71	27/406	6/52	3/140	0/62	2/485	0/19	6/177	0/30	0/134	2/292	0/18	47/1891
	0.0%	1.4%	6.7%	11.5%	2.1%	0.0%	0.4%	0.0%	3.4%	0.0%	0.0%	0.7%	0.0%	2.5%
	0.0%-0.0%	0.0%-3.7%	4.6%-8.7%	4.3%-18.8%	0.1%-4.2%	0.0%-0.0%	0.0%-0.9%	0.0%-0.0%	1.2%-5.6%	0.0%-0.0%	0.0%-0.0%	0.0%-1.5%	0.0%-0.0%	1.9%-3.1%
crt-Asp24Tyr	1/31	7/80	52/391	2/40	10/154	12/75	56/437	8/66	9/182	0/35	11/141	22/245	4/33	194/1910
	3.2%	8.8%	13.3%	5.0%	6.5%	16.0%	12.8%	12.1%	4.9%	0.0%	7.8%	9.0%	12.1%	10.2%
	0.0%-8.4%	3.6%-13.9%	10.5%-16.1%	0.0%-10.7%	3.2%-9.8%	9.0%-23.0%	10.2%-15.4%	5.5%-18.7%	2.3%-7.6%	0.0%-0.0%	4.1%-11.5%	6.0%-12.0%	2.8%-21.5%	9.0%-11.3%
crt-Gln271Glu	0/10	1/51	16/279	4/31	4/111	0/42	4/410	0/26	4/101	0/25	0/61	4/237	0/7	37/1391
	0.0%	2.0%	5.7%	12.9%	3.6%	0.0%	1.0%	0.0%	4.0%	0.0%	0.0%	1.7%	0.0%	2.7%
	0.0%-0.0%	0.0%-5.2%	3.4%-8.0%	3.0%-22.8%	0.7%-6.5%	0.0%-0.0%	0.2%-1.8%	0.0%-0.0%	0.8%-7.2%	0.0%-0.0%	0.0%-0.0%	0.3%-3.1%	0.0%-0.0%	2.0%-3.4%
crt-Lys76Thr	0/5	1/71	27/406	6/52	3/140	0/62	2/485	0/19	6/177	0/30	0/134	2/292	0/18	47/1891
	0.0%	1.4%	6.7%	11.5%	2.1%	0.0%	0.4%	0.0%	3.4%	0.0%	0.0%	0.7%	0.0%	2.5%
	0.0%-0.0%	0.0%-3.7%	4.6%-8.7%	4.3%-18.8%	0.1%-4.2%	0.0%-0.0%	0.0%-0.9%	0.0%-0.0%	1.2%-5.6%	0.0%-0.0%	0.0%-0.0%	0.0%-1.5%	0.0%-0.0%	1.9%-3.1%
crt-Met74Ile	0/5	1/71	27/406	6/52	3/140	0/62	2/485	0/19	6/177	0/30	0/134	2/292	0/18	47/1891
	0.0%	1.4%	6.7%	11.5%	2.1%	0.0%	0.4%	0.0%	3.4%	0.0%	0.0%	0.7%	0.0%	2.5%
	0.0%-0.0%	0.0%-3.7%	4.6%-8.7%	4.3%-18.8%	0.1%-4.2%	0.0%-0.0%	0.0%-0.9%	0.0%-0.0%	1.2%-5.6%	0.0%-0.0%	0.0%-0.0%	0.0%-1.5%	0.0%-0.0%	1.9%-3.1%
crt-Val141Leu	0/101	1/124	19/563	4/50	0/137	0/131	1/482	0/107	0/203	0/25	0/223	1/346	0/15	26/2507
	0.0%	0.8%	3.4%	8.0%	0.0%	0.0%	0.2%	0.0%	0.0%	0.0%	0.0%	0.3%	0.0%	1.0%
	0.0%-0.0%	0.0%-2.1%	2.1%-4.6%	1.7%-14.3%	0.0%-0.0%	0.0%-0.0%	0.0%-0.5%	0.0%-0.0%	0.0%-0.0%	0.0%-0.0%	0.0%-0.0%	0.0%-0.8%	0.0%-0.0%	0.7%-1.4%

crt-Val418Ala	no coverage	0/16	3/162	1/29	0/39	0/7	0/352	no coverage	0/20	0/5	0/6	0/206	0/1	4/843
	no coverage	0.0%	1.9%	3.4%	0.0%	0.0%	0.0%	no coverage	0.0%	0.0%	0.0%	0.0%	0.0%	0.5%
	no coverage	0.0%-0.0%	0.1%-3.6%	0.0%-9.0%	0.0%-0.0%	0.0%-0.0%	0.0%-0.0%	no coverage	0.0%-0.0%	0.0%-0.0%	0.0%-0.0%	0.0%-0.0%	0.0%-0.0%	0.1%-0.9%
cytb-Arg365Gln	0/234	0/267	0/636	0/41	3/239	0/285	0/299	0/266	0/346	0/79	0/409	0/316	0/73	3/3490
	0.0%	0.0%	0.0%	0.0%	1.3%	0.0%	0.0%	0.0%	0.0%	0.0%	0.0%	0.0%	0.0%	0.1%
	0.0%-0.0%	0.0%-0.0%	0.0%-0.0%	0.0%-0.0%	0.1%-2.4%	0.0%-0.0%	0.0%-0.0%	0.0%-0.0%	0.0%-0.0%	0.0%-0.0%	0.0%-0.0%	0.0%-0.0%	0.0%-0.0%	0.0%-0.2%
cytb-Cys354Trp	0/234	0/267	2/636	0/41	1/239	0/285	0/299	0/266	0/346	0/79	0/409	0/316	0/73	3/3490
	0.0%	0.0%	0.3%	0.0%	0.4%	0.0%	0.0%	0.0%	0.0%	0.0%	0.0%	0.0%	0.0%	0.1%
	0.0%-0.0%	0.0%-0.0%	0.0%-0.7%	0.0%-0.0%	0.0%-1.1%	0.0%-0.0%	0.0%-0.0%	0.0%-0.0%	0.0%-0.0%	0.0%-0.0%	0.0%-0.0%	0.0%-0.0%	0.0%-0.0%	0.0%-0.2%
cytb-His362Gln	0/234	0/267	1/636	0/41	0/239	3/285	0/299	0/266	0/346	0/79	3/409	2/316	1/73	10/3490
	0.0%	0.0%	0.2%	0.0%	0.0%	1.1%	0.0%	0.0%	0.0%	0.0%	0.7%	0.6%	1.4%	0.3%
	0.0%-0.0%	0.0%-0.0%	0.0%-0.4%	0.0%-0.0%	0.0%-0.0%	0.1%-2.0%	0.0%-0.0%	0.0%-0.0%	0.0%-0.0%	0.0%-0.0%	0.0%-1.4%	0.0%-1.4%	0.0%-3.6%	0.1%-0.4%
cytb-Leu206Phe	0/102	0/148	2/585	1/26	0/196	0/152	0/267	0/103	0/314	0/73	0/351	0/181	0/45	3/2543
	0.0%	0.0%	0.3%	3.8%	0.0%	0.0%	0.0%	0.0%	0.0%	0.0%	0.0%	0.0%	0.0%	0.1%
	0.0%-0.0%	0.0%-0.0%	0.0%-0.7%	0.0%-10.0%	0.0%-0.0%	0.0%-0.0%	0.0%-0.0%	0.0%-0.0%	0.0%-0.0%	0.0%-0.0%	0.0%-0.0%	0.0%-0.0%	0.0%-0.0%	0.0%-0.2%
cytb-Phe292Tyr	0/79	0/147	0/512	0/15	0/179	0/115	0/181	0/103	10/300	0/71	0/338	0/80	0/30	10/2150
	0.0%	0.0%	0.0%	0.0%	0.0%	0.0%	0.0%	0.0%	3.3%	0.0%	0.0%	0.0%	0.0%	0.5%
	0.0%-0.0%	0.0%-0.0%	0.0%-0.0%	0.0%-0.0%	0.0%-0.0%	0.0%-0.0%	0.0%-0.0%	0.0%-0.0%	1.6%-5.0%	0.0%-0.0%	0.0%-0.0%	0.0%-0.0%	0.0%-0.0%	0.2%-0.7%
cytb-Ser355Cys	0/234	1/267	1/636	0/41	2/239	0/285	0/299	0/266	1/346	0/79	0/409	1/316	0/73	6/3490
	0.0%	0.4%	0.2%	0.0%	0.8%	0.0%	0.0%	0.0%	0.3%	0.0%	0.0%	0.3%	0.0%	0.2%
	0.0%-0.0%	0.0%-1.0%	0.0%-0.4%	0.0%-0.0%	0.0%-1.8%	0.0%-0.0%	0.0%-0.0%	0.0%-0.0%	0.0%-0.8%	0.0%-0.0%	0.0%-0.0%	0.0%-0.8%	0.0%-0.0%	0.1%-0.3%
cytb-Val9Ile	0/56	0/176	0/488	0/21	0/198	0/199	0/205	0/92	0/275	0/76	3/297	0/51	0/38	3/2172
	0.0%	0.0%	0.0%	0.0%	0.0%	0.0%	0.0%	0.0%	0.0%	0.0%	1.0%	0.0%	0.0%	0.1%
	0.0%-0.0%	0.0%-0.0%	0.0%-0.0%	0.0%-0.0%	0.0%-0.0%	0.0%-0.0%	0.0%-0.0%	0.0%-0.0%	0.0%-0.0%	0.0%-0.0%	0.1%-2.0%	0.0%-0.0%	0.0%-0.0%	0.0%-0.3%
dhfr-ts-Asn51Ile	82/89	109/113	550/558	57/57	155/155	114/115	508/511	80/100	236/241	39/39	248/249	341/346	28/28	2547/2601
	92.1%	96.5%	98.6%	100.0%	100.0%	99.1%	99.4%	80.0%	97.9%	100.0%	99.6%	98.6%	100.0%	97.9%
	87.4%-96.8%	93.6%-99.3%	97.7%-99.4%	100.0%-100.0%	100.0%-100.0%	97.7%-100.0%	98.9%-100.0%	73.4%-86.6%	96.4%-99.4%	100.0%-100.0%	98.9%-100.0%	97.5%-99.6%	100.0%-100.0%	97.5%-98.4%
dhfr-ts-Cys59Arg	79/89	107/113	515/558	52/57	148/155	113/115	475/511	95/100	229/241	39/39	240/249	325/346	27/28	2444/2601
	88.8%	94.7%	92.3%	91.2%	95.5%	98.3%	93.0%	95.0%	95.0%	100.0%	96.4%	93.9%	96.4%	94.0%
	83.3%-94.3%	91.2%-98.2%	90.4%-94.2%	85.1%-97.4%	92.7%-98.2%	96.3%-100.0%	91.1%-94.8%	91.4%-98.6%	92.7%-97.3%	100.0%-100.0%	94.4%-98.3%	91.8%-96.0%	90.7%-100.0%	93.2%-94.7%
dhfr-ts-Ile164Leu	0/2	0/73	63/401	0/55	0/133	0/61	14/493	0/19	0/161	0/25	0/120	5/304	0/16	82/1863
	0.0%	0.0%	15.7%	0.0%	0.0%	0.0%	2.8%	0.0%	0.0%	0.0%	0.0%	1.6%	0.0%	4.4%
	0.0%-0.0%	0.0%-0.0%	12.7%-18.7%	0.0%-0.0%	0.0%-0.0%	0.0%-0.0%	1.6%-4.1%	0.0%-0.0%	0.0%-0.0%	0.0%-0.0%	0.0%-0.0%	0.4%-2.8%	0.0%-0.0%	3.6%-5.2%
dhfr-ts-Ser108Asn	29/29	83/83	486/486	55/55	140/140	72/72	490/490	79/79	198/198	33/33	182/182	307/309	17/17	2171/2173
	100.0%	100.0%	100.0%	100.0%	100.0%	100.0%	100.0%	100.0%	100.0%	100.0%	100.0%	99.4%	100.0%	99.9%
	100.0%-100.0%	100.0%-100.0%	100.0%-100.0%	100.0%-100.0%	100.0%-100.0%	100.0%-100.0%	100.0%-100.0%	100.0%-100.0%	100.0%-100.0%	100.0%-100.0%	100.0%-100.0%	98.6%-100.0%	100.0%-100.0%	99.8%-100.0%
dhps-Ala437Gly	34/43	82/86	483/509	64/64	133/140	81/87	482/499	47/65	195/214	26/29	173/186	309/318	14/16	2123/2256
	79.1%	95.3%	94.9%	100.0%	95.0%	93.1%	96.6%	72.3%	91.1%	89.7%	93.0%	97.2%	87.5%	94.1%
	68.9%-89.3%	91.6%-99.1%	93.3%-96.5%	100.0%-100.0%	92.0%-98.0%	88.6%-97.6%	95.3%-97.9%	63.2%-81.4%	87.9%-94.3%	80.4%-99.0%	89.9%-96.1%	95.6%-98.7%	73.9%-100.0%	93.3%-94.9%
dhps-Ala581Gly	14/117	6/138	157/631	26/65	57/172	37/161	3/519	0/139	12/270	2/46	4/291	16/409	14/40	348/2998
	12.0%	4.3%	24.9%	40.0%	33.1%	23.0%	0.6%	0.0%	4.4%	4.3%	1.4%	3.9%	35.0%	11.6%
	7.0%-16.9%	1.5%-7.2%	22.1%-27.7%	30.0%-50.0%	27.2%-39.0%	17.5%-28.4%	0.0%-1.1%	0.0%-0.0%	2.4%-6.5%	0.0%-9.3%	0.3%-2.5%	2.3%-5.5%	22.6%-47.4%	10.6%-12.6%
dhps-Ala613Ser	4/81	0/115	1/604	0/67	0/149	0/127	0/510	1/113	3/238	0/37	0/238	0/366	0/30	9/2675
	4.9%	0.0%	0.2%	0.0%	0.0%	0.0%	0.0%	0.9%	1.3%	0.0%	0.0%	0.0%	0.0%	0.3%
	1.0%-8.9%	0.0%-0.0%	0.0%-0.4%	0.0%-0.0%	0.0%-0.0%	0.0%-0.0%	0.0%-0.0%	0.0%-2.3%	0.1%-2.4%	0.0%-0.0%	0.0%-0.0%	0.0%-0.0%	0.0%-0.0%	0.2%-0.5%
dhps-Lys540Glu	83/97	118/124	547/582	56/56	152/159	124/129	503/517	85/107	225/250	37/40	239/259	348/358	27/32	2544/2710
	85.6%	95.2%	94.0%	100.0%	95.6%	96.1%	97.3%	79.4%	90.0%	92.5%	92.3%	97.2%	84.4%	93.9%
	79.7%-91.4%	92.0%-98.3%	92.4%-95.6%	100.0%-100.0%	92.9%-98.3%	93.3%-98.9%	96.1%-98.5%	73.0%-85.9%	86.9%-93.1%	85.6%-99.4%	89.5%-95.0%	95.8%-98.6%	73.8%-94.9%	93.1%-94.6%
dhps-Ser436Ala	4/43	3/86	2/509	0/64	3/140	5/87	5/499	4/65	4/214	0/29	3/186	3/318	1/16	37/2256

	9.3%	3.5%	0.4%	0.0%	2.1%	5.7%	1.0%	6.2%	1.9%	0.0%	1.6%	0.9%	6.2%	1.6%
	2.0%-16.6%	0.2%-6.7%	0.0%-0.8%	0.0%-0.0%	0.1%-4.2%	1.6%-9.9%	0.3%-1.7%	1.3%-11.1%	0.3%-3.4%	0.0%-0.0%	0.1%-3.1%	0.1%-1.8%	0.0%-16.2%	1.2%-2.1%
dhps-Ser436Phe	0/43	0/86	1/509	0/64	0/140	0/87	0/499	1/65	1/214	0/29	0/186	0/318	0/16	3/2256
	0.0%	0.0%	0.2%	0.0%	0.0%	0.0%	0.0%	1.5%	0.5%	0.0%	0.0%	0.0%	0.0%	0.1%
	0.0%-0.0%	0.0%-0.0%	0.0%-0.5%	0.0%-0.0%	0.0%-0.0%	0.0%-0.0%	0.0%-0.0%	0.0%-4.0%	0.0%-1.2%	0.0%-0.0%	0.0%-0.0%	0.0%-0.0%	0.0%-0.0%	0.0%-0.3%
dhps-Val622Leu	0/81	0/115	0/604	0/67	0/149	0/127	1/510	0/113	3/238	0/37	0/238	0/366	0/30	4/2675
	0.0%	0.0%	0.0%	0.0%	0.0%	0.0%	0.2%	0.0%	1.3%	0.0%	0.0%	0.0%	0.0%	0.1%
	0.0%-0.0%	0.0%-0.0%	0.0%-0.0%	0.0%-0.0%	0.0%-0.0%	0.0%-0.0%	0.0%-0.5%	0.0%-0.0%	0.1%-2.4%	0.0%-0.0%	0.0%-0.0%	0.0%-0.0%	0.0%-0.0%	0.0%-0.3%
exo-Asn369Asp	0/111	0/146	1/532	0/15	0/160	0/158	1/179	1/114	0/290	0/37	0/314	0/115	0/32	3/2203
	0.0%	0.0%	0.2%	0.0%	0.0%	0.0%	0.6%	0.9%	0.0%	0.0%	0.0%	0.0%	0.0%	0.1%
	0.0%-0.0%	0.0%-0.0%	0.0%-0.5%	0.0%-0.0%	0.0%-0.0%	0.0%-0.0%	0.0%-1.5%	0.0%-2.3%	0.0%-0.0%	0.0%-0.0%	0.0%-0.0%	0.0%-0.0%	0.0%-0.0%	0.0%-0.3%
exo-Asp393Asn	0/112	3/146	0/534	0/15	1/162	0/159	0/179	0/114	0/292	0/37	2/316	2/115	0/32	8/2213
	0.0%	2.1%	0.0%	0.0%	0.6%	0.0%	0.0%	0.0%	0.0%	0.0%	0.6%	1.7%	0.0%	0.4%
	0.0%-0.0%	0.1%-4.0%	0.0%-0.0%	0.0%-0.0%	0.0%-1.6%	0.0%-0.0%	0.0%-0.0%	0.0%-0.0%	0.0%-0.0%	0.0%-0.0%	0.0%-1.4%	0.0%-3.7%	0.0%-0.0%	0.2%-0.6%
exo-Val394Ile	0/112	0/146	3/534	0/15	0/162	0/159	0/179	0/114	0/292	0/37	0/316	0/115	0/32	3/2213
	0.0%	0.0%	0.6%	0.0%	0.0%	0.0%	0.0%	0.0%	0.0%	0.0%	0.0%	0.0%	0.0%	0.1%
	0.0%-0.0%	0.0%-0.0%	0.0%-1.1%	0.0%-0.0%	0.0%-0.0%	0.0%-0.0%	0.0%-0.0%	0.0%-0.0%	0.0%-0.0%	0.0%-0.0%	0.0%-0.0%	0.0%-0.0%	0.0%-0.0%	0.0%-0.3%
exo-Val410Ile	0/112	0/146	0/534	0/15	3/162	0/159	1/179	0/114	0/292	0/37	0/316	0/115	0/32	4/2213
	0.0%	0.0%	0.0%	0.0%	1.9%	0.0%	0.6%	0.0%	0.0%	0.0%	0.0%	0.0%	0.0%	0.2%
	0.0%-0.0%	0.0%-0.0%	0.0%-0.0%	0.0%-0.0%	0.1%-3.6%	0.0%-0.0%	0.0%-1.5%	0.0%-0.0%	0.0%-0.0%	0.0%-0.0%	0.0%-0.0%	0.0%-0.0%	0.0%-0.0%	0.0%-0.3%
fd-Asp193Tyr	0/100	3/145	95/527	5/13	1/168	1/156	0/168	0/102	3/264	0/40	13/310	1/113	1/24	123/2130
	0.0%	2.1%	18.0%	38.5%	0.6%	0.6%	0.0%	0.0%	1.1%	0.0%	4.2%	0.9%	4.2%	5.8%
	0.0%-0.0%	0.1%-4.0%	15.3%-20.8%	16.3%-60.7%	0.0%-1.6%	0.0%-1.7%	0.0%-0.0%	0.0%-0.0%	0.1%-2.2%	0.0%-0.0%	2.3%-6.1%	0.0%-2.3%	0.0%-10.9%	4.9%-6.6%
fd-Lys166Glu	0/100	0/145	0/527	0/13	4/168	0/156	0/168	0/102	0/264	1/40	0/310	0/113	0/24	5/2130
	0.0%	0.0%	0.0%	0.0%	2.4%	0.0%	0.0%	0.0%	0.0%	2.5%	0.0%	0.0%	0.0%	0.2%
	0.0%-0.0%	0.0%-0.0%	0.0%-0.0%	0.0%-0.0%	0.4%-4.3%	0.0%-0.0%	0.0%-0.0%	0.0%-0.0%	0.0%-0.0%	0.0%-6.6%	0.0%-0.0%	0.0%-0.0%	0.0%-0.0%	0.1%-0.4%
k13-Ala175Thr	0/2	0/66	8/311	0/40	0/111	0/56	0/440	0/1	0/111	0/18	0/80	0/247	0/10	8/1493
	0.0%	0.0%	2.6%	0.0%	0.0%	0.0%	0.0%	0.0%	0.0%	0.0%	0.0%	0.0%	0.0%	0.5%
	0.0%-0.0%	0.0%-0.0%	1.1%-4.0%	0.0%-0.0%	0.0%-0.0%	0.0%-0.0%	0.0%-0.0%	0.0%-0.0%	0.0%-0.0%	0.0%-0.0%	0.0%-0.0%	0.0%-0.0%	0.0%-0.0%	0.2%-0.8%
k13-Ala578Ser	0/81	0/127	1/596	0/67	0/137	0/115	4/504	1/105	0/254	0/31	1/299	1/372	0/11	8/2699
	0.0%	0.0%	0.2%	0.0%	0.0%	0.0%	0.8%	1.0%	0.0%	0.0%	0.3%	0.3%	0.0%	0.3%
	0.0%-0.0%	0.0%-0.0%	0.0%-0.4%	0.0%-0.0%	0.0%-0.0%	0.0%-0.0%	0.1%-1.4%	0.0%-2.5%	0.0%-0.0%	0.0%-0.0%	0.0%-0.9%	0.0%-0.7%	0.0%-0.0%	0.1%-0.5%
k13-Arg255Lys	1/110	1/120	9/519	3/57	3/159	3/133	28/511	1/116	9/217	1/41	3/224	7/339	3/37	72/2583
	0.9%	0.8%	1.7%	5.3%	1.9%	2.3%	5.5%	0.9%	4.1%	2.4%	1.3%	2.1%	8.1%	2.8%
	0.0%-2.4%	0.0%-2.2%	0.8%-2.7%	0.4%-10.1%	0.1%-3.7%	0.1%-4.4%	3.8%-7.1%	0.0%-2.3%	1.9%-6.4%	0.0%-6.4%	0.1%-2.6%	0.8%-3.3%	0.7%-15.5%	2.3%-3.3%
k13-Arg561His	0/42	0/86	36/466	0/48	0/105	1/87	0/457	0/66	0/174	0/16	0/186	2/295	0/4	39/2032
	0.0%	0.0%	7.7%	0.0%	0.0%	1.1%	0.0%	0.0%	0.0%	0.0%	0.0%	0.7%	0.0%	1.9%
	0.0%-0.0%	0.0%-0.0%	5.7%-9.8%	0.0%-0.0%	0.0%-0.0%	0.0%-3.0%	0.0%-0.0%	0.0%-0.0%	0.0%-0.0%	0.0%-0.0%	0.0%-0.0%	0.0%-1.5%	0.0%-0.0%	1.4%-2.4%
k13-Asn133Lys	0/3	0/65	1/321	0/41	0/117	0/53	2/435	0/16	0/114	0/22	0/90	1/250	0/2	4/1529
	0.0%	0.0%	0.3%	0.0%	0.0%	0.0%	0.5%	0.0%	0.0%	0.0%	0.0%	0.4%	0.0%	0.3%
	0.0%-0.0%	0.0%-0.0%	0.0%-0.8%	0.0%-0.0%	0.0%-0.0%	0.0%-0.0%	0.0%-1.0%	0.0%-0.0%	0.0%-0.0%	0.0%-0.0%	0.0%-0.0%	0.0%-1.1%	0.0%-0.0%	0.0%-0.5%
k13-Asn137Asp	0/3	0/65	1/321	0/41	0/117	0/53	1/435	0/16	0/114	0/22	0/90	1/250	0/2	3/1529
	0.0%	0.0%	0.3%	0.0%	0.0%	0.0%	0.2%	0.0%	0.0%	0.0%	0.0%	0.4%	0.0%	0.2%
	0.0%-0.0%	0.0%-0.0%	0.0%-0.8%	0.0%-0.0%	0.0%-0.0%	0.0%-0.0%	0.0%-0.6%	0.0%-0.0%	0.0%-0.0%	0.0%-0.0%	0.0%-0.0%	0.0%-1.1%	0.0%-0.0%	0.0%-0.4%
k13-Asn137Lys	0/3	0/65	1/321	0/41	0/117	0/53	1/435	0/16	0/114	0/22	0/90	1/250	0/2	3/1529
	0.0%	0.0%	0.3%	0.0%	0.0%	0.0%	0.2%	0.0%	0.0%	0.0%	0.0%	0.4%	0.0%	0.2%
	0.0%-0.0%	0.0%-0.0%	0.0%-0.8%	0.0%-0.0%	0.0%-0.0%	0.0%-0.0%	0.0%-0.6%	0.0%-0.0%	0.0%-0.0%	0.0%-0.0%	0.0%-0.0%	0.0%-1.1%	0.0%-0.0%	0.0%-0.4%
k13-Asn217His	0/20	0/72	3/394	0/50	0/132	0/66	3/469	0/31	0/176	1/29	0/132	3/272	0/21	10/1864
	0.0%	0.0%	0.8%	0.0%	0.0%	0.0%	0.6%	0.0%	0.0%	3.4%	0.0%	1.1%	0.0%	0.5%
	0.0%-0.0%	0.0%-0.0%	0.0%-1.5%	0.0%-0.0%	0.0%-0.0%	0.0%-0.0%	0.0%-1.2%	0.0%-0.0%	0.0%-0.0%	0.0%-9.0%	0.0%-0.0%	0.1%-2.1%	0.0%-0.0%	0.3%-0.8%

k13-Asn264Ser	0/110	0/120	1/519	0/57	0/159	0/133	1/511	0/116	0/217	0/41	0/224	2/339	0/37	4/2583
	0.0%	0.0%	0.2%	0.0%	0.0%	0.0%	0.2%	0.0%	0.0%	0.0%	0.0%	0.6%	0.0%	0.2%
	0.0%-0.0%	0.0%-0.0%	0.0%-0.5%	0.0%-0.0%	0.0%-0.0%	0.0%-0.0%	0.0%-0.5%	0.0%-0.0%	0.0%-0.0%	0.0%-0.0%	0.0%-0.0%	0.0%-1.3%	0.0%-0.0%	0.0%-0.3%
k13-Gln271Leu	0/100	0/104	1/492	0/47	0/147	0/115	2/491	0/102	0/178	0/33	0/198	0/314	0/28	3/2349
	0.0%	0.0%	0.2%	0.0%	0.0%	0.0%	0.4%	0.0%	0.0%	0.0%	0.0%	0.0%	0.0%	0.1%
	0.0%-0.0%	0.0%-0.0%	0.0%-0.5%	0.0%-0.0%	0.0%-0.0%	0.0%-0.0%	0.0%-0.9%	0.0%-0.0%	0.0%-0.0%	0.0%-0.0%	0.0%-0.0%	0.0%-0.0%	0.0%-0.0%	0.0%-0.2%
k13-Glu208Lys	0/20	0/72	8/394	0/50	0/132	0/66	0/469	0/31	0/176	0/29	0/132	0/272	0/21	8/1864
	0.0%	0.0%	2.0%	0.0%	0.0%	0.0%	0.0%	0.0%	0.0%	0.0%	0.0%	0.0%	0.0%	0.4%
	0.0%-0.0%	0.0%-0.0%	0.9%-3.2%	0.0%-0.0%	0.0%-0.0%	0.0%-0.0%	0.0%-0.0%	0.0%-0.0%	0.0%-0.0%	0.0%-0.0%	0.0%-0.0%	0.0%-0.0%	0.0%-0.0%	0.2%-0.7%
k13-Gly112Glu	0/3	0/65	1/321	0/41	0/117	0/53	2/435	0/16	0/114	0/22	0/90	0/250	0/2	3/1529
	0.0%	0.0%	0.3%	0.0%	0.0%	0.0%	0.5%	0.0%	0.0%	0.0%	0.0%	0.0%	0.0%	0.2%
	0.0%-0.0%	0.0%-0.0%	0.0%-0.8%	0.0%-0.0%	0.0%-0.0%	0.0%-0.0%	0.0%-1.0%	0.0%-0.0%	0.0%-0.0%	0.0%-0.0%	0.0%-0.0%	0.0%-0.0%	0.0%-0.0%	0.0%-0.4%
k13-His136Asn	0/3	0/65	0/321	0/41	0/117	1/53	1/435	0/16	1/114	0/22	0/90	3/250	1/2	7/1529
	0.0%	0.0%	0.0%	0.0%	0.0%	1.9%	0.2%	0.0%	0.9%	0.0%	0.0%	1.2%	50.0%	0.5%
	0.0%-0.0%	0.0%-0.0%	0.0%-0.0%	0.0%-0.0%	0.0%-0.0%	0.0%-5.0%	0.0%-0.6%	0.0%-0.0%	0.0%-2.3%	0.0%-0.0%	0.0%-0.0%	0.1%-2.3%	0.0%-100.0%	0.2%-0.7%
k13-His384Arg	0/17	0/72	4/411	0/46	0/129	0/58	0/468	0/62	0/187	0/25	1/157	0/282	0/4	5/1918
	0.0%	0.0%	1.0%	0.0%	0.0%	0.0%	0.0%	0.0%	0.0%	0.0%	0.6%	0.0%	0.0%	0.3%
	0.0%-0.0%	0.0%-0.0%	0.2%-1.8%	0.0%-0.0%	0.0%-0.0%	0.0%-0.0%	0.0%-0.0%	0.0%-0.0%	0.0%-0.0%	0.0%-0.0%	0.0%-1.7%	0.0%-0.0%	0.0%-0.0%	0.1%-0.5%
k13-Ile205Thr	0/2	0/66	6/306	0/40	0/110	0/53	0/427	0/1	0/107	0/16	0/77	1/246	0/9	7/1460
	0.0%	0.0%	2.0%	0.0%	0.0%	0.0%	0.0%	0.0%	0.0%	0.0%	0.0%	0.4%	0.0%	0.5%
	0.0%-0.0%	0.0%-0.0%	0.7%-3.3%	0.0%-0.0%	0.0%-0.0%	0.0%-0.0%	0.0%-0.0%	0.0%-0.0%	0.0%-0.0%	0.0%-0.0%	0.0%-0.0%	0.0%-1.1%	0.0%-0.0%	0.2%-0.8%
k13-Leu116Ile	0/3	0/65	1/321	0/41	0/117	0/53	3/435	0/16	0/114	0/22	0/90	2/250	0/2	6/1529
	0.0%	0.0%	0.3%	0.0%	0.0%	0.0%	0.7%	0.0%	0.0%	0.0%	0.0%	0.8%	0.0%	0.4%
	0.0%-0.0%	0.0%-0.0%	0.0%-0.8%	0.0%-0.0%	0.0%-0.0%	0.0%-0.0%	0.0%-1.3%	0.0%-0.0%	0.0%-0.0%	0.0%-0.0%	0.0%-0.0%	0.0%-1.7%	0.0%-0.0%	0.1%-0.7%
k13-Leu177Ile	0/2	0/66	0/306	0/40	0/110	0/53	3/427	0/1	0/107	0/16	0/77	0/246	0/9	3/1460
	0.0%	0.0%	0.0%	0.0%	0.0%	0.0%	0.7%	0.0%	0.0%	0.0%	0.0%	0.0%	0.0%	0.2%
	0.0%-0.0%	0.0%-0.0%	0.0%-0.0%	0.0%-0.0%	0.0%-0.0%	0.0%-0.0%	0.0%-1.4%	0.0%-0.0%	0.0%-0.0%	0.0%-0.0%	0.0%-0.0%	0.0%-0.0%	0.0%-0.0%	0.0%-0.4%
k13-Leu258Met	4/110	3/120	6/519	1/57	3/159	1/133	10/511	1/116	12/217	2/41	3/224	17/339	0/37	63/2583
	3.6%	2.5%	1.2%	1.8%	1.9%	0.8%	2.0%	0.9%	5.5%	4.9%	1.3%	5.0%	0.0%	2.4%
	0.7%-6.6%	0.2%-4.8%	0.4%-1.9%	0.0%-4.6%	0.1%-3.7%	0.0%-2.0%	0.9%-3.0%	0.0%-2.3%	3.0%-8.1%	0.0%-10.4%	0.1%-2.6%	3.1%-7.0%	0.0%-0.0%	1.9%-2.9%
k13-Leu99Val	0/2	0/68	0/359	0/54	0/120	0/53	1/470	0/20	0/153	0/22	0/99	4/295	0/2	5/1717
	0.0%	0.0%	0.0%	0.0%	0.0%	0.0%	0.2%	0.0%	0.0%	0.0%	0.0%	1.4%	0.0%	0.3%
	0.0%-0.0%	0.0%-0.0%	0.0%-0.0%	0.0%-0.0%	0.0%-0.0%	0.0%-0.0%	0.0%-0.6%	0.0%-0.0%	0.0%-0.0%	0.0%-0.0%	0.0%-0.0%	0.2%-2.5%	0.0%-0.0%	0.1%-0.5%
k13-Lys108Glu	0/3	0/65	0/321	0/41	4/117	0/53	1/435	0/16	0/114	0/22	0/90	0/250	0/2	5/1529
	0.0%	0.0%	0.0%	0.0%	3.4%	0.0%	0.2%	0.0%	0.0%	0.0%	0.0%	0.0%	0.0%	0.3%
	0.0%-0.0%	0.0%-0.0%	0.0%-0.0%	0.0%-0.0%	0.7%-6.2%	0.0%-0.0%	0.0%-0.6%	0.0%-0.0%	0.0%-0.0%	0.0%-0.0%	0.0%-0.0%	0.0%-0.0%	0.0%-0.0%	0.1%-0.6%
k13-Lys124Asn	0/3	0/65	0/321	0/41	0/117	0/53	5/435	0/16	0/114	0/22	0/90	1/250	0/2	6/1529
	0.0%	0.0%	0.0%	0.0%	0.0%	0.0%	1.1%	0.0%	0.0%	0.0%	0.0%	0.4%	0.0%	0.4%
	0.0%-0.0%	0.0%-0.0%	0.0%-0.0%	0.0%-0.0%	0.0%-0.0%	0.0%-0.0%	0.3%-2.0%	0.0%-0.0%	0.0%-0.0%	0.0%-0.0%	0.0%-0.0%	0.0%-1.1%	0.0%-0.0%	0.1%-0.7%
k13-Lys189Asn	0/2	0/66	1/306	0/40	0/110	0/53	2/427	0/1	0/107	3/16	0/77	5/246	0/9	11/1460
	0.0%	0.0%	0.3%	0.0%	0.0%	0.0%	0.5%	0.0%	0.0%	18.8%	0.0%	2.0%	0.0%	0.8%
	0.0%-0.0%	0.0%-0.0%	0.0%-0.9%	0.0%-0.0%	0.0%-0.0%	0.0%-0.0%	0.0%-1.0%	0.0%-0.0%	0.0%-0.0%	2.7%-34.8%	0.0%-0.0%	0.6%-3.5%	0.0%-0.0%	0.4%-1.1%
k13-Lys189Thr	0/2	5/66	61/306	13/40	18/110	7/53	104/427	0/1	12/107	1/16	10/77	68/246	1/9	300/1460
	0.0%	7.6%	19.9%	32.5%	16.4%	13.2%	24.4%	0.0%	11.2%	6.2%	13.0%	27.6%	11.1%	20.5%
	0.0%-0.0%	2.2%-12.9%	16.2%-23.7%	20.3%-44.7%	10.6%-22.2%	5.6%-20.9%	20.9%-27.8%	0.0%-0.0%	6.2%-16.2%	0.0%-16.2%	6.7%-19.3%	23.0%-32.3%	0.0%-28.3%	18.8%-22.3%
k13-Lys9Arg	0/6	0/65	0/358	0/51	0/118	0/55	0/468	0/14	3/144	0/19	0/94	0/279	0/5	3/1676
	0.0%	0.0%	0.0%	0.0%	0.0%	0.0%	0.0%	0.0%	2.1%	0.0%	0.0%	0.0%	0.0%	0.2%
	0.0%-0.0%	0.0%-0.0%	0.0%-0.0%	0.0%-0.0%	0.0%-0.0%	0.0%-0.0%	0.0%-0.0%	0.0%-0.0%	0.1%-4.0%	0.0%-0.0%	0.0%-0.0%	0.0%-0.0%	0.0%-0.0%	0.0%-0.3%
k13-Phe699Cys	0/32	0/73	3/410	0/50	0/138	0/67	0/485	0/36	0/195	0/33	0/139	0/291	0/21	3/1970
	0.0%	0.0%	0.7%	0.0%	0.0%	0.0%	0.0%	0.0%	0.0%	0.0%	0.0%	0.0%	0.0%	0.2%

	0.0%-0.0%	0.0%-0.0%	0.0%-1.4%	0.0%-0.0%	0.0%-0.0%	0.0%-0.0%	0.0%-0.0%	0.0%-0.0%	0.0%-0.0%	0.0%-0.0%	0.0%-0.0%	0.0%-0.0%	0.0%-0.0%	0.0%-0.3%
k13-Pro419Ser	0/97	0/126	5/591	0/66	0/170	0/126	0/520	0/116	0/281	0/44	0/306	1/374	0/36	6/2853
	0.0%	0.0%	0.8%	0.0%	0.0%	0.0%	0.0%	0.0%	0.0%	0.0%	0.0%	0.3%	0.0%	0.2%
	0.0%-0.0%	0.0%-0.0%	0.2%-1.5%	0.0%-0.0%	0.0%-0.0%	0.0%-0.0%	0.0%-0.0%	0.0%-0.0%	0.0%-0.0%	0.0%-0.0%	0.0%-0.0%	0.0%-0.7%	0.0%-0.0%	0.1%-0.4%
k13-Pro667Ser	0/51	0/101	2/531	0/69	0/161	0/104	3/509	0/76	0/250	0/42	0/236	0/336	0/31	5/2497
	0.0%	0.0%	0.4%	0.0%	0.0%	0.0%	0.6%	0.0%	0.0%	0.0%	0.0%	0.0%	0.0%	0.2%
	0.0%-0.0%	0.0%-0.0%	0.0%-0.8%	0.0%-0.0%	0.0%-0.0%	0.0%-0.0%	0.0%-1.1%	0.0%-0.0%	0.0%-0.0%	0.0%-0.0%	0.0%-0.0%	0.0%-0.0%	0.0%-0.0%	0.1%-0.3%
k13-Pro667Thr	0/51	0/101	0/531	0/69	0/161	0/104	0/509	0/76	3/250	0/42	0/236	0/336	0/31	3/2497
	0.0%	0.0%	0.0%	0.0%	0.0%	0.0%	0.0%	0.0%	1.2%	0.0%	0.0%	0.0%	0.0%	0.1%
	0.0%-0.0%	0.0%-0.0%	0.0%-0.0%	0.0%-0.0%	0.0%-0.0%	0.0%-0.0%	0.0%-0.0%	0.0%-0.0%	0.1%-2.3%	0.0%-0.0%	0.0%-0.0%	0.0%-0.0%	0.0%-0.0%	0.0%-0.2%
k13-Ser182Thr	0/2	0/66	4/306	0/40	0/110	0/53	2/427	0/1	1/107	0/16	1/77	2/246	0/9	10/1460
	0.0%	0.0%	1.3%	0.0%	0.0%	0.0%	0.5%	0.0%	0.9%	0.0%	1.3%	0.8%	0.0%	0.7%
	0.0%-0.0%	0.0%-0.0%	0.2%-2.4%	0.0%-0.0%	0.0%-0.0%	0.0%-0.0%	0.0%-1.0%	0.0%-0.0%	0.0%-2.5%	0.0%-0.0%	0.0%-3.4%	0.0%-1.8%	0.0%-0.0%	0.3%-1.0%
k13-Thr149Ser	0/3	0/65	1/321	0/41	0/117	0/53	12/435	0/16	0/114	0/22	1/90	2/250	0/2	16/1529
	0.0%	0.0%	0.3%	0.0%	0.0%	0.0%	2.8%	0.0%	0.0%	0.0%	1.1%	0.8%	0.0%	1.0%
	0.0%-0.0%	0.0%-0.0%	0.0%-0.8%	0.0%-0.0%	0.0%-0.0%	0.0%-0.0%	1.5%-4.1%	0.0%-0.0%	0.0%-0.0%	0.0%-0.0%	0.0%-2.9%	0.0%-1.7%	0.0%-0.0%	0.6%-1.5%
k13-Thr207Lys	0/20	0/72	3/394	0/50	0/132	0/66	1/469	0/31	0/175	0/29	0/131	0/272	0/21	4/1862
	0.0%	0.0%	0.8%	0.0%	0.0%	0.0%	0.2%	0.0%	0.0%	0.0%	0.0%	0.0%	0.0%	0.2%
	0.0%-0.0%	0.0%-0.0%	0.0%-1.5%	0.0%-0.0%	0.0%-0.0%	0.0%-0.0%	0.0%-0.6%	0.0%-0.0%	0.0%-0.0%	0.0%-0.0%	0.0%-0.0%	0.0%-0.0%	0.0%-0.0%	0.0%-0.4%
k13-Thr348Ile	0/12	0/73	5/386	0/46	0/141	0/60	0/467	0/48	0/158	0/30	0/130	0/279	0/18	5/1848
	0.0%	0.0%	1.3%	0.0%	0.0%	0.0%	0.0%	0.0%	0.0%	0.0%	0.0%	0.0%	0.0%	0.3%
	0.0%-0.0%	0.0%-0.0%	0.3%-2.2%	0.0%-0.0%	0.0%-0.0%	0.0%-0.0%	0.0%-0.0%	0.0%-0.0%	0.0%-0.0%	0.0%-0.0%	0.0%-0.0%	0.0%-0.0%	0.0%-0.0%	0.1%-0.5%
k13-Val555Ala	0/42	0/86	3/466	0/48	0/105	0/87	0/457	0/66	0/174	0/16	0/186	0/295	0/4	3/2032
	0.0%	0.0%	0.6%	0.0%	0.0%	0.0%	0.0%	0.0%	0.0%	0.0%	0.0%	0.0%	0.0%	0.1%
	0.0%-0.0%	0.0%-0.0%	0.0%-1.3%	0.0%-0.0%	0.0%-0.0%	0.0%-0.0%	0.0%-0.0%	0.0%-0.0%	0.0%-0.0%	0.0%-0.0%	0.0%-0.0%	0.0%-0.0%	0.0%-0.0%	0.0%-0.3%
kelch10-Glu668Lys	1/48	0/95	2/429	0/12	0/158	0/92	0/153	0/76	3/251	0/33	2/262	0/40	0/26	8/1675
	2.1%	0.0%	0.5%	0.0%	0.0%	0.0%	0.0%	0.0%	1.2%	0.0%	0.8%	0.0%	0.0%	0.5%
	0.0%-5.5%	0.0%-0.0%	0.0%-1.0%	0.0%-0.0%	0.0%-0.0%	0.0%-0.0%	0.0%-0.0%	0.0%-0.0%	0.1%-2.3%	0.0%-0.0%	0.0%-1.6%	0.0%-0.0%	0.0%-0.0%	0.2%-0.8%
kelch10-Leu622Ile	0/14	0/70	3/312	0/4	1/130	1/58	0/128	0/47	4/176	0/24	0/134	0/17	0/9	9/1123
	0.0%	0.0%	1.0%	0.0%	0.8%	1.7%	0.0%	0.0%	2.3%	0.0%	0.0%	0.0%	0.0%	0.8%
	0.0%-0.0%	0.0%-0.0%	0.1%-1.9%	0.0%-0.0%	0.0%-2.0%	0.0%-4.5%	0.0%-0.0%	0.0%-0.0%	0.4%-4.1%	0.0%-0.0%	0.0%-0.0%	0.0%-0.0%	0.0%-0.0%	0.4%-1.2%
kelch10-Thr636Ser	0/48	0/95	4/429	0/12	0/158	0/92	2/153	0/76	4/251	0/33	1/262	0/39	0/26	11/1674
	0.0%	0.0%	0.9%	0.0%	0.0%	0.0%	1.3%	0.0%	1.6%	0.0%	0.4%	0.0%	0.0%	0.7%
	0.0%-0.0%	0.0%-0.0%	0.2%-1.7%	0.0%-0.0%	0.0%-0.0%	0.0%-0.0%	0.0%-2.8%	0.0%-0.0%	0.3%-2.9%	0.0%-0.0%	0.0%-1.0%	0.0%-0.0%	0.0%-0.0%	0.3%-1.0%
mcp-Pro280Thr	0/4	4/69	3/292	0/2	1/132	4/55	1/128	0/41	3/145	0/26	0/121	0/20	0/8	16/1043
	0.0%	5.8%	1.0%	0.0%	0.8%	7.3%	0.8%	0.0%	2.1%	0.0%	0.0%	0.0%	0.0%	1.5%
	0.0%-0.0%	1.2%-10.4%	0.1%-2.0%	0.0%-0.0%	0.0%-2.0%	1.5%-13.0%	0.0%-2.1%	0.0%-0.0%	0.1%-4.0%	0.0%-0.0%	0.0%-0.0%	0.0%-0.0%	0.0%-0.0%	0.9%-2.2%
mdr1-Ala891Thr	0/23	0/66	0/333	0/41	2/122	1/58	0/460	0/20	0/134	0/22	0/92	0/270	0/10	3/1651
	0.0%	0.0%	0.0%	0.0%	1.6%	1.7%	0.0%	0.0%	0.0%	0.0%	0.0%	0.0%	0.0%	0.2%
	0.0%-0.0%	0.0%-0.0%	0.0%-0.0%	0.0%-0.0%	0.0%-3.5%	0.0%-4.5%	0.0%-0.0%	0.0%-0.0%	0.0%-0.0%	0.0%-0.0%	0.0%-0.0%	0.0%-0.0%	0.0%-0.0%	0.0%-0.4%
mdr1-Asn1042Asp	0/146	0/169	2/629	0/71	0/198	0/187	0/536	0/185	3/276	0/62	0/276	0/430	0/56	5/3221
	0.0%	0.0%	0.3%	0.0%	0.0%	0.0%	0.0%	0.0%	1.1%	0.0%	0.0%	0.0%	0.0%	0.2%
	0.0%-0.0%	0.0%-0.0%	0.0%-0.7%	0.0%-0.0%	0.0%-0.0%	0.0%-0.0%	0.0%-0.0%	0.0%-0.0%	0.1%-2.1%	0.0%-0.0%	0.0%-0.0%	0.0%-0.0%	0.0%-0.0%	0.0%-0.3%
mdr1-Asn1199Ser	no coverage	0/65	0/339	0/53	0/105	0/50	2/462	no coverage	1/108	0/13	0/70	0/289	0/1	3/1555
	no coverage	0.0%	0.0%	0.0%	0.0%	0.0%	0.4%	no coverage	0.9%	0.0%	0.0%	0.0%	0.0%	0.2%
	no coverage	0.0%-0.0%	0.0%-0.0%	0.0%-0.0%	0.0%-0.0%	0.0%-0.0%	0.0%-0.9%	no coverage	0.0%-2.4%	0.0%-0.0%	0.0%-0.0%	0.0%-0.0%	0.0%-0.0%	0.0%-0.4%
mdr1-Asn12Thr	0/24	0/70	0/408	0/62	2/141	1/64	1/498	0/13	0/187	0/30	0/119	0/315	0/20	4/1951
	0.0%	0.0%	0.0%	0.0%	1.4%	1.6%	0.2%	0.0%	0.0%	0.0%	0.0%	0.0%	0.0%	0.2%
	0.0%-0.0%	0.0%-0.0%	0.0%-0.0%	0.0%-0.0%	0.0%-3.1%	0.0%-4.1%	0.0%-0.5%	0.0%-0.0%	0.0%-0.0%	0.0%-0.0%	0.0%-0.0%	0.0%-0.0%	0.0%-0.0%	0.0%-0.4%
mdr1-Asn504Lys	0/5	0/74	5/365	0/39	0/137	0/63	4/442	0/26	0/166	0/29	3/125	5/250	0/23	17/1744

	0.0%	0.0%	1.4%	0.0%	0.0%	0.0%	0.9%	0.0%	0.0%	0.0%	2.4%	2.0%	0.0%	1.0%
	0.0%-0.0%	0.0%-0.0%	0.4%-2.4%	0.0%-0.0%	0.0%-0.0%	0.0%-0.0%	0.2%-1.6%	0.0%-0.0%	0.0%-0.0%	0.0%-0.0%	0.1%-4.7%	0.5%-3.5%	0.0%-0.0%	0.6%-1.4%
mdr1-Asn649Asp	0/56	0/94	8/497	1/47	1/136	0/91	14/468	0/71	2/198	0/31	1/197	9/290	0/12	36/2188
	0.0%	0.0%	1.6%	2.1%	0.7%	0.0%	3.0%	0.0%	1.0%	0.0%	0.5%	3.1%	0.0%	1.6%
	0.0%-0.0%	0.0%-0.0%	0.7%-2.5%	0.0%-5.6%	0.0%-1.9%	0.0%-0.0%	1.7%-4.3%	0.0%-0.0%	0.0%-2.2%	0.0%-0.0%	0.0%-1.3%	1.4%-4.8%	0.0%-0.0%	1.2%-2.1%
mdr1-Asn652Asp	3/56	1/94	12/497	5/47	2/136	1/91	22/468	3/71	3/198	2/31	7/197	6/290	0/12	67/2188
	5.4%	1.1%	2.4%	10.6%	1.5%	1.1%	4.7%	4.2%	1.5%	6.5%	3.6%	2.1%	0.0%	3.1%
	0.4%-10.3%	0.0%-2.8%	1.3%-3.5%	3.2%-18.0%	0.0%-3.2%	0.0%-2.9%	3.1%-6.3%	0.3%-8.2%	0.1%-2.9%	0.0%-13.7%	1.4%-5.7%	0.7%-3.4%	0.0%-0.0%	2.5%-3.7%
mdr1-Asn653Asp	0/56	0/94	3/497	0/47	0/136	0/91	6/468	0/71	0/198	0/31	0/197	0/290	0/12	9/2188
	0.0%	0.0%	0.6%	0.0%	0.0%	0.0%	1.3%	0.0%	0.0%	0.0%	0.0%	0.0%	0.0%	0.4%
	0.0%-0.0%	0.0%-0.0%	0.0%-1.2%	0.0%-0.0%	0.0%-0.0%	0.0%-0.0%	0.4%-2.1%	0.0%-0.0%	0.0%-0.0%	0.0%-0.0%	0.0%-0.0%	0.0%-0.0%	0.0%-0.0%	0.2%-0.6%
mdr1-Asn86Phe	0/130	0/139	1/595	0/48	0/160	0/144	0/486	0/124	2/261	0/38	0/260	0/356	0/26	3/2767
	0.0%	0.0%	0.2%	0.0%	0.0%	0.0%	0.0%	0.0%	0.8%	0.0%	0.0%	0.0%	0.0%	0.1%
	0.0%-0.0%	0.0%-0.0%	0.0%-0.4%	0.0%-0.0%	0.0%-0.0%	0.0%-0.0%	0.0%-0.0%	0.0%-0.0%	0.0%-1.7%	0.0%-0.0%	0.0%-0.0%	0.0%-0.0%	0.0%-0.0%	0.0%-0.2%
mdr1-Asn86Tyr	0/130	0/139	3/595	0/48	1/160	0/144	1/486	0/124	2/261	0/38	0/260	1/356	0/26	8/2767
	0.0%	0.0%	0.5%	0.0%	0.6%	0.0%	0.2%	0.0%	0.8%	0.0%	0.0%	0.3%	0.0%	0.3%
	0.0%-0.0%	0.0%-0.0%	0.0%-1.0%	0.0%-0.0%	0.0%-1.6%	0.0%-0.0%	0.0%-0.5%	0.0%-0.0%	0.0%-1.7%	0.0%-0.0%	0.0%-0.0%	0.0%-0.7%	0.0%-0.0%	0.1%-0.5%
mdr1-Asp1246Tyr	0/5	0/68	12/415	4/63	3/129	2/56	14/487	0/36	0/169	0/19	1/126	16/314	1/8	53/1895
	0.0%	0.0%	2.9%	6.3%	2.3%	3.6%	2.9%	0.0%	0.0%	0.0%	0.8%	5.1%	12.5%	2.8%
	0.0%-0.0%	0.0%-0.0%	1.5%-4.2%	1.3%-11.4%	0.1%-4.5%	0.0%-7.7%	1.6%-4.1%	0.0%-0.0%	0.0%-0.0%	0.0%-0.0%	0.0%-2.1%	3.1%-7.1%	0.0%-31.7%	2.2%-3.4%
mdr1-Asp543Gly	0/96	0/115	0/567	0/45	1/143	0/118	2/477	0/103	0/222	0/28	0/229	0/305	0/15	3/2463
	0.0%	0.0%	0.0%	0.0%	0.7%	0.0%	0.4%	0.0%	0.0%	0.0%	0.0%	0.0%	0.0%	0.1%
	0.0%-0.0%	0.0%-0.0%	0.0%-0.0%	0.0%-0.0%	0.0%-1.8%	0.0%-0.0%	0.0%-0.9%	0.0%-0.0%	0.0%-0.0%	0.0%-0.0%	0.0%-0.0%	0.0%-0.0%	0.0%-0.0%	0.0%-0.2%
mdr1-Asp642Asn	0/56	2/94	0/497	0/47	0/136	0/91	4/468	0/71	1/198	0/31	2/197	12/290	0/12	21/2188
	0.0%	2.1%	0.0%	0.0%	0.0%	0.0%	0.9%	0.0%	0.5%	0.0%	1.0%	4.1%	0.0%	1.0%
	0.0%-0.0%	0.0%-4.6%	0.0%-0.0%	0.0%-0.0%	0.0%-0.0%	0.0%-0.0%	0.2%-1.6%	0.0%-0.0%	0.0%-1.3%	0.0%-0.0%	0.0%-2.2%	2.2%-6.1%	0.0%-0.0%	0.6%-1.3%
mdr1-Asp650Asn	0/56	1/94	8/497	3/47	3/136	3/91	24/468	2/71	5/198	2/31	5/197	8/290	0/12	64/2188
	0.0%	1.1%	1.6%	6.4%	2.2%	3.3%	5.1%	2.8%	2.5%	6.5%	2.5%	2.8%	0.0%	2.9%
	0.0%-0.0%	0.0%-2.8%	0.7%-2.5%	0.5%-12.2%	0.1%-4.3%	0.2%-6.4%	3.5%-6.8%	0.0%-6.0%	0.7%-4.4%	0.0%-13.7%	0.7%-4.4%	1.2%-4.3%	0.0%-0.0%	2.3%-3.5%
mdr1-Asp651Asn	1/56	0/94	16/497	2/47	3/136	0/91	35/468	1/71	2/198	0/31	4/197	20/290	0/12	84/2188
	1.8%	0.0%	3.2%	4.3%	2.2%	0.0%	7.5%	1.4%	1.0%	0.0%	2.0%	6.9%	0.0%	3.8%
	0.0%-4.7%	0.0%-0.0%	1.9%-4.5%	0.0%-9.1%	0.1%-4.3%	0.0%-0.0%	5.5%-9.5%	0.0%-3.7%	0.0%-2.2%	0.0%-0.0%	0.4%-3.7%	4.4%-9.3%	0.0%-0.0%	3.2%-4.5%
mdr1-Asp651Tyr	0/56	0/94	0/497	2/47	0/136	0/91	1/468	0/71	0/198	0/31	0/197	1/290	0/12	4/2188
	0.0%	0.0%	0.0%	4.3%	0.0%	0.0%	0.2%	0.0%	0.0%	0.0%	0.0%	0.3%	0.0%	0.2%
	0.0%-0.0%	0.0%-0.0%	0.0%-0.0%	0.0%-9.1%	0.0%-0.0%	0.0%-0.0%	0.0%-0.6%	0.0%-0.0%	0.0%-0.0%	0.0%-0.0%	0.0%-0.0%	0.0%-0.9%	0.0%-0.0%	0.0%-0.3%
mdr1-Gln1198Lys	no coverage	0/65	2/339	0/53	0/105	0/50	3/462	no coverage	0/108	0/13	0/70	0/289	0/1	5/1555
	no coverage	0.0%	0.6%	0.0%	0.0%	0.0%	0.6%	no coverage	0.0%	0.0%	0.0%	0.0%	0.0%	0.3%
	no coverage	0.0%-0.0%	0.0%-1.3%	0.0%-0.0%	0.0%-0.0%	0.0%-0.0%	0.0%-1.3%	no coverage	0.0%-0.0%	0.0%-0.0%	0.0%-0.0%	0.0%-0.0%	0.0%-0.0%	0.1%-0.6%
mdr1-Glu348Gly	0/21	0/80	0/475	0/50	0/136	0/75	0/480	0/54	3/214	0/30	0/206	0/292	0/10	3/2123
	0.0%	0.0%	0.0%	0.0%	0.0%	0.0%	0.0%	0.0%	1.4%	0.0%	0.0%	0.0%	0.0%	0.1%
	0.0%-0.0%	0.0%-0.0%	0.0%-0.0%	0.0%-0.0%	0.0%-0.0%	0.0%-0.0%	0.0%-0.0%	0.0%-0.0%	0.1%-2.7%	0.0%-0.0%	0.0%-0.0%	0.0%-0.0%	0.0%-0.0%	0.0%-0.3%
mdr1-Glu751Asp	0/90	5/127	5/568	0/49	0/158	0/128	7/489	0/103	0/231	0/36	0/222	0/319	0/32	17/2552
	0.0%	3.9%	0.9%	0.0%	0.0%	0.0%	1.4%	0.0%	0.0%	0.0%	0.0%	0.0%	0.0%	0.7%
	0.0%-0.0%	1.1%-6.8%	0.2%-1.5%	0.0%-0.0%	0.0%-0.0%	0.0%-0.0%	0.5%-2.3%	0.0%-0.0%	0.0%-0.0%	0.0%-0.0%	0.0%-0.0%	0.0%-0.0%	0.0%-0.0%	0.4%-0.9%
mdr1-Glu7Gln	0/24	0/70	1/408	0/62	0/141	0/64	7/498	0/13	1/187	0/30	1/119	1/315	1/20	12/1951
	0.0%	0.0%	0.2%	0.0%	0.0%	0.0%	1.4%	0.0%	0.5%	0.0%	0.8%	0.3%	5.0%	0.6%
	0.0%-0.0%	0.0%-0.0%	0.0%-0.6%	0.0%-0.0%	0.0%-0.0%	0.0%-0.0%	0.5%-2.3%	0.0%-0.0%	0.0%-1.4%	0.0%-0.0%	0.0%-2.2%	0.0%-0.8%	0.0%-13.0%	0.3%-0.9%
mdr1-Gly968Ala	0/115	0/138	0/577	0/72	0/176	0/148	0/526	1/149	1/267	0/54	0/263	2/381	0/46	4/2912
	0.0%	0.0%	0.0%	0.0%	0.0%	0.0%	0.0%	0.7%	0.4%	0.0%	0.0%	0.5%	0.0%	0.1%
	0.0%-0.0%	0.0%-0.0%	0.0%-0.0%	0.0%-0.0%	0.0%-0.0%	0.0%-0.0%	0.0%-0.0%	0.0%-1.8%	0.0%-1.0%	0.0%-0.0%	0.0%-0.0%	0.0%-1.1%	0.0%-0.0%	0.0%-0.3%

mdr1-Ile839Val	0/7	0/71	7/407	0/47	0/134	0/63	7/477	0/38	0/184	0/28	3/142	5/291	0/13	22/1902
	0.0%	0.0%	1.7%	0.0%	0.0%	0.0%	1.5%	0.0%	0.0%	0.0%	2.1%	1.7%	0.0%	1.2%
	0.0%-0.0%	0.0%-0.0%	0.7%-2.8%	0.0%-0.0%	0.0%-0.0%	0.0%-0.0%	0.6%-2.4%	0.0%-0.0%	0.0%-0.0%	0.0%-0.0%	0.1%-4.1%	0.5%-3.0%	0.0%-0.0%	0.8%-1.6%
mdr1-Leu429Phe	0/55	1/91	0/460	0/50	1/152	2/75	0/489	0/67	0/213	0/40	0/186	1/306	0/29	5/2213
	0.0%	1.1%	0.0%	0.0%	0.7%	2.7%	0.0%	0.0%	0.0%	0.0%	0.0%	0.3%	0.0%	0.2%
	0.0%-0.0%	0.0%-2.9%	0.0%-0.0%	0.0%-0.0%	0.0%-1.7%	0.0%-5.7%	0.0%-0.0%	0.0%-0.0%	0.0%-0.0%	0.0%-0.0%	0.0%-0.0%	0.0%-0.9%	0.0%-0.0%	0.1%-0.4%
mdr1-Leu836Phe	0/7	0/71	0/407	0/47	0/134	0/63	0/477	0/38	0/184	0/28	0/142	3/291	0/13	3/1902
	0.0%	0.0%	0.0%	0.0%	0.0%	0.0%	0.0%	0.0%	0.0%	0.0%	0.0%	1.0%	0.0%	0.2%
	0.0%-0.0%	0.0%-0.0%	0.0%-0.0%	0.0%-0.0%	0.0%-0.0%	0.0%-0.0%	0.0%-0.0%	0.0%-0.0%	0.0%-0.0%	0.0%-0.0%	0.0%-0.0%	0.1%-2.0%	0.0%-0.0%	0.0%-0.3%
mdr1-Lys34Arg	no coverage	0/19	1/168	0/38	0/23	0/11	3/382	no coverage	0/62	0/9	0/28	1/241	no coverage	5/981
	no coverage	0.0%	0.6%	0.0%	0.0%	0.0%	0.8%	no coverage	0.0%	0.0%	0.0%	0.4%	no coverage	0.5%
	no coverage	0.0%-0.0%	0.0%-1.6%	0.0%-0.0%	0.0%-0.0%	0.0%-0.0%	0.0%-1.5%	no coverage	0.0%-0.0%	0.0%-0.0%	0.0%-0.0%	0.0%-1.1%	no coverage	0.1%-0.9%
mdr1-Met239Ile	0/25	0/90	2/478	0/59	0/161	0/78	1/508	0/67	0/216	0/43	0/195	0/313	0/30	3/2263
	0.0%	0.0%	0.4%	0.0%	0.0%	0.0%	0.2%	0.0%	0.0%	0.0%	0.0%	0.0%	0.0%	0.1%
	0.0%-0.0%	0.0%-0.0%	0.0%-0.9%	0.0%-0.0%	0.0%-0.0%	0.0%-0.0%	0.0%-0.5%	0.0%-0.0%	0.0%-0.0%	0.0%-0.0%	0.0%-0.0%	0.0%-0.0%	0.0%-0.0%	0.0%-0.3%
mdr1-Phe1194Leu	no coverage	0/65	1/339	0/53	0/105	0/50	0/462	no coverage	0/108	0/13	0/70	2/289	0/1	3/1555
	no coverage	0.0%	0.3%	0.0%	0.0%	0.0%	0.0%	no coverage	0.0%	0.0%	0.0%	0.7%	0.0%	0.2%
	no coverage	0.0%-0.0%	0.0%-0.8%	0.0%-0.0%	0.0%-0.0%	0.0%-0.0%	0.0%-0.0%	no coverage	0.0%-0.0%	0.0%-0.0%	0.0%-0.0%	0.0%-1.5%	0.0%-0.0%	0.0%-0.4%
mdr1-Phe938Tyr	2/69	1/107	10/484	0/48	1/144	0/105	51/488	0/107	0/200	0/34	0/178	11/310	0/27	76/2301
	2.9%	0.9%	2.1%	0.0%	0.7%	0.0%	10.5%	0.0%	0.0%	0.0%	0.0%	3.5%	0.0%	3.3%
	0.0%-6.2%	0.0%-2.5%	1.0%-3.1%	0.0%-0.0%	0.0%-1.8%	0.0%-0.0%	8.2%-12.7%	0.0%-0.0%	0.0%-0.0%	0.0%-0.0%	0.0%-0.0%	1.8%-5.3%	0.0%-0.0%	2.7%-3.9%
mdr1-Thr199Ser	1/125	0/164	5/629	1/60	0/188	0/165	5/525	0/155	0/272	0/55	0/288	3/380	0/42	15/3048
	0.8%	0.0%	0.8%	1.7%	0.0%	0.0%	1.0%	0.0%	0.0%	0.0%	0.0%	0.8%	0.0%	0.5%
	0.0%-2.1%	0.0%-0.0%	0.2%-1.4%	0.0%-4.4%	0.0%-0.0%	0.0%-0.0%	0.3%-1.6%	0.0%-0.0%	0.0%-0.0%	0.0%-0.0%	0.0%-0.0%	0.0%-1.5%	0.0%-0.0%	0.3%-0.7%
mdr1-Tyr1197Asn	no coverage	0/65	1/339	0/53	0/105	0/50	0/462	no coverage	2/108	0/13	0/70	0/289	0/1	3/1555
	no coverage	0.0%	0.3%	0.0%	0.0%	0.0%	0.0%	no coverage	1.9%	0.0%	0.0%	0.0%	0.0%	0.2%
	no coverage	0.0%-0.0%	0.0%-0.8%	0.0%-0.0%	0.0%-0.0%	0.0%-0.0%	0.0%-0.0%	no coverage	0.0%-4.0%	0.0%-0.0%	0.0%-0.0%	0.0%-0.0%	0.0%-0.0%	0.0%-0.4%
mdr1-Tyr184Phe	65/125	79/164	279/629	19/60	116/188	101/165	336/525	88/155	134/272	33/55	113/288	209/380	15/42	1587/3048
	52.0%	48.2%	44.4%	31.7%	61.7%	61.2%	64.0%	56.8%	49.3%	60.0%	39.2%	55.0%	35.7%	52.1%
	44.6%-59.4%	41.8%-54.6%	41.1%-47.6%	21.8%-41.5%	55.9%-67.5%	55.0%-67.5%	60.6%-67.4%	50.2%-63.3%	44.3%-54.3%	49.1%-70.9%	34.5%-44.0%	50.8%-59.2%	23.6%-47.9%	50.6%-53.6%
mdr1-Val533Ile	0/96	0/115	0/567	0/45	0/143	0/118	2/477	0/103	0/222	0/28	0/229	1/305	0/15	3/2463
	0.0%	0.0%	0.0%	0.0%	0.0%	0.0%	0.4%	0.0%	0.0%	0.0%	0.0%	0.3%	0.0%	0.1%
	0.0%-0.0%	0.0%-0.0%	0.0%-0.0%	0.0%-0.0%	0.0%-0.0%	0.0%-0.0%	0.0%-0.9%	0.0%-0.0%	0.0%-0.0%	0.0%-0.0%	0.0%-0.0%	0.0%-0.9%	0.0%-0.0%	0.0%-0.2%
mdr2-Ile492Val	1/3	25/65	139/254	0/2	47/119	16/52	66/118	2/17	53/127	7/14	39/93	9/19	0/3	404/886
	33.3%	38.5%	54.7%	0.0%	39.5%	30.8%	55.9%	11.8%	41.7%	50.0%	41.9%	47.4%	0.0%	45.6%
	0.0%-78.1%	28.5%-48.4%	49.6%-59.9%	0.0%-0.0%	32.1%-46.9%	20.2%-41.3%	48.4%-63.4%	0.0%-24.6%	34.5%-48.9%	28.0%-72.0%	33.5%-50.4%	28.5%-66.2%	0.0%-0.0%	42.8%-48.4%
pfdpap1-Ala118Ser	0/95	0/136	1/517	0/11	0/158	0/143	2/166	0/102	3/271	0/38	0/314	0/81	0/22	6/2054
	0.0%	0.0%	0.2%	0.0%	0.0%	0.0%	1.2%	0.0%	1.1%	0.0%	0.0%	0.0%	0.0%	0.3%
	0.0%-0.0%	0.0%-0.0%	0.0%-0.5%	0.0%-0.0%	0.0%-0.0%	0.0%-0.0%	0.0%-2.6%	0.0%-0.0%	0.1%-2.2%	0.0%-0.0%	0.0%-0.0%	0.0%-0.0%	0.0%-0.0%	0.1%-0.5%
pfdpap1-Ala2Thr	0/2	24/67	108/258	1/2	40/119	22/53	47/118	8/19	29/126	8/22	36/97	8/17	3/6	334/906
	0.0%	35.8%	41.9%	50.0%	33.6%	41.5%	39.8%	42.1%	23.0%	36.4%	37.1%	47.1%	50.0%	36.9%
	0.0%-0.0%	26.2%-45.5%	36.8%-46.9%	0.0%-100.0%	26.5%-40.7%	30.4%-52.6%	32.4%-47.2%	23.5%-60.7%	16.8%-29.2%	19.5%-53.2%	29.0%-45.2%	27.1%-67.0%	16.4%-83.6%	34.2%-39.5%
pfdpap1-Asn144Thr	0/70	0/119	1/458	0/5	0/132	0/110	0/138	1/86	0/224	0/28	2/235	0/52	0/10	4/1667
	0.0%	0.0%	0.2%	0.0%	0.0%	0.0%	0.0%	1.2%	0.0%	0.0%	0.9%	0.0%	0.0%	0.2%
	0.0%-0.0%	0.0%-0.0%	0.0%-0.6%	0.0%-0.0%	0.0%-0.0%	0.0%-0.0%	0.0%-0.0%	0.0%-3.1%	0.0%-0.0%	0.0%-0.0%	0.0%-1.8%	0.0%-0.0%	0.0%-0.0%	0.0%-0.4%
pfdpap1-Asp295Ala	8/16	17/52	64/239	no coverage	23/70	14/39	44/97	15/59	27/74	3/11	24/83	3/14	1/2	243/756
	50.0%	32.7%	26.8%	no coverage	32.9%	35.9%	45.4%	25.4%	36.5%	27.3%	28.9%	21.4%	50.0%	32.1%
	29.4%-70.6%	22.0%-43.4%	22.1%-31.5%	no coverage	23.6%-42.1%	23.3%-48.5%	37.0%-53.7%	16.1%-34.7%	27.3%-45.7%	5.2%-49.4%	20.7%-37.1%	3.4%-39.5%	0.0%-100.0%	29.3%-34.9%
pfdpap1-Glu497Lys	0/67	0/105	0/434	0/1	0/112	0/95	0/121	0/88	0/188	0/21	4/218	0/61	0/4	4/1515
	0.0%	0.0%	0.0%	0.0%	0.0%	0.0%	0.0%	0.0%	0.0%	0.0%	1.8%	0.0%	0.0%	0.3%

	0.0%-0.0%	0.0%-0.0%	0.0%-0.0%	0.0%-0.0%	0.0%-0.0%	0.0%-0.0%	0.0%-0.0%	0.0%-0.0%	0.0%-0.0%	0.0%-0.0%	0.3%-3.3%	0.0%-0.0%	0.0%-0.0%	0.0%-0.5%
pfdpap1-Glu596Lys	0/46	0/90	0/420	0/7	0/137	0/83	0/141	0/81	3/231	0/36	0/243	0/39	0/9	3/1563
	0.0%	0.0%	0.0%	0.0%	0.0%	0.0%	0.0%	0.0%	1.3%	0.0%	0.0%	0.0%	0.0%	0.2%
	0.0%-0.0%	0.0%-0.0%	0.0%-0.0%	0.0%-0.0%	0.0%-0.0%	0.0%-0.0%	0.0%-0.0%	0.0%-0.0%	0.1%-2.5%	0.0%-0.0%	0.0%-0.0%	0.0%-0.0%	0.0%-0.0%	0.0%-0.4%
pfdpap1-Glu692Gln	no coverage	7/57	22/236	no coverage	13/109	7/48	14/113	no coverage	4/89	0/24	6/67	3/13	0/2	76/758
	no coverage	12.3%	9.3%	no coverage	11.9%	14.6%	12.4%	no coverage	4.5%	0.0%	9.0%	23.1%	0.0%	10.0%
	no coverage	5.1%-19.4%	6.2%-12.4%	no coverage	6.8%-17.0%	6.2%-23.0%	7.3%-17.5%	no coverage	0.9%-8.1%	0.0%-0.0%	3.2%-14.7%	3.9%-42.3%	0.0%-0.0%	8.2%-11.8%
pfdpap1-Glu692Lys	no coverage	0/57	11/236	no coverage	2/109	0/48	8/113	no coverage	5/89	0/24	0/67	0/13	0/2	26/758
	no coverage	0.0%	4.7%	no coverage	1.8%	0.0%	7.1%	no coverage	5.6%	0.0%	0.0%	0.0%	0.0%	3.4%
	no coverage	0.0%-0.0%	2.4%-6.9%	no coverage	0.0%-3.9%	0.0%-0.0%	3.1%-11.0%	no coverage	1.6%-9.6%	0.0%-0.0%	0.0%-0.0%	0.0%-0.0%	0.0%-0.0%	2.3%-4.5%
pfdpap1-Ile636Met	1/17	0/66	0/355	0/2	0/117	0/63	1/123	0/65	1/189	0/30	0/160	1/26	0/4	4/1217
	5.9%	0.0%	0.0%	0.0%	0.0%	0.0%	0.8%	0.0%	0.5%	0.0%	0.0%	3.8%	0.0%	0.3%
	0.0%-15.3%	0.0%-0.0%	0.0%-0.0%	0.0%-0.0%	0.0%-0.0%	0.0%-0.0%	0.0%-2.1%	0.0%-0.0%	0.0%-1.4%	0.0%-0.0%	0.0%-0.0%	0.0%-10.0%	0.0%-0.0%	0.1%-0.6%
pfdpap1-Ile689Lys	no coverage	10/57	40/236	no coverage	13/109	10/48	26/113	no coverage	10/89	1/24	3/67	2/13	0/2	115/758
	no coverage	17.5%	16.9%	no coverage	11.9%	20.8%	23.0%	no coverage	11.2%	4.2%	4.5%	15.4%	0.0%	15.2%
	no coverage	9.3%-25.8%	12.9%-21.0%	no coverage	6.8%-17.0%	11.2%-30.5%	16.5%-29.5%	no coverage	5.7%-16.7%	0.0%-10.9%	0.3%-8.6%	0.0%-31.8%	0.0%-0.0%	13.0%-17.3%
pfdpap1-Leu218Val	0/6	2/71	3/298	0/5	1/135	0/65	0/130	2/25	5/188	0/32	4/134	0/19	0/14	17/1122
	0.0%	2.8%	1.0%	0.0%	0.7%	0.0%	0.0%	8.0%	2.7%	0.0%	3.0%	0.0%	0.0%	1.5%
	0.0%-0.0%	0.0%-6.0%	0.1%-2.0%	0.0%-0.0%	0.0%-2.0%	0.0%-0.0%	0.0%-0.0%	0.0%-16.9%	0.7%-4.6%	0.0%-0.0%	0.6%-5.4%	0.0%-0.0%	0.0%-0.0%	0.9%-2.1%
pfdpap1-Leu690Asn	no coverage	0/57	1/236	no coverage	1/109	1/48	1/113	no coverage	0/89	0/24	0/67	0/13	0/2	4/758
	no coverage	0.0%	0.4%	no coverage	0.9%	2.1%	0.9%	no coverage	0.0%	0.0%	0.0%	0.0%	0.0%	0.5%
	no coverage	0.0%-0.0%	0.0%-1.1%	no coverage	0.0%-2.4%	0.0%-5.5%	0.0%-2.3%	no coverage	0.0%-0.0%	0.0%-0.0%	0.0%-0.0%	0.0%-0.0%	0.0%-0.0%	0.1%-1.0%
pfdpap1-Leu690His	no coverage	2/57	0/236	no coverage	1/109	0/48	2/113	no coverage	1/89	1/24	1/67	0/13	0/2	8/758
	no coverage	3.5%	0.0%	no coverage	0.9%	0.0%	1.8%	no coverage	1.1%	4.2%	1.5%	0.0%	0.0%	1.1%
	no coverage	0.0%-7.5%	0.0%-0.0%	no coverage	0.0%-2.4%	0.0%-0.0%	0.0%-3.8%	no coverage	0.0%-3.0%	0.0%-10.9%	0.0%-3.9%	0.0%-0.0%	0.0%-0.0%	0.4%-1.7%
pfdpap1-Leu690Ile	no coverage	5/57	32/236	no coverage	12/109	4/48	19/113	no coverage	13/89	3/24	5/67	3/13	0/2	96/758
	no coverage	8.8%	13.6%	no coverage	11.0%	8.3%	16.8%	no coverage	14.6%	12.5%	7.5%	23.1%	0.0%	12.7%
	no coverage	2.6%-14.9%	9.9%-17.2%	no coverage	6.1%-15.9%	1.8%-14.9%	11.0%-22.6%	no coverage	8.4%-20.8%	1.4%-23.6%	2.2%-12.7%	3.9%-42.3%	0.0%-0.0%	10.7%-14.7%
pfdpap1-Leu691Phe	no coverage	1/57	11/236	no coverage	1/109	1/48	6/113	no coverage	5/89	0/24	0/67	0/13	0/2	25/758
	no coverage	1.8%	4.7%	no coverage	0.9%	2.1%	5.3%	no coverage	5.6%	0.0%	0.0%	0.0%	0.0%	3.3%
	no coverage	0.0%-4.6%	2.4%-6.9%	no coverage	0.0%-2.4%	0.0%-5.5%	1.8%-8.8%	no coverage	1.6%-9.6%	0.0%-0.0%	0.0%-0.0%	0.0%-0.0%	0.0%-0.0%	2.2%-4.4%
pfdpap1-Lys216Asn	0/6	2/71	3/298	0/5	3/135	3/65	1/130	0/25	2/188	0/32	0/134	0/19	1/14	15/1122
	0.0%	2.8%	1.0%	0.0%	2.2%	4.6%	0.8%	0.0%	1.1%	0.0%	0.0%	0.0%	7.1%	1.3%
	0.0%-0.0%	0.0%-6.0%	0.1%-2.0%	0.0%-0.0%	0.1%-4.3%	0.3%-8.9%	0.0%-2.0%	0.0%-0.0%	0.0%-2.3%	0.0%-0.0%	0.0%-0.0%	0.0%-0.0%	0.0%-18.5%	0.8%-1.9%
pfdpap1-Lys216Thr	0/6	0/71	1/298	0/5	0/135	1/65	0/130	0/25	0/188	0/32	0/134	1/19	0/14	3/1122
	0.0%	0.0%	0.3%	0.0%	0.0%	1.5%	0.0%	0.0%	0.0%	0.0%	0.0%	5.3%	0.0%	0.3%
	0.0%-0.0%	0.0%-0.0%	0.0%-0.9%	0.0%-0.0%	0.0%-0.0%	0.0%-4.0%	0.0%-0.0%	0.0%-0.0%	0.0%-0.0%	0.0%-0.0%	0.0%-0.0%	0.0%-13.7%	0.0%-0.0%	0.0%-0.5%
pfdpap1-Pro161Leu	0/82	4/126	0/484	0/8	1/150	1/120	1/156	0/97	0/259	0/34	2/282	0/61	0/16	9/1875
	0.0%	3.2%	0.0%	0.0%	0.7%	0.8%	0.6%	0.0%	0.0%	0.0%	0.7%	0.0%	0.0%	0.5%
	0.0%-0.0%	0.6%-5.7%	0.0%-0.0%	0.0%-0.0%	0.0%-1.8%	0.0%-2.2%	0.0%-1.7%	0.0%-0.0%	0.0%-0.0%	0.0%-0.0%	0.0%-1.5%	0.0%-0.0%	0.0%-0.0%	0.2%-0.7%
pfdpap1-Ser292Tyr	0/16	2/52	8/239	no coverage	3/70	3/39	7/97	2/59	0/74	0/11	3/83	0/14	0/2	28/756
	0.0%	3.8%	3.3%	no coverage	4.3%	7.7%	7.2%	3.4%	0.0%	0.0%	3.6%	0.0%	0.0%	3.7%
	0.0%-0.0%	0.0%-8.2%	1.4%-5.3%	no coverage	0.3%-8.3%	0.7%-14.7%	2.9%-11.5%	0.0%-7.3%	0.0%-0.0%	0.0%-0.0%	0.2%-7.0%	0.0%-0.0%	0.0%-0.0%	2.6%-4.8%
pfdpap1-Ser509Asn	0/67	0/105	0/434	0/1	1/112	0/95	1/121	2/88	2/188	0/21	0/218	0/61	0/4	6/1515
	0.0%	0.0%	0.0%	0.0%	0.9%	0.0%	0.8%	2.3%	1.1%	0.0%	0.0%	0.0%	0.0%	0.4%
	0.0%-0.0%	0.0%-0.0%	0.0%-0.0%	0.0%-0.0%	0.0%-2.4%	0.0%-0.0%	0.0%-2.2%	0.0%-4.9%	0.0%-2.3%	0.0%-0.0%	0.0%-0.0%	0.0%-0.0%	0.0%-0.0%	0.1%-0.7%
pfdpap1-Thr222Ile	no coverage	1/66	0/248	0/2	2/114	1/55	2/114	0/2	1/131	0/23	0/98	0/16	0/2	7/871
	no coverage	1.5%	0.0%	0.0%	1.8%	1.8%	1.8%	0.0%	0.8%	0.0%	0.0%	0.0%	0.0%	0.8%
	no coverage	0.0%-4.0%	0.0%-0.0%	0.0%-0.0%	0.0%-3.8%	0.0%-4.8%	0.0%-3.8%	0.0%-0.0%	0.0%-2.0%	0.0%-0.0%	0.0%-0.0%	0.0%-0.0%	0.0%-0.0%	0.3%-1.3%
pfdpap1-Thr327Ser	1/54	0/92	0/389	0/9	1/128	1/87	1/128	0/87	0/179	0/30	2/186	1/35	0/12	7/1416

	1.9%	0.0%	0.0%	0.0%	0.8%	1.1%	0.8%	0.0%	0.0%	0.0%	1.1%	2.9%	0.0%	0.5%
	0.0%-4.9%	0.0%-0.0%	0.0%-0.0%	0.0%-0.0%	0.0%-2.1%	0.0%-3.0%	0.0%-2.1%	0.0%-0.0%	0.0%-0.0%	0.0%-0.0%	0.0%-2.3%	0.0%-7.5%	0.0%-0.0%	0.2%-0.8%
pfdpap1-Val523Ile	0/67	1/105	0/434	0/1	2/112	2/95	0/121	0/88	4/188	0/21	0/218	0/61	0/4	9/1515
	0.0%	1.0%	0.0%	0.0%	1.8%	2.1%	0.0%	0.0%	2.1%	0.0%	0.0%	0.0%	0.0%	0.6%
	0.0%-0.0%	0.0%-2.5%	0.0%-0.0%	0.0%-0.0%	0.0%-3.8%	0.0%-4.5%	0.0%-0.0%	0.0%-0.0%	0.4%-3.9%	0.0%-0.0%	0.0%-0.0%	0.0%-0.0%	0.0%-0.0%	0.3%-0.9%
pph-Asp1147Asn	0/7	0/60	6/230	no coverage	2/96	0/50	1/103	0/24	2/155	0/24	0/101	0/17	0/2	11/869
	0.0%	0.0%	2.6%	no coverage	2.1%	0.0%	1.0%	0.0%	1.3%	0.0%	0.0%	0.0%	0.0%	1.3%
	0.0%-0.0%	0.0%-0.0%	0.9%-4.3%	no coverage	0.0%-4.5%	0.0%-0.0%	0.0%-2.6%	0.0%-0.0%	0.0%-2.8%	0.0%-0.0%	0.0%-0.0%	0.0%-0.0%	0.0%-0.0%	0.6%-1.9%
pph-Glu1097Lys	0/7	3/60	0/230	no coverage	0/96	3/50	0/103	0/24	0/155	0/24	0/101	0/17	0/2	6/869
	0.0%	5.0%	0.0%	no coverage	0.0%	6.0%	0.0%	0.0%	0.0%	0.0%	0.0%	0.0%	0.0%	0.7%
	0.0%-0.0%	0.4%-9.6%	0.0%-0.0%	no coverage	0.0%-0.0%	0.5%-11.5%	0.0%-0.0%	0.0%-0.0%	0.0%-0.0%	0.0%-0.0%	0.0%-0.0%	0.0%-0.0%	0.0%-0.0%	0.2%-1.2%
pph-Met1146Lys	0/7	1/60	1/230	no coverage	0/96	0/50	2/103	0/24	0/155	0/24	0/101	0/17	0/2	4/869
	0.0%	1.7%	0.4%	no coverage	0.0%	0.0%	1.9%	0.0%	0.0%	0.0%	0.0%	0.0%	0.0%	0.5%
	0.0%-0.0%	0.0%-4.4%	0.0%-1.1%	no coverage	0.0%-0.0%	0.0%-0.0%	0.0%-4.2%	0.0%-0.0%	0.0%-0.0%	0.0%-0.0%	0.0%-0.0%	0.0%-0.0%	0.0%-0.0%	0.1%-0.8%
pph-Ser1095Ile	0/7	0/60	0/230	no coverage	1/96	0/50	1/103	0/24	2/155	0/24	0/101	0/17	0/2	4/869
	0.0%	0.0%	0.0%	no coverage	1.0%	0.0%	1.0%	0.0%	1.3%	0.0%	0.0%	0.0%	0.0%	0.5%
	0.0%-0.0%	0.0%-0.0%	0.0%-0.0%	no coverage	0.0%-2.7%	0.0%-0.0%	0.0%-2.6%	0.0%-0.0%	0.0%-2.8%	0.0%-0.0%	0.0%-0.0%	0.0%-0.0%	0.0%-0.0%	0.1%-0.8%

Study Regions

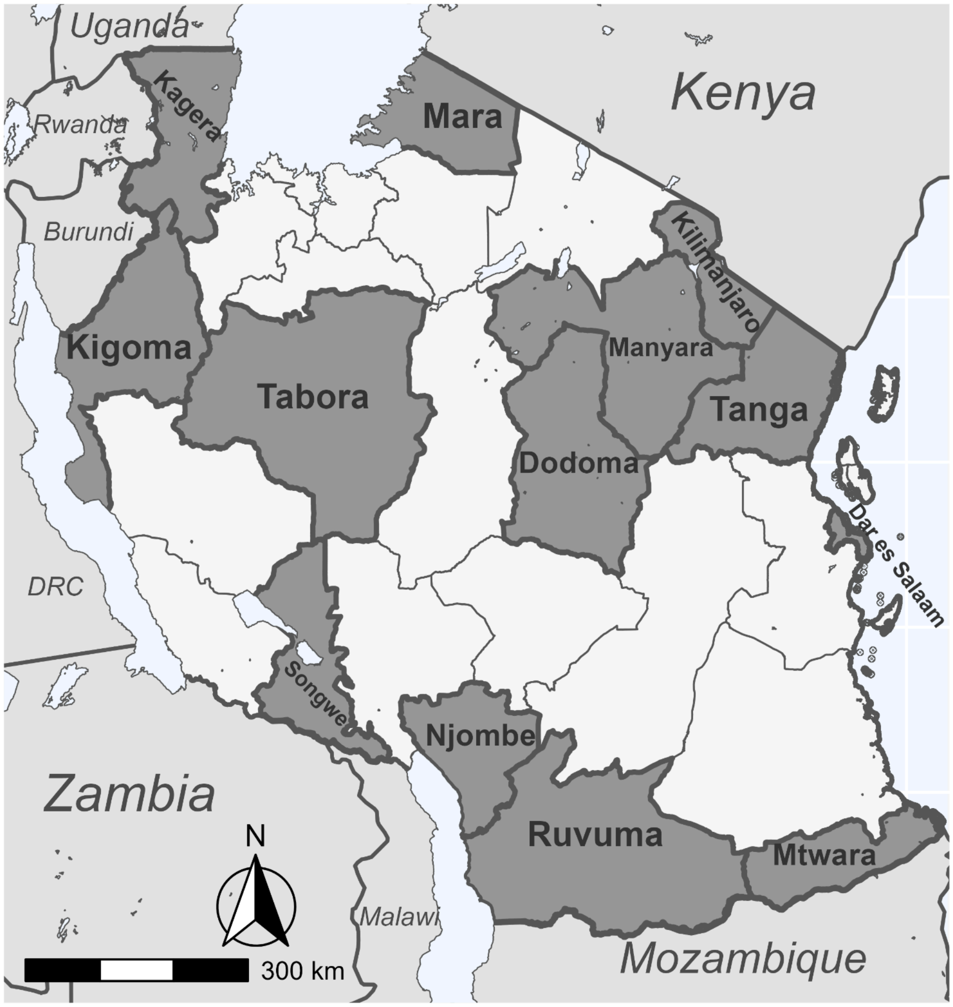

Figure S1. Regions with sampling in the study. The regions of Tanga, Ruvuma, and Kigoma involved community survey samples. The remaining regions involved sample collections in health facilities.

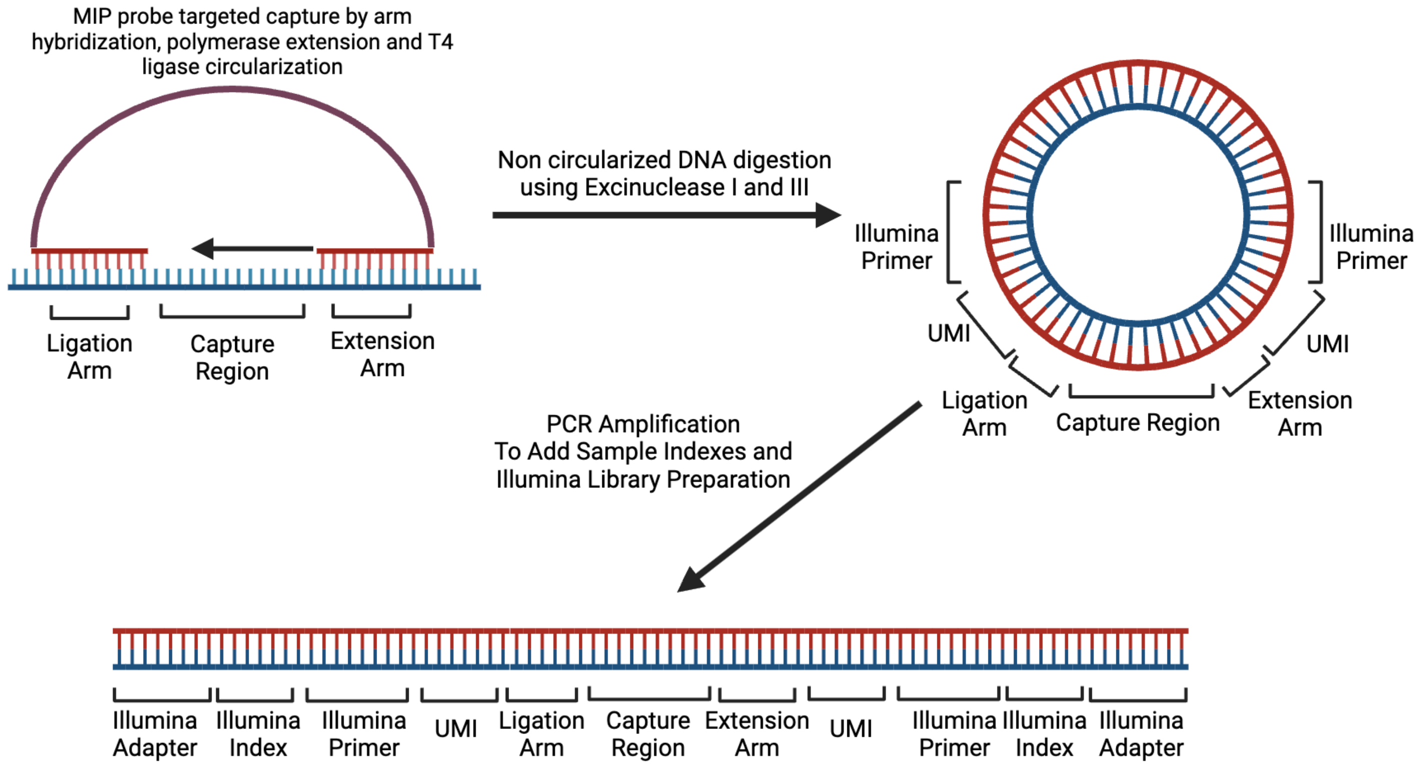

Figure S2. Schematic of Molecular Inversion Probe Method. UMI: Unique Molecular Identifier. The 3'-end of the molecular inversion probe ends in the extension arm where polymerase primes and extends while the 5' end of the MIP represents the ligation arm where after extension the circle is ligated closed. Both arms hybridized based on complementary sequence to the sequence flanking the target. Generated with Biorender academic license to University of North Carolina**.**

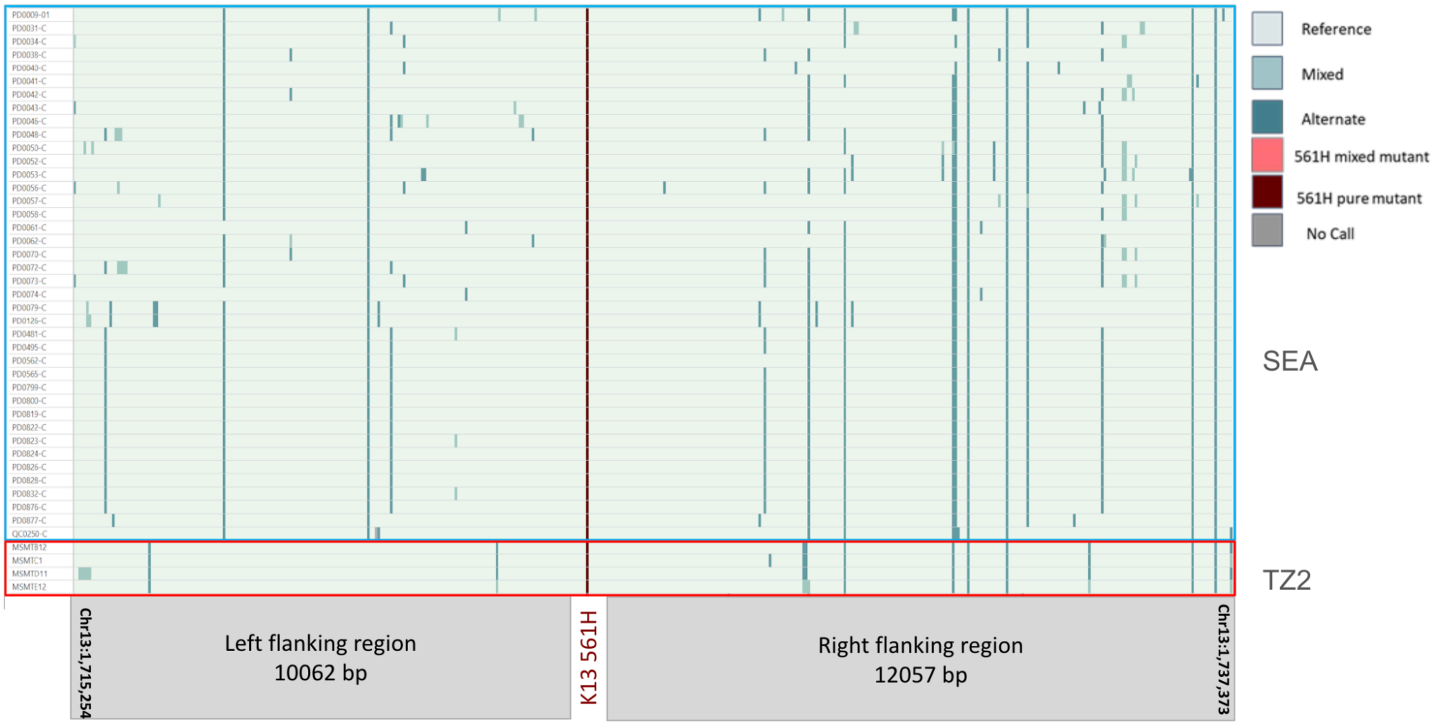

Figure S3. Extended haplotype comparison between TZ2 and Southeast Asian (SEA) isolates. R561**H** mutant parasites collected in SEA from Pf7K (blue box) are compared to TZ2 isolates (red box) from this study. Extended haplotypes show two distinct groups.

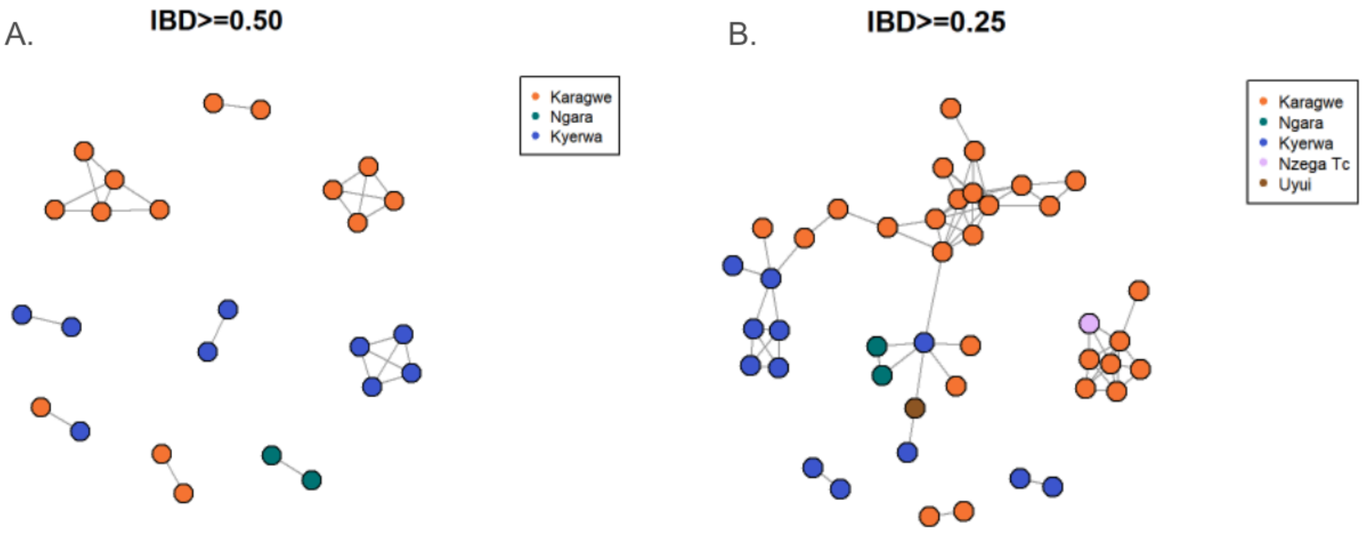

Figure S4. Inheritance by descent relatedness among R561H parasite infection in Kagera. IBD ranges from 0 (completely unrelated) to 1 (identical) for infections. Panel A and B show networks of parasites with 0.5 and 0.25 IBD, respectively. Isolates are colored by the district that they are from.

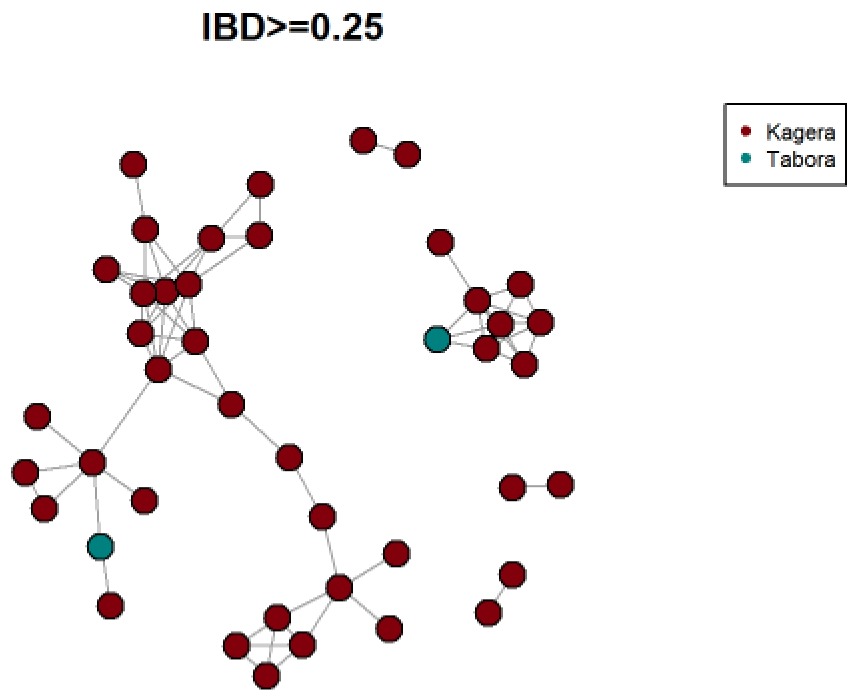

Figure S5. IBD relatedness of R561H infections between regions. At an IBD level of 0.25, the two parasite isolates from Tabora cluster with isolates from Kagera. The isolates in other regions remain not connected to the network demonstrating lower levels of relatedness.

REFERENCES

1. Popkin Hall ZR, Seth MD, Madebe RA, Budodo R, Bakari C, Francis F, et al. Malaria species positivity rates among symptomatic individuals across regions of differing transmission intensities in Mainland Tanzania. J Infect Dis [Internet]. 2023 Nov 22; Available from: http://dx.doi.org/10.1093/infdis/jiad522

2. Rogier E, Battle N, Bakari C, Seth MD, Nace D, Herman C, et al. Plasmodium falciparum pfhrp2 and pfhrp3 gene deletions among patients enrolled at 100 health facilities throughout Tanzania: February to July 2021 [Internet]. medRxiv. 2023 [cited 2024 Mar 14]. p. 2023.07.29.23293322. Available from: https://www.medrxiv.org/content/10.1101/2023.07.29.23293322v1.abstract

3. Popkin-Hall ZR, Seth MD, Madebe RA, Budodo R, Bakari C, Francis F, et al. Prevalence of non-falciparum malaria infections among asymptomatic individuals in four regions of Mainland Tanzania. Parasit Vectors. 2024 Mar 23;17(1):153.

4. Ishengoma DS, Mmbando BP, Mandara CI, Chiduo MG, Francis F, Timiza W, et al. Trends of Plasmodium falciparum prevalence in two communities of Muheza district North-eastern Tanzania: correlation between parasite prevalence, malaria interventions and rainfall in the context of re-emergence of malaria after two decades of progressively declining transmission. Malar J. 2018 Jul 6;17(1):252.

5. Moser KA, Madebe RA, Aydemir O, Chiduo MG, Mandara CI, Rumisha SF, et al. Describing the current status of Plasmodium falciparum population structure and drug resistance within mainland Tanzania using molecular inversion probes. Mol Ecol [Internet]. 2020 Oct 26; Available from: http://dx.doi.org/10.1111/mec.15706

6. Fola AA, Feleke SM, Mohammed H, Brhane BG, Hennelly CM, Assefa A, et al. Plasmodium falciparum resistant to artemisinin and diagnostics have emerged in Ethiopia. Nat Microbiol. 2023 Oct;8(10):1911–9.

7. Verity R, Aydemir O, Brazeau NF, Watson OJ, Hathaway NJ, Mwandagalirwa MK, et al. The impact of antimalarial resistance on the genetic structure of Plasmodium falciparum in the DRC. Nat Commun. 2020 Apr 30;11(1):2107.

8. Oyola SO, Ariani CV, Hamilton WL, Kekre M, Amenga-Etego LN, Ghansah A, et al. Whole genome sequencing of Plasmodium falciparum from dried blood spots using selective whole genome amplification. Malar J. 2016 Dec 20;15(1):597.

9. Miotto O, Amato R, Ashley EA, MacInnis B, Almagro-Garcia J, Amaratunga C, et al. Genetic architecture of artemisinin-resistant Plasmodium falciparum. Nat Genet. 2015 Mar;47(3):226–34.

10. Ross LS, Fidock DA. Elucidating Mechanisms of Drug-Resistant Plasmodium falciparum. Cell Host Microbe. 2019 Jul 10;26(1):35–47.

11. Rosenthal PJ, Asua V, Conrad MD. Emergence, transmission dynamics and mechanisms of artemisinin partial resistance in malaria parasites in Africa. Nat Rev Microbiol [Internet]. 2024 Feb 6; Available from: http://dx.doi.org/10.1038/s41579-024-01008-2

12. Blasco B, Leroy D, Fidock DA. Antimalarial drug resistance: linking Plasmodium falciparum parasite biology to the clinic. Nat Med. 2017 Aug 4;23(8):917–28.

13. Website [Internet]. Available from: https://iris.who.int/bitstream/handle/10665/336692/9789240012813-eng.pdf?sequence=1
